# Promotion of axon regeneration and protection on injured retinal ganglion cells by rCXCL2

**DOI:** 10.1186/s41232-023-00283-5

**Published:** 2023-06-20

**Authors:** Zi-Yuan Zhang, Zhao-Yang Zuo, Yang Liang, Si-Ming Zhang, Chun-Xia Zhang, Jing Chi, Bin Fan, Guang-Yu Li

**Affiliations:** Department of Ophthalmology, Second Hospital of Jilin University, Changchun, 130041 China

**Keywords:** CXCL2, Axon regeneration, Retinal ganglion cells, Neuroprotection, Chemokine

## Abstract

**Background:**

In addition to rescuing injured retinal ganglion cells (RGCs) by stimulating the intrinsic growth ability of damaged RGCs in various retinal/optic neuropathies, increasing evidence has shown that the external microenvironmental factors also play a crucial role in restoring the survival of RGCs by promoting the regrowth of RGC axons, especially inflammatory factors. In this study, we aimed to screen out the underlying inflammatory factor involved in the signaling of staurosporine (STS)-induced axon regeneration and verify its role in the protection of RGCs and the promotion of axon regrowth.

**Methods:**

We performed transcriptome RNA sequencing for STS induction models in vitro and analyzed the differentially expressed genes. After targeting the key gene, we verified the role of the candidate factor in RGC protection and promotion of axon regeneration in vivo with two RGC-injured animal models (optic nerve crush, ONC; retinal *N*-methyl-*D*-aspartate, NMDA damage) by using cholera toxin subunit B anterograde axon tracing and specific immunostaining of RGCs.

**Results:**

We found that a series of inflammatory genes expressed upregulated in the signaling of STS-induced axon regrowth and we targeted the candidate *CXCL2* gene since the level of the chemokine *CXCL2* gene elevated significantly among the top upregulated genes. We further demonstrated that intravitreal injection of rCXCL2 robustly promoted axon regeneration and significantly improved RGC survival in ONC-injured mice in vivo. However, different from its role in ONC model, the intravitreal injection of rCXCL2 was able to simply protect RGCs against NMDA-induced excitotoxicity in mouse retina and maintain the long-distance projection of RGC axons, yet failed to promote significant axon regeneration.

**Conclusions:**

We provide the first in vivo evidence that CXCL2, as an inflammatory factor, is a key regulator in the axon regeneration and neuroprotection of RGCs. Our comparative study may facilitate deciphering the exact molecular mechanisms of RGC axon regeneration and developing high-potency targeted drugs.

**Supplementary Information:**

The online version contains supplementary material available at 10.1186/s41232-023-00283-5.

## Background

As the sole output neurons in the retina, retinal ganglion cells (RGCs) transmit visual impulses to the brain to form vision [1]. The damage of RGCs accompanied by the anterograde or retrograde loss of RGC axons is a characteristic pathological change in various retinal/optic neuropathies, such as glaucoma, retinal ischemia, and traumatic optic neuropathy, which are all able to cause visual dysfunction and even irreversible blindness [2–4]. Therefore, to efficiently preserve the visual function for these diseases, in-depth investigation on the molecular mechanism of protection on RGCs and promotion of axon regeneration will be of great scientific significance.

The classic strategies of optic neuroprotection mainly focus on how to delay or block the programmed death of RGCs by regulating different signaling pathways. For instance, it has been reported that overexpression of the *Sirt-1* gene in RGCs via adeno-associated virus 2 (AAV2) significantly protected rat RGCs from optic nerve crush (ONC) injury [5], and reactivation of CaMKII via AAV gene therapy robustly protected RGCs against murine ONC/*N*-methyl-D-aspartate (NMDA) damage [6]. However, the therapeutic strategies centered on rescuing RGCs still cannot reverse the visual dysfunction caused by the massive death of RGCs; thus, how to promote the regeneration of RGCs has become a novel research hotspot. Cell replacement therapy has made significant progress in the past several years. For example, RGC-like precursor neurons that were differentiated in vitro from embryonic stem cells or induced pluripotent stem cells were implanted into the eye and successfully formed neuronal synaptic connections with upper neurons [7, 8]. More recently, Zhou et al*.* reported that specifically knocking down the *Ptbp1* gene by CRISPR/cas13 system was able to reactivate stem cell-like potential and directly trans-differentiate retinal Müller cells into functional RGCs in vivo [9]. Though the cell replacement therapy offers promising research directions to investigate the regeneration of functional RGCs, there is still a long journey to overcome the technical and safety barriers to eventually achieve clinical application.

Currently, there is increasing evidence that promoting axon regeneration is crucial to achieve the protection of damaged RGCs. Philip and others have found that almost all molecular mechanisms that can stimulate axon regeneration show protective effects on RGCs, but the simple neuroprotection scheme cannot avoid the final fate of RGCs’ death caused by axon injury [10, 11]. Regenerating axons require the sprouting of growth cones, which involves resealing the ruptured cell membrane, reorganizing the intra-axonal organelles, and remodeling the microtubules and other cytoskeletal components [12]. A determinant of axon regeneration is the intrinsic growth ability of injured neurons, which is regulated by specific genes. For example, Xin reported that knocking down the *PTEN* gene, which is an inhibitory factor of the phosphatidylinositol 3-kinase (PI3K)/protein kinase B (PKB/AKT)/mammalian target of rapamycin (mTOR) signaling pathway, significantly promoted the axon regeneration in αRGC cells, and regenerated axons even reached the thalamus to form synapses after 12 weeks [13]. Norsworthy et al. showed that overexpression of *Sox11* markedly promoted axon regeneration of non-αRGCs in mice with ONC injury [14]. Additionally, Lu et al. found that ectopic expression of Oct4, Sox2, and Klf4 genes in mouse RGCs promoted axon regeneration after glaucoma injury by reprogramming the epigenetic signature into youthful DNA methylation patterns and transcriptomes [15]. In addition, the external microenvironmental factors also play a crucial role during the axon regeneration of RGCs, especially inflammatory factors. Inflammatory stimulation in the rat eyes by intravitreal injection of β/γ-crystallins, zymosan, or the toll-like receptor 2 agonist Pam(3)Cys delays axotomy-induced RGC death and transforms RGCs into an active regenerative state [16, 17]. The application of IL-6-like cytokines or continuous expression of hyper-IL-6 directly targeting the gp130 receptor, a common signaling receptor of all IL-6-like cytokines, induces stronger RGC axon regeneration than other known axon growth-promoting treatments such as *PTEN* knockout both in vitro and in vivo [18, 19].

Chemokines, a class of inflammatory factors, are small cytokines or signaling proteins secreted by cells that induce targeted chemotaxis of nearby responding cells. Most chemokines have a small molecular weight (between 8 and 10 kDa) and share a high similarity of gene sequence and amino acid sequence [20]. The formation of growth cones during axon regeneration requires the guidance by chemokines, while growing axons also need to be navigated by responding to these guidance cues [21]; thus, chemokines play an important role in promoting axon regeneration [10, 22, 23].

Staurosporine (STS), functioning as a broad-spectrum protein kinase C (PKC) inhibitor, was originally isolated from the culture medium of *Streptomyces staurosporesa* [24]. Although previous studies have shown that STS can induce cell apoptosis at the micromolar concentration level, more intriguing studies have also verified that low concentrations of STS can significantly induce neurite outgrowth in many neurons derived from different origins, such as HN33 hippocampal cells, RGC-5 retinal cells, PC12 chromocytoma cells, and SH-SY5Y neuroblastoma cells [25–27], suggesting that STS treatment may trigger an unknown molecular mechanism of axon growth commonly existing in neurons.

In this research, we explored the potential molecular mechanism of STS-induced axon regeneration with two types of retinal cells (661W cells, retinal precursor neurons derived from mouse retinal tumors [28]; and ARPE-19 cells, derived from the human retinal pigment epithelium [29]). With whole-transcriptome sequencing (RNA-Seq), we found that the chemokine *CXCL2* gene was one of the top upregulated genes induced by STS in both types of cells and that recombinant CXCL2 (rCXCL2) played a similar role of STS treatment in promoting axon regeneration in 661W cells in vitro. More importantly, we further verified the role of rCXCL2 in the protection of RGCs and the promotion of axon regeneration with two RGC-injured mice models (ONC and NMDA-induced retinal excitotoxicity) in vivo. Our results provide in vivo evidence that CXCL2, as an inflammatory factor, is a key regulator in the axon regeneration and neuroprotection of RGCs, which may be a promising therapeutic target for retinal/optic neuropathies characterized by RGC degeneration.

## Methods

### Chemicals and reagents

Staurosporine (Cat# M2066) and *N*-methyl-d-aspartic acid (NMDA, Cat# M2884) were acquired from AbMole (Beijing, China); 4’,6-diamidino-2-phenylindole (DAPI, Cat# C0065), antifading mounting medium (Cat# S2100), and Triquick Reagent (Trizol Substitute, Cat# R1100) were purchased from Solarbio (Beijing, China); Cholera Toxin Subunit B (CTB) conjugated Alexa Fluor 488 (Cat# C34775) was obtained from Thermo Fisher Scientific; and murine rCXCL2 (Cat# 250–15) was acquired from PeproTech (Rocky Hill, NJ, USA).

### Cell culture

The 661W cell line was provided by Dr. Muayyad Al-Ubaidi (University of Oklahoma Health Sciences Center, USA). The ARPE-19 cell line was purchased from the American Type Culture Collection (ATCC Cat# CRL-2302, RRID: CVCL_0145, USA). Cell culture medium and all supplements were purchased from the HyClone Company (Beijing, China). The 661W cells were maintained in Dulbecco’s modified Eagle’s medium (DMEM) with 10% fetal bovine serum (FBS), 100 U/ml penicillin, and 100 mg/ml streptomycin. The ARPE-19 cells were grown in DMEM/nutrient mixture F-12 (DMEM/F-12) containing 10% FBS, 100 U/ml penicillin, and 100 mg/ml streptomycin. The cells were passaged by 0.05% trypsin–EDTA every 2 or 3 days and cultured in a humidified incubator with 5% CO_2_ and 95% air at 37 °C.

### Cell differentiation with STS

STS was added to cells cultured in 6- or 96-well plates to a final concentration of 75 nM, and solvent dimethyl sulfoxide (0.05% in medium) was used to treat cells as vehicle controls. After 6 h of treatment, the cells were obtained for the experiment described below.

### Cell immunofluorescence

Cells in 96-well plates were fixed with 4% paraformaldehyde (PFA) for 20 min at room temperature (RT). Fixed cells were then rinsed, permeabilized with 0.2% Triton X-100 for 10 min, blocked with 3% bovine serum albumin (BSA) for 30 min, stained with appropriate primary antibodies, and incubated overnight at 4 °C. After washing three times with PBS for 5 min each time, the respective secondary antibodies were added and the cells were incubated at RT for 1 h in the dark. Next, the cells were washed three times with PBS for 5 min each and stained with 0.5 mg/ml DAPI at RT for 10 min. After another triple washing, the images were photographed under an inverted microscope (Olympus, Japan). The fluorescence-positive neurites were traced and graphed using the filament tracing function of Imaris software (v.9.0.1, Bitplane).

The following antibodies were used: mouse anti-β-III Tubulin (1:100, Beyotime Cat# AT809, RRID: AB_2893434); rabbit anti-MAP2 (1:50, Signalway Cat# 21636, RRID: AB_2923039); rabbit anti-NeuN (1:400, Abcam Cat# ab177487, RRID: AB_2532109); Cy3, anti-mouse IgG (1:400, Abbkine Cat# A22210, RRID: AB_2923040); Cy3, anti-rabbit IgG (1:400, Abbkine Cat# A22220, RRID: AB_2923041); and DyLight 488, anti-rabbit IgG (1:200, Abbkine Cat# A23220, RRID: AB_2737289).

### Western blot analysis

Cell and retina samples were sonicated in protein lysate buffer (Beyotime, Shanghai, China), and a bicinchoninic acid assay was used to measure the total protein concentration. Briefly, an equal amount (20 μg) of cell lysate was dissolved in a sample buffer, before boiling the samples for 5 min at 100°C. Next, proteins were separated by sodium dodecyl sulfate–polyacrylamide gel electrophoresis and then transferred onto polyvinylidene fluoride membranes, which were blocked subsequently with 5% nonfat dry milk in Tris-buffered saline with 0.1% Tween-20 (TBS-T) for 1 h at RT. Then, the blots were incubated with the specific primary antibody overnight at 4°C, followed by 1 h of incubation with the appropriate peroxidase-linked secondary antibodies at RT. Signals were then developed using enhanced chemiluminescence, after which images were detected microscopically using a charge-coupled device camera (Tanon, Shanghai, China). Finally, the band density of proteins was calculated with the ImageJ software (v1.53, NIH, USA).

The following antibodies were used: mouse anti-β-III Tubulin (1:2000, Beyotime Cat# AT809, RRID: AB_2893434); rabbit anti-MAP2 (1: 2000 Signalway Cat# 21,636, RRID: AB_2923039); rabbit anti-NeuN (1: 6000, Abcam Cat# ab177487, RRID: AB_2532109); mouse anti-β-actin (1:10000, Signalway Cat# 21800, RRID: AB_2923042); mouse anti-p-STAT3 (1:2000, Santa Cruz Biotechnology Cat# sc-8059, RRID:AB_628292); rabbit anti-p-JAK2 (1:2000, Beyotime Cat# AF1486, RRID: AB_2923043); rabbit anti-Bax (1:1000, Beyotime Cat# AF0057, RRID: AB_2923045); rabbit anti-Bcl-2 (1:1000, Beyotime Cat# AF0060, RRID: AB_2923046); rabbit anti-Caspase-3 (1:1000, Beyotime Cat# AC030, RRID: AB_2923047); anti-rabbit IgG, HRP conjugated (1:8000, Signalway Cat# L3012, RRID: AB_895483); and anti-mouse IgG, HRP conjugated (1:8000, Signalway Cat# L3032, RRID: AB_895481).

### RNA-sequencing

Four groups of cell samples containing Vehicle_661W, STS_661W, Vehicle-ARPE-19, and STS_ARPE-19 were prepared for RNA-Seq and each group had three biological replicates. Total RNA was extracted from the tissues using TRIzol according to the instructions of the manufacturers (Invitrogen, Carlsbad, CA, USA). Subsequently, the total RNA was qualified and quantified using a NanoDrop and Agilent 2100 bioanalyzer (Thermo Fisher Scientific, MA, USA). Oligo (dT)-attached magnetic beads were then used to purify mRNA. Then, cDNA fragments were generated using random hexamer-primed reverse transcription and amplified by polymerase chain reaction (PCR), after which the products were purified by Ampure XP Beads and dissolved in ethidium bromide solution. Agilent Technologies 2100 bioanalyzer was used to validate the double-stranded PCR products, which were then heated, denatured, and circularized by the splint oligo sequence to obtain the final library. The single-strand circle (ssCir) DNA was formatted as the final library. The final library was amplified with phi29 to make DNA nanoball (DNB), which had more than 300 copies of one molecule. DNBs were loaded into the patterned nanoarray, and single-end 50 base reads were generated on the BGIseq500 platform (BGI-Shenzhen, China).

The sequencing data were filtered with SOAPnuke (v1.5.2) by removing reads containing a sequencing adapter, reads whose low-quality base ratio (base quality less than or equal to 5) is more than 20% or whose unknown base (‘N’ base) ratio is more than 5%. Afterwards, clean reads were obtained and stored in FASTQ format. The clean reads were mapped to the reference genome using HISAT2 (v2.0.4). Bowtie2 (v2.2.5) was applied to align the clean reads to the reference coding gene set. Then, the expression level of the gene was calculated by RSEM (v1.2.12). Essentially, differential expression analysis was performed using the DESeq2 (v1.4.5) with a *Q* value ≤ 0.05. Differential expression data are reported as log_2_ (STS_samples/Vehicle_samples).

### qRT-PCR analysis

RNA of STS-treated or vehicle-treated cells was extracted with an Eastep™ Super Total RNA Extraction Kit (Promega, Beijing, China), and cDNA synthesis was performed using the FastQuant RT Kit (TIANGEN, Beijing, China). Real-time PCR was conducted with 2X SG Fast qPCR Master Mix Kit (Sangon Biotech, Shanghai, China), and the primers were as follows: mouse CXCL2-forward (F): AAGTTTGCCTTGACCCTGAA; mouse CXCL2-reverse (R): AGGCACATCAGGTACGATCC; human CXCL2-forward (F): CTGCGCCCAAACCGAAGTCATA; human CXCL2-reverse (R): TTCAGGAACAGCCAATAAGC; mouse GAPDH-F: TGTGTCCGTCGTGGATCTGA; mouse GAPDH-R: TTGCTGTTGAAGTCGCAGGAG; human GAPDH-F: GAGCCCGCAGCCTCCCGCTT; human GAPDH-R: CCCGCGGCCATCACGCCACAG (Sangon Biotech, Shanghai, China). qRT-PCR results are based on four or five replicates. The relative expression in each STS-treated sample was first normalized by the level of GAPDH and then by the value of the vehicle-treated group.

### Animals

Adult male C57BL/6 J mice (age: 8–10 weeks; average weight: 20–23 g) were purchased from the Animal Center of Jilin University (Changchun, China) and were maintained with a free intake of food and water; the indoor temperature was maintained at 21–23°C, with a standard 12-h/12-h light/dark cycle was guaranteed. All of the animal experiments were conducted in accordance with the Association of Research in Vision and Ophthalmology statement regarding the use of animals in ophthalmology and vision research, and they were approved by the Animal Experimentation Ethics Committee of Jilin University.

### Optic nerve crush

Adult mice were anesthetized with pentobarbital sodium (50 mg/kg) by intraperitoneal injection and were placed under the operating microscope. Oxybuprocaine hydrochloride ophthalmic solution (0.5%; Santen, Suzhou, China) was used for topical anesthesia. Following the blunt separation of the conjunctival sac using two toothed forceps around the lateral canthus, the optic nerve of the left eye was carefully exposed intra-orbitally and crushed with self-closing Dumont forceps #5 (Beijing, China) at 1 mm behind the globe for 15 s without damaging the ophthalmic artery. Ophthalmic ointment was applied to protect the cornea after surgery.

### Intravitreal injection

After anesthesia, the pupils of mice were dilated with 5% phenylephrine and 0.5% tropicamide eye drops (Santen, Suzhou, China). Next, a 32-G needle was inserted obliquely downward to make a scleral puncture approximately 1–1.5 mm posterior to the limbus. Consequently, a 10-μl Hamilton syringe connected to a 33-G needle (Hamilton Company, Reno, NV) was inserted into the intravitreal cavity through the scleral tunnel; 1.5 μl of NMDA (20 mM), CTB, vehicle (phosphate buffer saline-PBS), or rCXCL2 (1.0 μg/μl) for each time was injected slowly into the intravitreal space. Special care was given to avoid infringing the lens. The antibiotic ophthalmic ointment was applied to prevent potential infection at the end of the procedure. Any eyes with cataracts and/or hemorrhages were excluded from the analysis. For the ONC model, rCXCL2 and CTB were only injected into the left eyes of mice, while for the NMDA injury model, the left eyes of mice were injected with NMDA and rCXCL2 and the left and right eyes of mice were both injected with CTB 3 days before euthanasia. The maintained axon projection in LGN and SC of the right hemisphere of each group of NMDA-damaged mice was comparatively analyzed by referring to their left hemisphere, where the CTB fluorescence intensity was always kept close to that of the normal control group (no intravitreal injections of NMDA or rCXCL2 in normal control eyes).

### Histology

Before staining mice tissues, the mice were perfused transcardially with saline and 4% PFA. Next, the eyes, optic nerves, optic chiasm, and brains were dissected carefully and completely, after which they were post-fixed in 4% PFA for 24 h and immersed in 30% sucrose for another 24 h.

Whole-mount retinas were then permeabilized with 0.5% Triton X-100 for 30 min and blocked in a mixture of 10% normal goat serum, 0.1% Triton X-100, and 1%BSA in PBS for 1 h. Retinas were incubated with primary antibodies overnight at 4°C for 2 days and washed 3 × 10 min with PBS before 1.5 h of incubation with secondary antibodies at RT. Retinas were again washed 3 × 10 min with PBS and then observed on the slides with a mounting medium. The whole retina was photomerged from several images at low magnification using Adobe Photoshop software. Twelve images (0.35 × 0.45 mm^2^ each) at high magnification were taken for each retina using a fluorescence microscope (Olympus, Japan). The number of RGCs in each image was counted by three researchers blinded to the experiment using the StarDist 2D plugin in ImageJ software, and the average number of RGCs was determined.

For preparing ON and retinal sections, ONs and enucleated eyes were embedded in optimal cutting temperature compound and frozen-sectioned using a cryostat. For immunofluorescent staining, the cryo-Sects. (10 μm for retinal and longitudinal ON sections and 4 μm for cross ON sections) were blocked in a mixture of 10% normal goat serum, 0.1% Triton X-100, and 1%BSA in PBS for 1 h after first washing with PBS. The sections were then incubated with primary antibodies at 4°C overnight, followed by 1 h of incubation with secondary antibodies at RT. After incubation with antibodies, frozen sections were extensively washed three times (5 min per wash) with PBS at RT and mounted in a mounting medium. For terminal-deoxynucleotidyl-transferase (TdT)-mediated dUTP-biotin nick end labeling (TUNEL) assays, the retinal sections were stained using a One Step TUNEL Assay Kit (Beyotime, Shanghai, China) according to the manufacturer’s instructions. The sections were visualized on a fluorescent microscope (Olympus, Japan). The counted number of cells was normalized to the retinal length. The regenerating axons of ONC models in longitudinal ON sections were quantified in four to eight sections per case at prespecified distances from the injury site. The total number of regenerating axons at each site was calculated with the following formula: Σad = πr^2^ × (average axons/mm width)/t. The width at every site was measured (*r* is the radius of the nerve, and *t* is the section thickness). The ON cross-sections of the NMDA models were stained to calculate the number of β-III Tubulin–positive axons at 0.5–1 mm from the eyeball, and five square areas (30 × 30 μm) were sampled in each section for analysis. Three participants measured the number of β-III Tubulin–positive axons to ensure the objective of the experimental data.

The brains of mice were collected 3 days after CTB intravitreal injection as described above and frozen sectioned (15 μm) coronally at LGN and SC. The sections were mounted prior to photographing microscopically (Olympus, Japan).

The following antibodies were used: rabbit anti-RBPMS (1:200, OmnimAbs Cat# OM165217, RRID: AB_2923048); rabbit anti-Thy1 (1:200, Beyotime Cat# AF1636, RRID: AB_2928953); rabbit anti-GAP43 (1:200, Beyotime Cat# AF0153, RRID: AB_2923049); mouse anti-β-III Tubulin (1:100, Beyotime Cat# AT809, RRID: AB_2893434); mouse anti-CXCR2 (1:50, Santa Cruz Biotechnology Cat# sc-7304, RRID:AB_626893); Cy3, anti-rabbit IgG (1:400, Abbkine Cat# A22220, RRID: AB_2923041); DyLight 488, anti-rabbit IgG (1:200, Abbkine Cat# A23220, RRID:AB_2737289); and DyLight 488, anti-mouse IgG (1:200, Abbkine Cat# A23210, RRID: AB_2923050).

### Flash electroretinogram (ERG)

Flash ERG was performed with the RetiMINER IV system (IRC Medical Equipment, Chongqing, China). For the NMDA injury model, the mice in each group were adapted in darkness overnight 1 day before euthanasia. All handling and experiments were performed under a dim red light to maintain dark adaptation. After anesthetizing with pentobarbital sodium (50 mg/kg) by intraperitoneal injection, atropine sulfate eye gel was used to dilate pupils. Subsequently, thread electrodes were placed across the center of each cornea. A subcutaneous electrode was inserted into the posterior part of the neck (approximately halfway between the ears), and a ground electrode was placed at the end of the mouse’s tail with conductive paste. Bilateral ERG recording was performed from both eyes simultaneously, with each item repeated at least twice. The resulting waveforms were superimposed to confirm the reliability of the recording. Scotopic ERGs were recorded at stimulus intensities levels of 0.001, 0.01, 0.1, 1.0, 3.0, and 10.0 cd. s/m^2^. Oscillatory potentials (OPs) were recorded using the white flashes of 3.0 cd. s/m^2^ scotopic responses via bandpass filtering between 50 and 170 Hz. The positive scotopic threshold responses (pSTRs) were measured from the baseline to the positive peak of the waveform at a flash intensity of 0.001 cd. s/m^2^. After light adaptation for 15 min, the photopic negative responses (PhNRs) were measured from the baseline to the trough of the negative response following the positive b-wave at a flash intensity of 10.0 cd. s/m^2^. Antibiotic eye ointment was applied to reduce the risk of infection after completing the recording procedure.

### Statistical analysis

Each experiment was repeated at least three times. Values are presented as means ± mean standard error (SEM). Differences between means were evaluated with one-way analysis of variance (ANOVA), followed by Bonferroni post hoc tests or nonparametric Kruskal–Wallis test with Dunn’s multiple-comparisons test to compare the results of different groups. Unpaired two-tailed Student’s *t* tests were applied for the comparison between the two groups. All of the statistical analyses were performed using the GraphPad software program and statistical significance was set at *p < 0.05.

## Results

### STS treatment promotes axon outgrowth in vitro

**Fig. 1:**
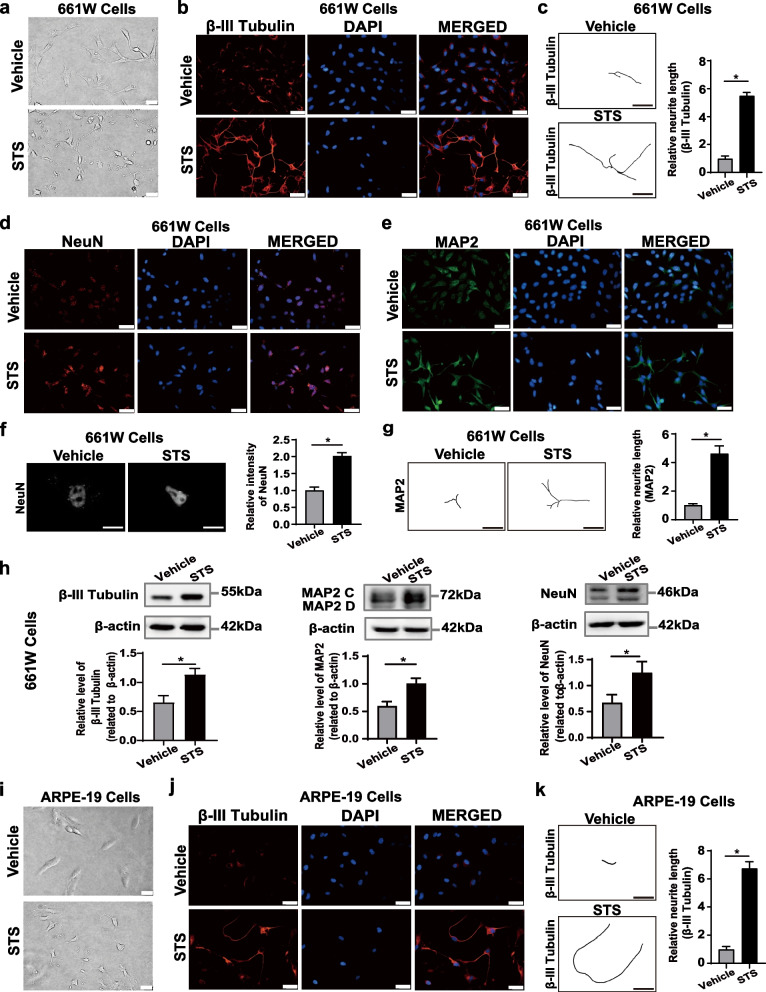
Differentiation of 661W and ARPE-19 cells after STS treatment. **a** Morphological changes of 661W cells treated with 75 nM STS or vehicle (0.05% DMSO in medium) for 6 h. Scale bar, 10 μm. **b** Immunostaining of two groups of 661W cells for neuronal marker β-III Tubulin. Scale bar, 20 μm.** c** Representative neurite tracing images of (**b**). The average lengths of β-III tubulin-positive neurites were compared between the vehicle and STS-treated group. Data are shown as the means ± SEM, *n* = 6 samples per group, *p < 0.05. **d**, **e** Immunostaining of two-group 661W cells for neuronal markers NeuN, and MAP2, respectively. Scale bar, 20 μm. **f**–**g** Representative black-and-white images of (**d**) and neurite tracing images of (**e**), respectively. The average fluorescence intensity of NeuN-positive cells and the average lengths of MAP2-positive neurites were quantified. Results are presented as the means ± SEM (*n* = 6, *p < 0.05). **h** Western blots (cropped blot images) showing the effect of differentiation with STS on the level of β-III tubulin, NeuN, and MAP2 proteins in 661W cells. β-actin was used as an internal control. The results are shown as the means ± SEM (*n* = 5, *p < 0.05). **i** Morphological changes of ARPE-19 cells treated with 75 nM STS or vehicle (0.05% DMSO in medium) for 6 h. Scale bar, 10 μm. **j** β-III tubulin staining images of two-group ARPE-19 cells. Scale bar, 20 μm. **k** Representative neurite tracing images of (**g**) and the quantitative analysis of the average lengths of β-III tubulin-positive neurites. Data are presented as the means ± SEM (*n* = 6, *p < 0.05)

We observed that the treatment with low concentrations of STS (75 nM) for 6 h remarkably promoted axon growth in 661W cells (Fig. 1a), and further immunostaining confirmed that outgrowth of axons was strongly positive for expression of β-III Tubulin (a pan-neuronal maker that exclusively identifies axons [30]), as shown in Fig. 1b, c. Furthermore, the STS treatment-induced 661W cells to differentiate into mature neurons, which showed stopped proliferation and significantly increased expression levels of neuronal markers, such as NeuN, MAP2, and β-III Tubulin (Fig. 1d–h). Intriguingly, we found that the treatment with 75 nM STS for 6 h also induced significant axon-like structure growth in ARPE-19 cells (derived from the human retinal pigment epithelium [28]) and that the regenerated axon-like structures were positively stained with β-III Tubulin as well (Fig. 1j, k). Consistently, the levels of β-III Tubulin in differentiated ARPE-19 cells were also significantly increased (Fig. S1c). In addition, STS treatment also obviously elevated the expression levels of MAP2 but not NeuN in differentiated ARPE-19 cells (Fig. S1a–c). These results suggest that an unknown molecular mechanism to promote axon regeneration commonly exists in the two different types of cells, and STS treatment may activate the underlying signaling to stimulate axon growth.

### The level of the *CXCL2* gene is upregulated in STS-treated cells

**Fig. 2:**
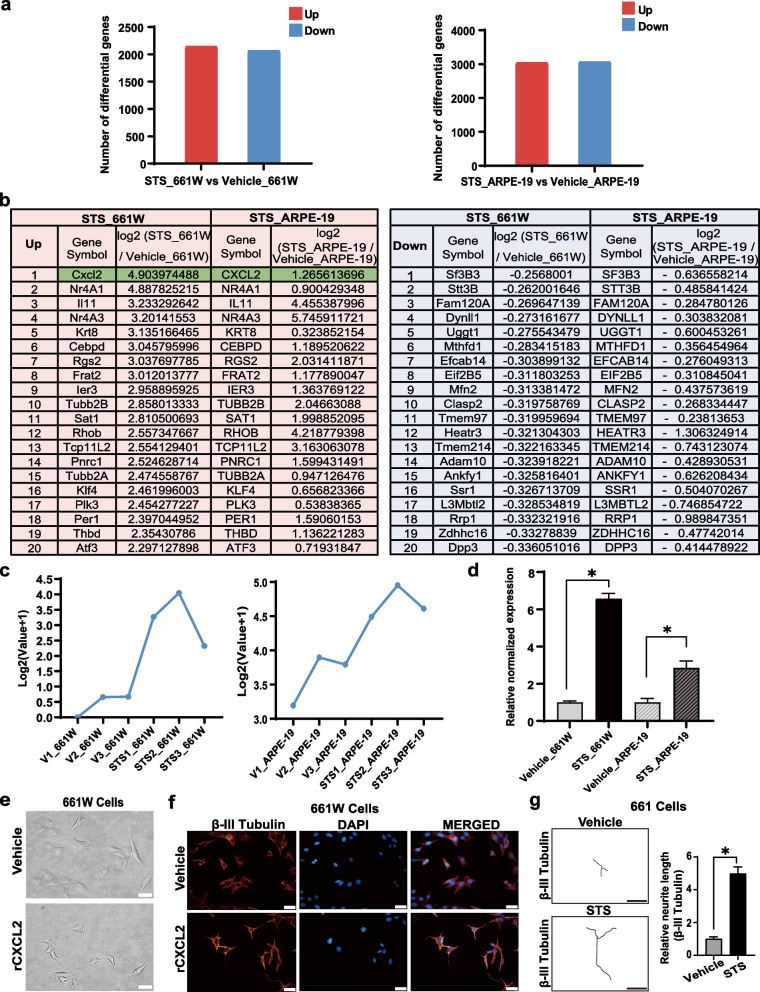
RNA-Seq analysis of 661W and ARPE-19 cells treated with vehicle or STS. **a** The total number of upregulated or downregulated genes in four groups. **b** The top 20 most co-upregulated and co-downregulated genes and their average expression value in two types of cells. **c** The line chart of the *CXCL2* gene expression showed that *CXCL2* gene was upregulated in each biological replicate. **d** The quantitative PCR analyses of CXCL2 expression normalized to *GAPDH* in four groups. The data are presented as the means ± SEM (*n* = 4 or 5, *p < 0.05). **e** Morphological changes of 661W cells treated with 50 nM rCXCL2 or vehicle (medium) for 48 h. Scale bar, 20 μm. **f** β-III tubulin staining images of two-group 661W cells. Scale bar, 20 μm. **g** Representative neurite tracing images of (**f**). The average lengths of β-III tubulin-positive neurites were compared between the two groups. Data are shown as the means ± SEM, *n* = 6 samples per group, *p < 0.05

We analyzed the differentially expressed genes in the two types of STS-treated cells related to each vehicle-treated group after RNA-Seq, and the stacked histogram analysis showed that the RNA-Seq data of each sample in each group were highly reproducible, suggesting that the original RNA-Seq data were of high quality and credibility (Fig. S2). RNA-Seq results showed that there were 2143 upregulated genes and 2068 downregulated genes in STS-treated 661W cells, while there were 3038 upregulated genes and 3064 downregulated genes in STS-treated ARPE-19 cells (Fig. 2a). With further comparative analysis, we found that there was a total of 689 co-upregulated genes and 595 co-downregulated genes in both groups of STS-treated cells (Tables S1 and S2). Among the top co-upregulated genes, the level of the chemokine *CXCL2* gene elevated significantly (log_2_-value: 4.90 in 661W cells vs. 1.27 in ARPE-19 cells), as shown in Fig. 2b, c. We further confirmed the increased expression of the *CXCL2* gene in both groups of differentiated cells with qPCR (Fig. 2d). Next, we verified whether exogenous rCXCL2 may promote axon growth in vitro similar to STS-treatment. As shown in Fig. 2e–g, we found that 48 h after treatment with 50 nM rCXCL2, 661W cells grew obvious axon-like structures which turned positive for β-III Tubulin. Therefore, these results suggest that CXCL2 may play an important role in axon regeneration, and CXCL2 is a potential molecular target for promoting axon growth.

### rCXCL2 promotes axon regeneration and protects RGCs from ONC injury

**Fig. 3:**
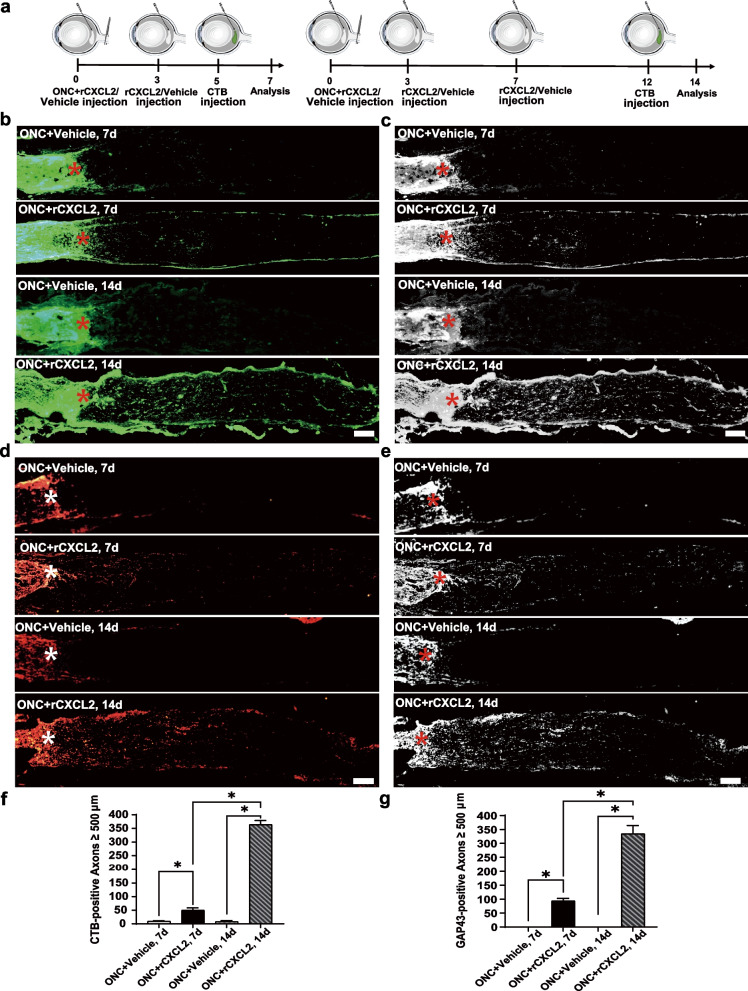
rCXCL2 regenerates RGC axons in ONC models. **a** Timeline of injection process of rCXCL2/vehicle-treated groups within 7 or 14 days. **b** Anterograde CTB tracing of regenerated RGC axons from vehicle (PBS) or rCXCL2-treated eyes 7 and 14 days after ONC. Scale bar, 100 μm, *the crush site. **c** The black-and-white images of (**b**). **d** GAP43.^+^ regenerated axons after ONC with vehicle (PBS) or rCXCL2 injection. Scale bar, 100 μm. **e** The black-and-white images of (**d**). **f** Quantitation of CTB-positive axons 0.5 mm past the injury site. Data are presented as mean ± SEM, *n* = 4 to 5 nerves per group, *p < 0.05. **g** Quantitation of GAP43-positive axons 0.5 mm past the injury site. Results are presented as mean ± SEM, *n* = 4 to 5 nerves per group, *p < 0.05

We next investigated whether rCXCL2 promoted the regeneration of RGC axons in vivo using a murine ONC model. As shown in Fig. 3a–c, 7 days after ONC, anterograde axon tracing with CTB showed that most RGC axons disappeared from the crushed site and CTB staining was nearly invisible. However, after double intravitreal injections of 1.0 μg/μl rCXCL2, we found that the regenerated RGC axons with positive CTB staining were significantly detected in the injured optic nerve. In addition, 14 days after ONC, CTB axon tracing after triple intravitreal injections of rCXCL2 showed a more robust regeneration of RGC axons from the crushed site, in which the longest regenerated axon was able to even reach 1.5 mm behind the eyeball. After a quantitative analysis of regenerated axons in the 7-day and 14-day groups of ONC, we found that an injection of rCXCL2 into the vitreous significantly promoted the regeneration of RGC axons in mice. The number and length of regeneration of RGC axons remarkably increased compared with the vehicle control group, and the RGC axon regeneration in the 14-day group was significantly improved than that in the 7-day group (Fig. 3f). GAP43 specifically expresses in regenerating axons, so it is a commonly used marker for detecting regenerated axons [31]. We further confirmed that the RGC axons regenerated from the crushed site were remarkably positive for GAP43 staining (Fig. 3d, e). Moreover, the quantitative analysis results for the number and length of regenerated axons by GAP43 fluorescent staining were consistent with the results of CTB staining (Fig. 3g).

**Fig. 4:**
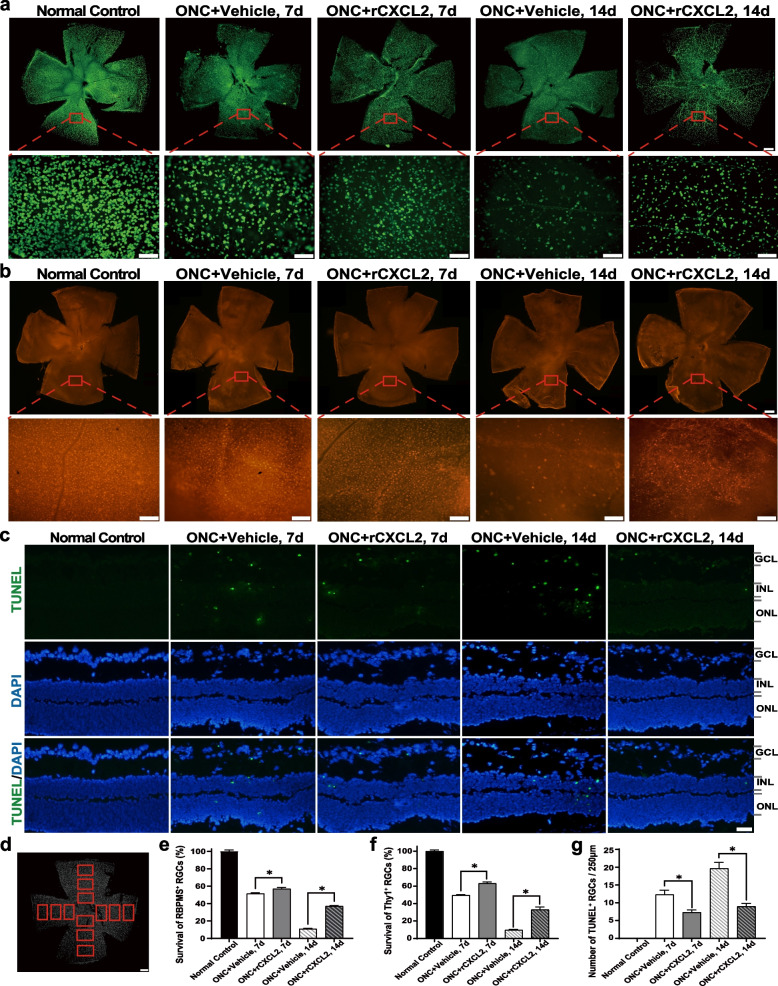
rCXCL2 promotes RGC survival after optic nerve injury. **a**,** b** Immunofluorescence staining images showing RBPMS- or Thy1-positive RGCs in the whole-mount retinas (scale bar: 200 μm) and their representative images of middle regions in the retina (scale bar: 50 μm) 7 and 14 days after ONC with vehicle (PBS) or rCXCL2 injection, respectively. The whole retina was photomerged by several images at low magnification using Adobe Photoshop software. **c** Representative images of TUNEL staining in retinal sections. Scale bar, 20 μm.** d** Scheme of RGC counting; squares represent 12 counting fields (0.35 × 0.45 mm^2^ each). The number of RGCs in each image was counted by three researchers blinded to the experiment and the average number of RGCs was determined. The scale bar is 200 μm. **e**,** f** Percentage of surviving RBPMS^+^ or Thy1^+^ RGCs normalized to a normal control group (100%), respectively. Six to eight biological replicates were used for five groups. Each biological replicate represents the average of 12 different fields in the retina. Data are shown as mean ± SEM, *p < 0.05. **g** The number of TUNEL-positive RGCs in five groups was quantified. Data are presented as mean ± SEM, *n* = 6 samples per group, *p < 0.05

In addition, we also evaluated the protective effect of rCXCL2 on RGC by quantitatively determining the RGC number in the retina after ONC injury. By using whole-mount retinas and the specific marker (RBPMS) to label RGCs, we showed that the total number of RGCs decreased by 51.5% and 10.8% in the 7-day and 14-day Vehicle-treated ONC groups, respectively, while intravitreal injection of rCXCL2 significantly protected RGCs in the ONC injury model, increasing the relative number of RGCs up to 56.9% (7 days) and 40.0% (14 days), as shown in Fig. 4a, d, e. Additionally, with Thy1 labeling RGCs, we found that the total number of RGCs decreased by 42.4% and 9.7% in the 7-day and 14-day vehicle-treated ONC groups, respectively, while intravitreal injection of rCXCL2 significantly elevated the relative number of RGCs up to 47.3% (7 days) and 33.0% (14 days), as shown in Fig. 4b, f. We also performed TUNEL assays in retinal sections to further investigate whether intravitreal injections of rCXCL2 may ameliorate RGC death. We showed that TUNEL-positive RGCs in the 7-day and 14-day rCXCL2-treated ONC groups were considerably fewer than those in the vehicle-treated groups (Fig. 4c, g).

### rCXCL2 protects RGCs and long-distance axon projection from retinal NMDA injury and improves visual function in mice with retinal NMDA damage

**Fig. 5:**
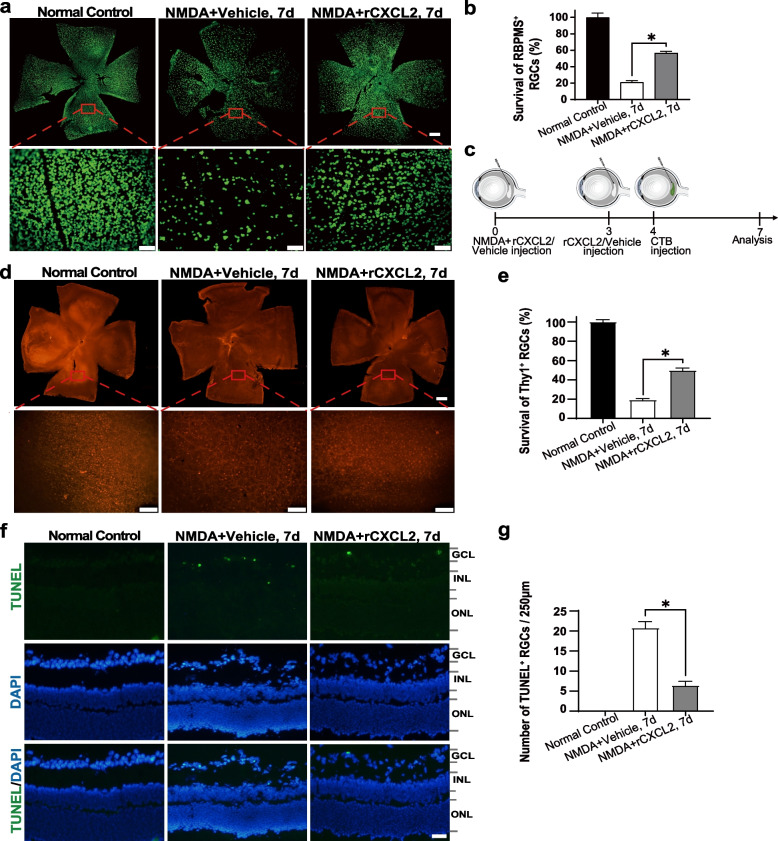
rCXCL2 protects RGCs against retinal NMDA damage. **a** RBPMS staining images of whole-mount retinas (scale bar: 200 μm) and representative images of middle regions in the retina (scale bar: 50 μm) for different groups. The whole retina was photomerged by several images at low magnification with Adobe Photoshop software. **b** Percentage of surviving RBPMS^+^ RGCs normalized to normal control retinas (100%). Five to seven biological replicates were used for three groups. Data are presented as mean ± SEM, *p < 0.05. **c** Timeline of the injection process of an rCXCL2-treated group over seven days. **d** Immunofluorescence staining images showing Thy1^+^ RGCs in the whole-mount retinas (scale bar: 200 μm) and their representative images of middle regions in the retina (Scale bar: 50 μm) 7 days after NMDA injury with vehicle (PBS) or rCXCL2 injection. The whole-mount images of retinas were photomerged from several images at low magnification using Adobe Photoshop software. **e** Percentage of surviving Thy1^+^ RGCs normalized to normal control retinas (100%). Six biological replicates were used for three groups. Data are shown as mean ± SEM, *p < 0.05.** f** Representative images of TUNEL staining in retinal sections. Scale bar, 20 μm. **g** The quantified number of TUNEL.^+^ RGCs in each group. Results are presented as mean ± SEM, *n* = 6 samples per group, *p < 0.05

**Fig. 6:**
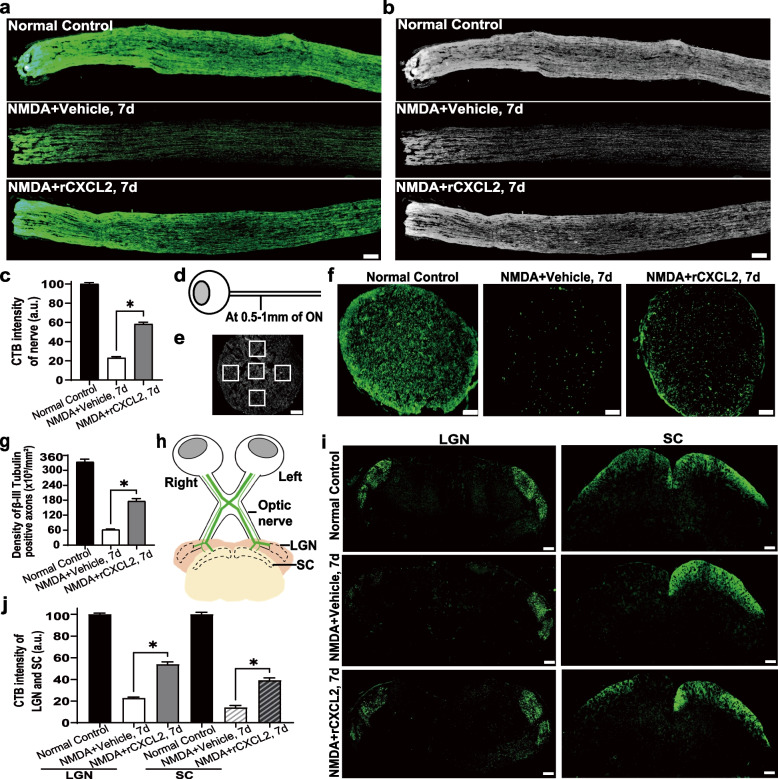
rCXCL2 protects long-distance axon projections targeting the brain in the retinal NMDA injury model. **a** Fluorescence images of anterograde CTB tracing of RGC axons in optic nerves from normal control eyes, and NMDA injection in the vehicle (PBS) or rCXCL2-treated eyes after 7 days. Scale bar, 100 μm. **b** The black-and-white images of **a**. **c** Quantification of CTB intensity in optic nerves normalized to the normal control group (100 a.u.). The results are shown as mean ± SEM, *n* = 4 to 6 nerves per group, *p < 0.05. **d** Schematic view of the location of ON cross-sections. **e** Scheme of the β-III Tubulin-positive axon counting in the cross-section. Squares represent counting fields. Scale bar, 30 μm. **f** Representative images of β-III Tubulin staining in cross-sections at 0.5–1.0 mm of ON. Scale bar, 20 μm. **g** The calculated density of β-III Tubulin–positive axons in cross-sections at 0.5–1.0 mm of ON. The results are presented as mean ± SEM, *n* = 5 to 6 samples per group, *p < 0.05. **h** Schematic illustration of anterograde CTB tracing of the LGN and SC. The right eye of mice was always as the normal control, and the left eye was given different treatment. **i** Fluorescence images of anterograde CTB tracing of RGC axons projecting to the LGN and SC from normal control eyes, and 7 days after NMDA injection in the vehicle (PBS) or rCXCL2-treated eyes. Scale bar, 200 μm. **j** Quantification of CTB intensity in the LGN and SC normalized to the normal control group (100 a.u.) after normalizing the right hemisphere to the left hemisphere. Data are presented as mean ± SEM, *n* = 4 brains per group, *p < 0.05

Exogenous NMDA can induce RGC excitotoxic death through NMDA receptors and lead to Wallerian-like degeneration of axons in the optic nerve and loss of target innervation in the brain [32]. We verified whether exogenous rCXCL2 protected RGCs from excitotoxic damage with the retinal NMDA injury model. As shown in Fig. 5a, b, after intravitreal injection of 20 mM NMDA for 7 days, the survival rate of RBPMS-positive RGCs was only approximately 20.9%, while intravitreal injection of rCXCL2 exhibited a significant protective effect and elevated the RGC survival rate to 56.5%. In RGCs labeled with Thy1, the total number of RGCs decreased by 16.4% in the 7-day vehicle-treated NMDA groups, while rCXCL2 treatment significantly increased the relative number of RGCs up to 42.4% (Fig. 5d, e). We also found that the TUNEL-positive cells in the GCL of NMDA-injured retinas were markedly reduced upon rCXCL2 treatment (Fig. 5f, g). In addition, exogenous rCXCL2 also protects RGC axons and long-distance projections in the brain from NMDA injury. CTB axon tracing, as shown in Fig. 6a–b, indicated that the number of RGC axons decreased significantly after NMDA treatment, and the fluorescence density of CTB-labeled optic nerve decreased by 23.1% in the 7-day vehicle-treated NMDA-damaged group. However, intravitreal injection of rCXCL2 significantly preserved RGC axons, and increased the fluorescence intensity of CTB-labeled optic nerve to 55.9% (Fig. 6c). Additionally, we further assessed the survival RGC axons by calculating the number of axons positively stained with β-III Tubulin at the cross-sections of the optic nerve at 0.5–1.0 mm behind the eyeball in each group after NMDA injury (Fig. 6d–f). As shown in Fig. 6g, the density of β-III Tubulin-positive axons decreased to 62 × 10^3^/mm^2^ 1 week after NMDA injury, while the axon density was significantly maintained at 176 × 10^3^/mm^2^ after rCXCL2 treatment.

RGC axons predominantly project to the contralateral thalamus in mice, and the lateral geniculate nucleus (LGN) and superior colliculus (SC) are the two main projection targets of RGC axons in the brain [6] (Fig. 6h). We observed that there was severe loss of RGC axonal projections on the 7th day after NMDA injection, with only 22.6% and 14.0% of CTB intensity left in the contralateral LGN and SC, respectively, while rCXCL2 treatment nearly doubled the RGC axonal projections to the contralateral LGN (53.7% of CTB intensity) and SC (39.0%) (Fig. 6i, j).

**Fig. 7:**
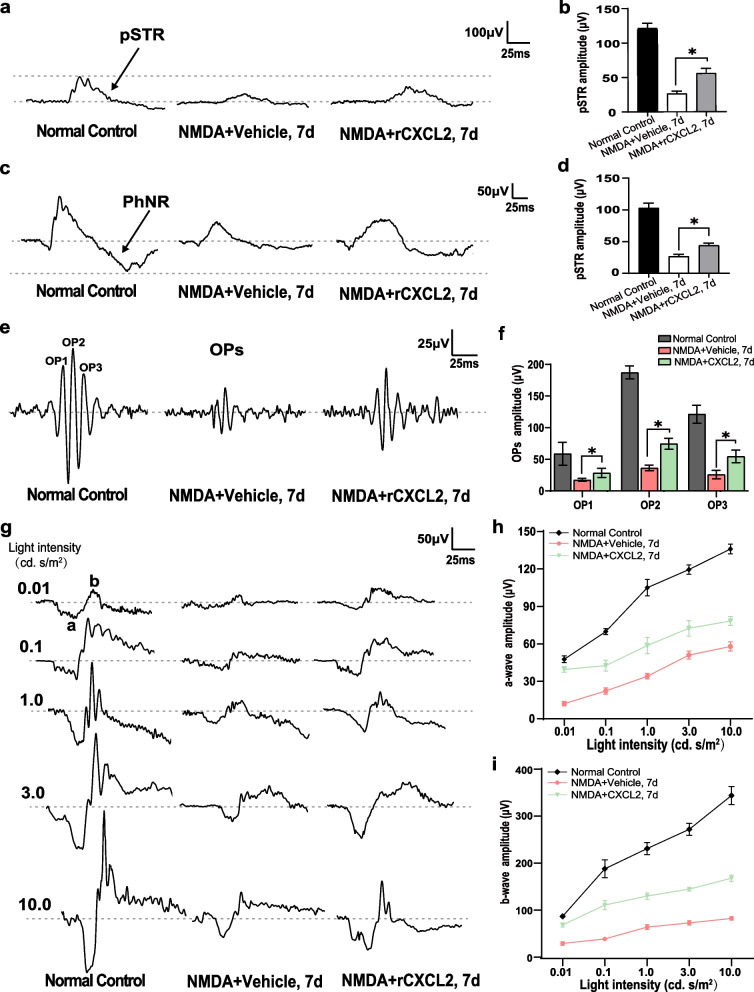
rCXCL2 treatment improves visual function in mice with retinal NMDA injury. **a** Representative pSTR waveforms from normal control eyes, as well as NMDA injection in the vehicle (PBS) or rCXCL2-treated eyes, after 7 days as recorded by flash ERG responses. The pSTR was elicited with a very dim flash stimuli at an intensity of 0.001 cd. s/m^2^ and measured from the baseline to the positive peak of the waveform. **b** Averaged data of pSTR amplitudes from each group. The results are shown as mean ± SEM, *n* = 8 eyes per group, *p < 0.05. **c** Representative PhNR waveforms from each group. The PhNR was elicited with strong light stimuli at the flash intensity of 10.0 cd. s/m^2^ and measured from the baseline to the trough of the negative response following the b wave. **d** Averaged data of PhNR amplitudes from each group. Data are presented as mean ± SEM, *n* = 5 eyes per group, *p < 0.05. **e** Representative OP waveforms from each group recorded with the white flashes of 3.0 cd. s/m^2^ scotopic responses via bandpass filtering between 50 and 170 Hz. **f** The averaged data of OP (OP1, OP2, and OP3) amplitudes from each group were quantified. The results are shown as mean ± SEM, *n* = 9 eyes per group, *p < 0.05. **g** Representative scotopic series (a- and b- waves) in response to the increasing flash intensity from each group. The flash intensities used to elicit the responses are presented to the left. **h**, **i** Line chart shows the amplitudes of a- and b- waves in scotopic ERG responses, respectively

We also investigated whether rCXCL2 affected the visual functions of mice subjected to NMDA damage using flash ERG recordings. In flash ERG responses, the pSTR and PhNR are generated in the RGCs, the OPs are generated in the amacrine cells, and the a/b waves are generated from the photoreceptor cells to cells located in the inner nuclei layer (INL) [33, 34]. We found that the amplitudes of pSTR and PhNR were decreased by 26.4 μV and 26.6 μV in the 7-day vehicle-treated NMDA-damaged group, respectively, indicating the functional damage to RGCs in mice with retinal NMDA injury, while intravitreal injection of rCXCL2 significantly improved the visual function of RGCs in the NMDA injury model, increasing the pSTR and PhNR amplitudes up to 56.0 μV and 44.0 μV, as shown in Fig. 7a–d. Additionally, the NMDA-mediated reduction in scotopic OPs, *a*-wave and *b*-wave were also significantly restored by rCXCL2 treatment (Fig. 7g–i).

Thus, our results demonstrate that rCXCL2 not only protects RGC somas from retinal NMDA injury but also preserves the integrity of RGC axons in the optic nerve and their distal target innervations in the brain. In addition, rCXCL2 treatment improves the visual function of mice with retinal NMDA damage.

### rCXCL2 activates different signaling pathways in ONC/NMDA-damaged retinas

**Fig. 8:**
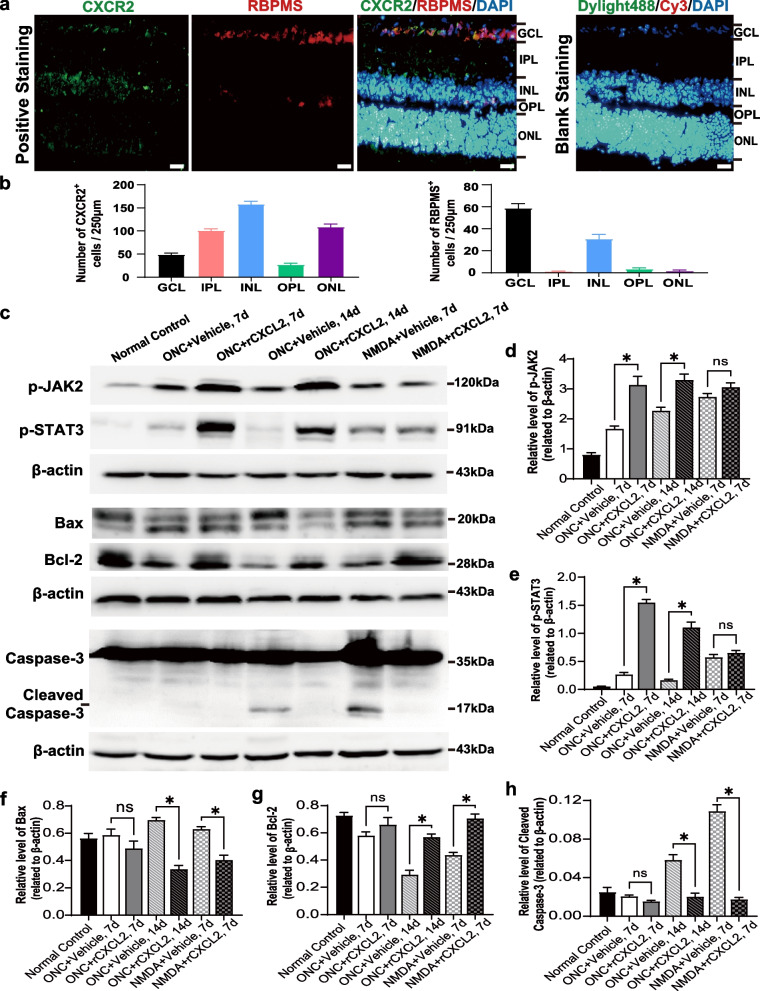
rCXCL2 binds with CXCR2 to activate different signaling pathways in ONC/NMDA-damaged retinas. **a** Immunofluorescence images of retinal sections to visualize the specific CXCL2 receptor, CXCR2 (green), RGCs (anti-RBPMS) (red), and cellular nucleus (DAPI) (blue). Scale bar, 20 μm. **b** Quantification of the number of CXCR2^+^ or RBPMS^+^ cells in each layer of retinal cells. Data are presented as mean ± SEM. **c** Western blots (cropped blot images) showing the protein expression of p-JAK2, p-STAT3, Bax, Bcl-2, caspase-3, and β-actin. **d**–**h** Western blot analysis of **b**, β-actin was used as an internal control. The results are shown as the means ± SEM (for **c**–**f**, *n* = 4; for **g**, *n* = 3, *p < 0.05, ns: not statistically significant). GCL, ganglion cell layer; IPL, inner plexiform layer; INL, inner nuclear layer; OPL, outer plexiform layer; ONL, outer nuclear layer

CXCR2, as a seven-transmembrane G-protein, is the only specific receptor of CXCL2 [35]. We determined the localization of CXCR2 in the mouse retina through immunostaining and revealed that CXCR2 was predominantly expressed in the ganglion cell layer (GCL) and INL. Furthermore, we also confirmed that CXCR2 was predominantly present on RGC soma in GCL, which was verified with the double staining of RBPMS (Fig. 8a). The number of CXCR2- or RBPMS-positive cells in each retinal cell layer was quantified (Fig. 8b).

The Janus kinase (JAK2)/signal transducer and activator of transcription (STAT3) signaling pathway is one of the major pathways mediated by CXCR2 to regulate cell survival and plays a crucial role in the regeneration of RGC axons [36–40]. We therefore evaluated whether exogenous rCXCL-activated JAK2/STAT3 signal. As shown in Fig. 8c–e, the treatment of rCXCL2 caused activation of JAK2/STAT3 in mouse retina determined on the 7th and 14th day after ONC injury, showing upregulation of the p-JAK2/p-STAT3 levels. However, there was no statistically significant difference observed in the p-JAK2/p-STAT3 levels in rCXCL2-treated NMDA-injured retinas as compared with the vehicle group (Fig. 8c–e).

The Caspase-3 dependent pathway is reported as a predominant pathway of RGC death caused by ONC/NMDA injury [41–43], so we further comparatively investigated the levels of pro-apoptotic factors Caspase-3 and Bax, and the anti-apoptotic factor Bcl-2 between the two injured groups. As shown in Fig. 8c, f–h, ONC injury failed to induce the activation of Caspase-dependent pathway on the 7th day, because Cleaved Caspase-3 was barely detectable, yet on the 14th day after ONC injury, delayed activation of the Caspase pathway could be detected, showing upregulated Cleaved Caspase-3. However, the upregulated Cleaved Caspase-3 was remarkably detectable on the 7th day after NMDA injury, indicating that NMDA-injury makes more significant strikes on RGC somas, with more rapid death of RGCs than that induced by ONC. Importantly, rCXCL2 injections significantly reduced the upregulation of pro-apoptotic factors Bax and Cleaved Caspase-3 caused by ONC injury (on the 14th day) or NMDA damage (on the 7th day). The double intravitreal injections of rCXCL2 also caused slight upregulation of Bcl-2 in mice retina on the 7th day after ONC injury, but significantly increased Bcl-2 level on the 7th day after NMDA damage, and triple intravitreal injections of rCXCL2 led to significant upregulation of Bcl-2 on the 14th day post-ONC injury.

Thus, these results indicate that JAK2/STAT3 signaling pathway might involve in rCXCL2 stimulated-axon regeneration in the ONC-damaged model and rCXCL2 might protect RGCs by regulating the Caspase-3 dependent pathway, but further investigations need to be performed to make the conclusion.

## Discussion

**Fig. 9:**
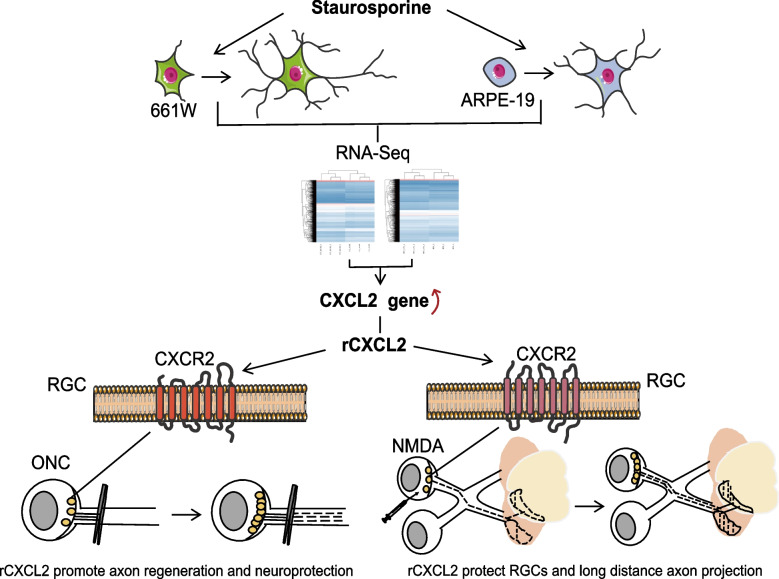
Schematic illustration depicting that CXCL2 contributes to axon regeneration and neuroprotection in RGC injury

As a broad-spectrum PKC inhibitor, STS isolated from *Streptomyces staurosporesa* is able to promote the outgrowth of neurites in a variety of cells such as HN33 hippocampal cells, PC12 chromocytoma cells, and SH-SY5Y neuroblastoma cells [25–27]. Recently, Kase et al. showed that another compound produced by *Streptomyces staurosporesa*, RK-682, promoted microtubule polymerization, and axon elongation in human neurons by enhancing p38 MAPK/CDC25B signaling [44]. Although some studies have shown that STS may induce dopaminergic axon growth by activating the mTOR signaling pathway via inhibiting AMPK [45], the detailed molecular mechanism still remains to be further investigated. The 661W cells derived from mouse retinal tumors positively express cone marker Opn1mw; RGC markers such as RBPMS, BRN3b (Pou4f2), BRN3c (Pou4f3), Thy1, and γ-synuclein (Sncg); and some other neuronal markers (β-III Tubulin, NeuN, and MAP2) [28]; however, ARPE-19 cells derived from the human pigment epithelium, expressing the specific pigment epithelium CRALBP and RPE65, shows distinct differences in origin and growth features from 661W cells [29]. In this study, we found that low concentrations of STS can robustly promote the axon growth of 661W cells and induce the cells to differentiate into mature neuronal cells, resulting in a significant increase in the expression of the mature neuron markers NeuN, MAP2, and β-III Tubulin. More intriguingly, we also found that ARPE-19 cells exhibited similar axon outgrowth as 661W cells after STS treatment. Thus, we speculate that a potential molecular mechanism commonly exists to promote axon regeneration in the two types of cells, and STS treatment may initiate the signals to promote axon outgrowth in these cells despite them having different origins. Based on this strategy, we performed a whole-transcriptome sequencing for 661W and ARPE-19 cells after STS induction, and comparatively analyzed the change in gene expression (Fig. 9). The results of mRNA-seq showed that there was a total of 595 co-downregulated genes and 689 co-upregulated genes in these cells after STS induction. We next ranked these co-regulated genes based on the significance of their expression quantity, and we excluded most of genes after further investigation into functions and associated pathways, such as *TUBB2A*, *TUBB2B* (encoding the structural constituents of the cytoskeleton), *CEBPD* (a versatile modulator encoding DNA-binding transcription in the nucleus), *SF3B3* (encoding the chromatin transcription and DNA repair), and *MTHFD1* (encoding a protein that catalyzes the interconversion of tetrahydrofolate) [46–49]. In the process of gene screening, we paid high attention to chemokine family members, due to their crucial role in regulating axon regeneration [10, 22, 50], and finally, we targeted the chemokine *CXCL2* because of its top significance of the increased expression level among the co-upregulated genes. More importantly, with further verification we found that exogenous rCXCL2 significantly induced axon outgrowth in cultured 661W cells in vitro with a similar effect as STS treatment. These results pushed us to further investigate the role of rCXCL2 in promoting axon regeneration with RGC-injured animal models in vivo.

There are four subfamilies in all identified chemokines, CXC, CC, CX3C, and XC, and CXCL2 is one of the 17 members in the CXC subfamily of chemokines [51]. Chemokine proteins are generally synthesized in cells as peptide precursors and secreted and released from cells guided by signal peptides. Most chemokines exert their biological effects through interactions with GPCR (G-protein-coupled transmembrane receptors, called chemokine receptors) [20]. Studies have shown that members of the CXC subfamily of chemokines play an important role in inflammation-induced axon regeneration [10]. CXCL12(SDF-1)/CXCR4 signaling is crucial for guiding RGC axons to exit the retina from the optic stalk, and knockdown of the ligand or a genetic mutation of CXCR4 led RGC axons to deviate from their original paths in zebrafish embryos [21]. In the rat cerebellar axons, CXCL12 also served as a guidance cue for axon pathfinding [23]. In addition, some studies have shown that the administration of exogenous chemokines also can promote RGC axon regrowth and protect RGCs from injuries. For instance, the intravitreal application of CXCL12 increased the activity of mTOR in RGCs and moderately stimulated axon regeneration upon ONC injury [52]. In cultured rat cortical neurons, recombinant CXCL5 improved the survival of RGCs [53], and CXCL5 secreted from adipose-derived stem cells promoted the growth of axons in rat pelvic ganglion cells by activating the JAK/STAT pathway [54]. Recently, it has also been reported that CXCL5 significantly promotes RGC survival and axon regeneration in an ONC injury model and if combined with experimental lens injury, the protective effect of CXCL5 on RGCs can be further enhanced [22]. In this study, we found that intravitreal injection of exogenous rCXCL2 significantly promoted axon regeneration and attenuated the death of RGCs in ONC-injured mice, whereas it exhibited a different role in the retinal NMDA-damaged model (Fig. 9). The intravitreal injection of rCXCL2 improved RGC survival and maintained the long-distance projection of RGC axons targeting the thalamus in the NMDA-induced retinal excitotoxicity, but failed to induce significant axon regeneration. With further investigation of the signal difference, we disclosed that the intravitreal injection of exogenous CXCL2 led to a significant activation of p-JAK2/p-STAT3 pathway in the ONC-injured model, but not in NMDA-induced retina excitotoxicity, suggesting that JAK2/STAT3 might be an important signaling pathway for CXCL2 to promote the axon regeneration of RGCs. However, although our study demonstrated that RGCs located in the inner layer of mice retina indeed expressed CXCL2 receptor positively, we still cannot exclude the possibility that the administration of exogenous rCXCL2 may act on amacrine cells or Müller cells to indirectly trigger the activation of signals inducing RGC axon regeneration. Thus, the detailed molecular mechanisms of CXCL2-promoted axon regeneration remain to be further elucidated in future studies.

In this study, we applied two different RGC injury models for a comparative study. Both models share a common pathological feature, i.e., the death of RGCs and the loss of axons. However, the difference between the two models is that the axonal injury of RGCs occurs first in the ONC model and then causes the retrograde RGC death and the anterograde loss of axon from the crush-damaged site; however, NMDA-induced excitotoxic insult may directly cause the damage of RGC somas, resulting in the anterograde degeneration of axons [55]. The death mechanism of RGCs induced by ONC is relatively complex. For example, the failure of axonal transportation, the deprivation of neurotrophic factors, the misbehavior of glial cells, and the loss of synaptic connectivity occurs after axon injury, leading to the eventual activation of death signals in RGCs [10, 56, 57]. However, exogenous NMDA predominantly induces neuronal glutamate excitotoxicity mediated through its receptors to cause the death of RGCs. Once the NMDA receptor is activated, it initiates a massive influx of Ca^2+^, resulting in mitochondrial dysfunction, DNA damage, and peroxide generation [58, 59], which eventually provoke caspase-mediated apoptotic cell death [60]. In this study, we found that the levels of Cleaved Caspase-3 and Bax hardly elevated on the 7th day after ONC injury, yet increased on the 14th day after ONC injury and on the 7th day after NMDA damage. rCXCL2 injections attenuated the upregulation of Cleaved Caspase-3 and Bax and induced upregulation of Bcl-2 on the 14th day after ONC and 7th day after NMDA damage. This suggested that NMDA damage made a more severe strike on RGC somas compared to the ONC injury, which induced the faster activation of caspase-dependent apoptotic signal and led to rapid death of RGCs, while rCXCL2 injections might attenuate the caspase-dependent RGC death caused by both injuries. In addition, the intravitreal injections of rCXCL2 activated the JAK2/STAT3 pathway only in the ONC injury model but failed in the NMDA injury model. The differences in these results may attribute to the different pathological mechanisms in the two injury models. In the ONC model, the damage of RGCs is secondary to the crush of RGC axons, which may provide a suitable time window for axon regeneration, and the intracellular microenvironment is relatively stable for rCXCL2 to induce axon regrowth. However, in the NMDA-injured retinas, the intracellular microenvironment of RGCs is remarkably changed because of Ca^2+^ influx, peroxide accumulation, and organelle dysfunction, which are caused by the continuous existence of NMDA, eventually resulting in the failure of axonal transport and persistent inhibition of axon regeneration. This indicates that regenerating the axon requires a healthy RGC soma to sustain axonal transport for axon elongation and wiring. Therefore, the different insults may lead to different consequences in promoting axon regeneration of RGCs though they may share similar pathological features such as the death of RGCs and the loss of axons, which should be highlighted in the investigations of axon regeneration.

## Conclusions

In this study, we demonstrated that the target factor rCXCL2 may robustly promote axon regeneration of RGC in the ONC-damaged mice model and significantly maintain long-distance axon projections in NMDA-induced retinal excitotoxicity. In addition, the exogenous rCXCL2 significantly improved the survival of RGCs against two different types of injury. Overall, our results indicate that CXCL2 as an inflammatory factor plays a key role in regulating the axon regeneration and neuroprotection of RGCs. Importantly, these insights will facilitate the development of promising neuroprotective and axon regenerative strategies for the clinical treatment of RGC degenerative diseases.

## Supplementary Information

**Additional file 1:** **Figure S1.** Immunostaining and western blots showing the expression of neural markers in ARPE-19 cells after STS treatment. a, b. Immunostaining for neuronal markers NeuN and MAP2 in ARPE-19 cells treated with 75 nM STS or vehiclefor 6 h. Scale bar, 10 μm. c Western blotsshowing the effect of differentiation with STS on the level of βIII tubulin, NeuN and MAP2 proteins in ARPE-19 cells. β-actin was used as an internal control. The results are shown as the means ± SEM.

**Additional file 2:** **Figure S2.** Stacked histogram analysis of transcriptome sequencing data showed that the RNA-Seq data of each sample in each group were highly reproducible. Transcripts Per Million<= 1 indicates a gene with a very low expression level, TPM between 1 and 10 is a gene with a lower expression level, TPM Genes with> = 10 are highly and moderately expressed.

**Additional file 3:** **Table S1.** There was a total of 689 co-upregulated genes in both groups of STS-treated cells.

**Additional file 4:** **Table S2.** There was a total of 595 co-downregulated genes in both groups of STS-treated cells.

**Additional file 5.** Original blot images.

## Data Availability

The datasets analyzed during the current study are available from the corresponding author on reasonable request.

## Abbreviations

AAV: Adeno-associated virus
BSA: Bovine serum albumin
CTB: Cholera Toxin Subunit B
DAPI: 4′,6-Diamino-2-phenylindole
DMEM: Dulbecco’s modified Eagle’s medium
DMEM/F-12: DMEM/nutrient mixture F-12
DNB: DNA nanoball
ERG: Electroretinogram
FBS: Fetal bovine serum
GCL: Ganglion cell layer
GPCR: G-protein-coupled transmembrane receptors
INL: Inner nuclei layer
JAK: Janus kinase
LGN: Lateral geniculate nucleus
mTOR: Mammalian target of rapamycin
NMDA: *N*-Methyl-d-aspartate
ON: Optic nerve
ONC: Optic nerve crush
OP: Oscillatory potential
PBS: Phosphate buffer saline
PCR: Polymerase chain reaction
PFA: Paraformaldehyde
PhNR: Photopic negative responses
PI3K: Phosphatidylinositol 3-kinase
PKB/AKT: Protein kinase B
PKC: Protein kinase C
pSTR: Positive scotopic threshold response
rCXCL2: Recombinant CXCL2
RGCs: Retinal ganglion cells
RT: Room temperature
SC: Superior colliculus
STAT: Signal transducer and activator of transcription
STS: Staurosporine
TUNEL: Terminal-deoxynucleotidyl-transferase (TdT)-mediated dUTP-biotin nick end labeling
