# Supplementary material for: Promotion of axon regeneration and protection on injured retinal ganglion cells by rCXCL2
Source: Inflamm Regen. 2023 Jun 20;43:31. doi: 10.1186/s41232-023-00283-5 (PMC10280836; doi:10.1186/s41232-023-00283-5)
Supplement: Supplementary file 1 — Additional file 1: Figure S1. Immunostaining and western blots showing the expression of neural markers in ARPE-19 cells after STS treatment. a, b. Immunostaining for neuronal markers NeuN and MAP2 in ARPE-19 cells treated with 75 nM STS or vehiclefor 6 h. Scale bar, 10 μm. c Western blotsshowing the effect of differentiation with STS on the level of βIII tubulin, NeuN and MAP2 proteins in ARPE-19 cells. β-actin was used as an internal control. The results are shown as the means ± SEM. [file 41232_2023_283_MOESM1_ESM.pdf]

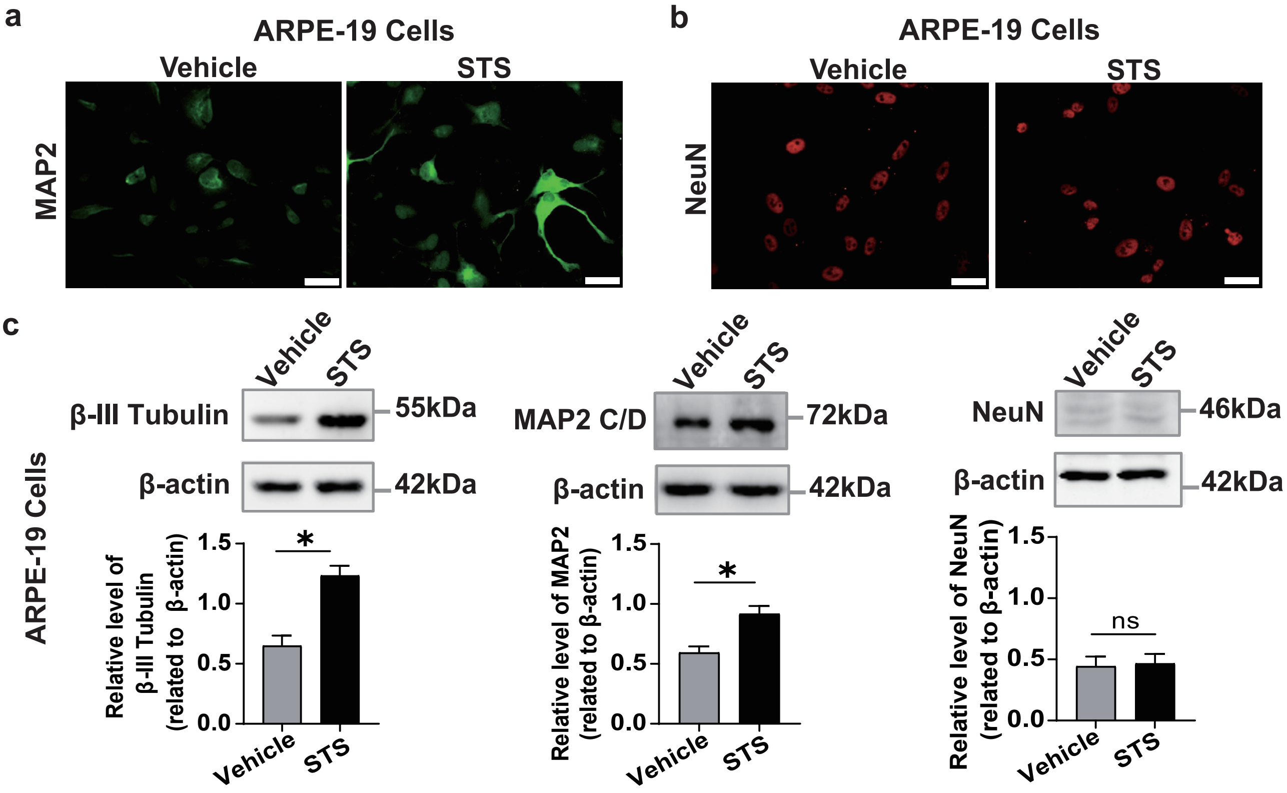

Figure S1. Immunostaining and western blots showing the expression of neural markers in ARPE-19 cells after STS treatment. **a, b.** Immunostaining for neuronal markers NeuN and MAP2 in ARPE-19 cells treated with 75 nM STS or vehicle (0.05% DMSO in medium) for 6 h. Scale bar, 10 µm. **c** Western blots (cropped blot images) showing the effect of differentiation with STS on the level of β-III tubulin, NeuN and MAP2 proteins in ARPE-19 cells. β-actin was used as an internal control. The results are shown as the means ± SEM (n = 5, *: P < 0.05, ns: not statistically significant).
