# Supplementary material for: Promotion of axon regeneration and protection on injured retinal ganglion cells by rCXCL2
Source: Inflamm Regen. 2023 Jun 20;43:31. doi: 10.1186/s41232-023-00283-5 (PMC10280836; doi:10.1186/s41232-023-00283-5)
Supplement: Supplementary file 2 — Additional file 2: Figure S2. Stacked histogram analysis of transcriptome sequencing data showed that the RNA-Seq data of each sample in each group were highly reproducible. Transcripts Per Million<= 1 indicates a gene with a very low expression level, TPM between 1 and 10 is a gene with a lower expression level, TPM Genes with> = 10 are highly and moderately expressed. [file 41232_2023_283_MOESM2_ESM.pdf]

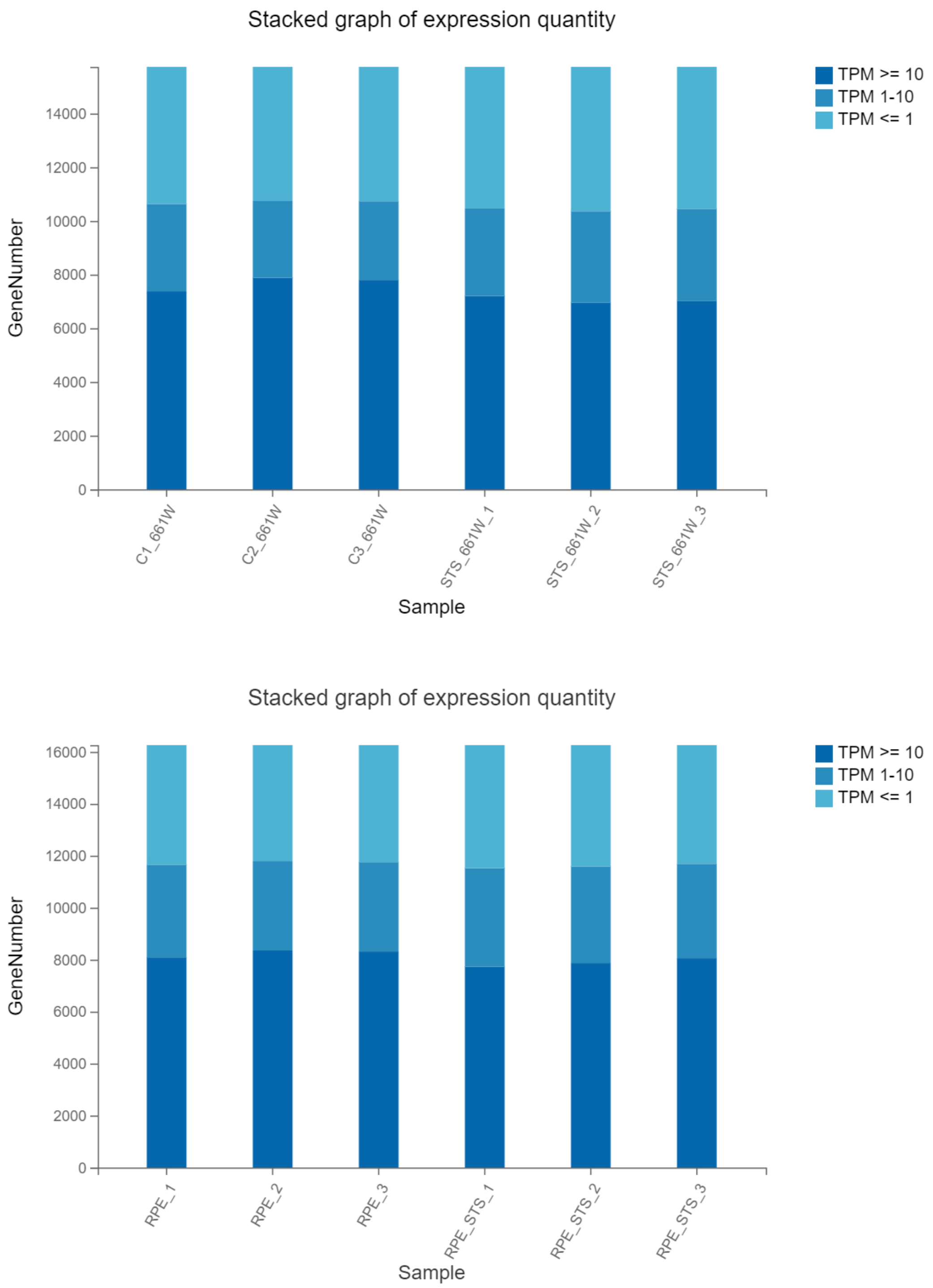

Figure S2. Stacked histogram analysis of transcriptome sequencing data showed that the RNA-Seq data of each sample in each group were highly reproducible. Transcripts Per Million (TPM) <= 1 indicates a gene with a very low expression level, TPM between 1 and 10 is a gene with a lower expression level, TPM Genes with> = 10 are highly and moderately expressed.
