# Supplementary material for: Promotion of axon regeneration and protection on injured retinal ganglion cells by rCXCL2
Source: Inflamm Regen. 2023 Jun 20;43:31. doi: 10.1186/s41232-023-00283-5 (PMC10280836; doi:10.1186/s41232-023-00283-5)
Supplement: Supplementary file 3 — Additional file 3: Table S1. There was a total of 689 co-upregulated genes in both groups of STS-treated cells. [file 41232_2023_283_MOESM3_ESM.pdf]

Table S1. There was a total of 689 co-upregulated genes in both groups of STS-treated cells.

No.	Gene ID	Symbol	Type	log2 (STS_661W / Vehicle_661W)	Qvalue (STS_661W / Vehicle_661W)	Gene ID	Symbol	Type	log2 (STS_ARPE19 / Vehicle_ARPE19)	Qvalue (STS_ARPE19 / Vehicle_ARPE19)
1	11350	Abl1	mRNA	0.699	0.00178	25	ABL1	mRNA	0.21515	0.035309
2	56215	Acin1	mRNA	0.5639	3.78E-06	22985	ACIN1	mRNA	0.24911	0.00839
3	171210	Acot2	mRNA	1.0509	0.002303	10965	ACOT2	mRNA	0.43134	0.000824
4	56360	Acot9	mRNA	0.8018	3.71E-06	23597	ACOT9	mRNA	0.39442	5.05E-05
5	54130	Actr1A	mRNA	0.4778	4.89E-05	10121	ACTR1A	mRNA	0.24922	0.003387
6	27360	Add3	mRNA	0.4595	0.04127	120	ADD3	mRNA	0.35748	0.002221
7	102216272	Ak6	mRNA	0.697	2.73E-05	102157402	AK6	mRNA	0.39483	0.015397
8	68050	Akirin1	mRNA	0.7496	1.27E-07	79647	AKIRIN1	mRNA	0.48847	3.92E-08
9	433693	Akirin2	mRNA	0.5217	9.41E-05	55122	AKIRIN2	mRNA	0.96596	1.95E-17
10	52717	Anapc16	mRNA	0.431	0.00033	119504	ANAPC16	mRNA	0.51812	2.17E-08
11	11727	Ang	mRNA	1.4821	0.000979	283	ANG	mRNA	0.64186	0.020776
12	67628	Anp32B	mRNA	0.2761	0.016492	10541	ANP32B	mRNA	0.28346	0.000679
13	11744	Anxa11	mRNA	0.4516	0.020373	311	ANXA11	mRNA	0.39819	2.36E-06
14	11747	Anxa5	mRNA	0.4174	0.00229	308	ANXA5	mRNA	0.22877	0.01448
15	108012	Ap1S2	mRNA	0.429	0.005482	8905	AP1S2	mRNA	0.39466	2.74E-05
16	72103	Aplf	mRNA	0.7492	9.23E-06	200558	APLF	mRNA	1.3408	1.33E-15
17	66884	Appbp2	mRNA	0.4449	0.006872	10513	APPBP2	mRNA	0.4805	9.36E-07
18	11845	Arf6	mRNA	0.3448	0.006045	382	ARF6	mRNA	0.60711	1.34E-12
19	16800	Arhgef2	mRNA	0.7276	0.044437	9181	ARHGEF2	mRNA	2.05827	2.12E-95
20	238247	Arid4A	mRNA	0.5822	0.001063	5926	ARID4A	mRNA	1.36531	7.72E-21
21	94246	Arid4B	mRNA	0.8916	5.34E-11	51742	ARID4B	mRNA	0.28187	0.012256
22	23806	Arih1	mRNA	0.3665	0.001293	25820	ARIH1	mRNA	0.36823	5.33E-05
23	68724	Arl8A	mRNA	0.6521	6.58E-05	127829	ARL8A	mRNA	0.47426	5.24E-05
24	233912	Armc5	mRNA	0.506	0.047094	79798	ARMC5	mRNA	0.43116	0.006876
25	71703	Armcx3	mRNA	0.4431	0.048434	51566	ARMCX3	mRNA	0.28957	0.013649
26	11865	Arntl	mRNA	0.8619	0.000297	406	ARNTL	mRNA	1.53762	3.27E-36
27	56378	Arpc3	mRNA	0.4626	0.011516	10094	ARPC3	mRNA	0.40175	2.70E-06
28	105171	Arrdc3	mRNA	0.4747	0.005313	57561	ARRDC3	mRNA	2.86142	1.13E-121
29	72323	Asb6	mRNA	0.4043	0.010512	140459	ASB6	mRNA	0.29128	0.013005
30	66403	Asf1A	mRNA	0.4638	0.009608	25842	ASF1A	mRNA	0.73701	1.06E-09
31	11909	Atf2	mRNA	0.5815	5.35E-06	1386	ATF2	mRNA	0.54666	3.43E-09
32	11910	Atf3	mRNA	2.2971	0.012174	467	ATF3	mRNA	0.71932	1.45E-06
33	11911	Atf4	mRNA	0.789	0.014198	468	ATF4	mRNA	1.38043	8.46E-10
34	67526	Atg12	mRNA	0.5146	0.008823	9140	ATG12	mRNA	0.41662	2.21E-05
35	51897	Atg13	mRNA	0.6333	0.000758	9776	ATG13	mRNA	0.62932	1.87E-14
36	100504663	Atg14	mRNA	0.788	5.76E-05	22863	ATG14	mRNA	0.62055	6.11E-08
37	329015	Atg2A	mRNA	0.9839	1.53E-06	23130	ATG2A	mRNA	0.59447	4.36E-08
38	13498	Atn1	mRNA	0.732	0.000391	1822	ATN1	mRNA	0.50159	1.34E-06
39	11973	Atp6V1E1	mRNA	0.4979	0.001022	529	ATP6V1E1	mRNA	0.50797	1.92E-08
40	108664	Atp6V1H	mRNA	0.3954	0.040276	51606	ATP6V1H	mRNA	0.32482	0.0017
41	233871	Atxn2L	mRNA	0.98	1.72E-05	11273	ATXN2L	mRNA	0.39315	5.46E-06
42	78937	Avl9	mRNA	1.0099	2.32E-10	23080	AVL9	mRNA	0.61541	2.89E-09
43	69534	Avpi1	mRNA	0.4319	0.007135	60370	AVPI1	mRNA	2.29054	3.30E-64
44	54218	B3Galt4	mRNA	0.7529	0.003048	8705	B3GALT4	mRNA	1.98563	2.30E-10
45	116848	Baz2A	mRNA	0.4197	0.014031	11176	BAZ2A	mRNA	0.86298	8.35E-19
46	407823	Baz2B	mRNA	1.0187	0.005686	29994	BAZ2B	mRNA	1.02593	9.71E-22
47	170770	Bbc3	mRNA	0.8893	0.00353	27113	BBC3	mRNA	0.83489	6.92E-06
48	12042	Bcl10	mRNA	0.7357	1.81E-09	8915	BCL10	mRNA	0.32865	0.009768
49	12125	Bcl2L11	mRNA	1.1276	0.000241	10018	BCL2L11	mRNA	1.05597	8.10E-15
50	72567	Bclaf1	mRNA	0.2758	0.026109	9774	BCLAF1	mRNA	0.52271	3.41E-08
51	382252	Bclaf3	mRNA	1.4568	4.12E-13	256643	BCLAF3	mRNA	0.93535	2.88E-09
52	320376	Bcorl1	mRNA	0.4866	0.021715	63035	BCORL1	mRNA	0.8082	9.00E-07

53	12062	Bdkrb2	mRNA	0.9911	0.002332	624	BDKRB2	mRNA	2.68109	1.45E-07
54	30948	Bin1	mRNA	0.4645	0.000333	274	BIN1	mRNA	0.38773	0.000281
55	233016	Blvrb	mRNA	0.3912	0.029132	645	BLVRB	mRNA	0.41517	0.000562
56	66352	Blzf1	mRNA	0.9044	1.31E-08	8548	BLZF1	mRNA	0.27208	0.029835
57	12175	Bnip2	mRNA	0.3765	0.000737	663	BNIP2	mRNA	0.39246	3.43E-05
58	71923	Borcs6	mRNA	0.4809	0.033568	54785	BORCS6	mRNA	0.97599	6.07E-06
59	223770	Brd1	mRNA	0.5379	0.002599	23774	BRD1	mRNA	0.28454	0.01773
60	14312	Brd2	mRNA	0.9239	8.46E-07	6046	BRD2	mRNA	0.24604	0.002237
61	67382	Brd3	mRNA	0.3761	0.021435	8019	BRD3	mRNA	0.47352	7.98E-06
62	78656	Brd8	mRNA	0.3999	0.007459	10902	BRD8	mRNA	0.8126	2.52E-17
63	71678	Brox	mRNA	0.4438	0.002083	148362	BROX	mRNA	0.69326	2.89E-13
64	100383	Bsdc1	mRNA	1.0768	3.02E-10	55108	BSDC1	mRNA	0.67406	4.69E-13
65	12226	Btg1	mRNA	1.3448	1.18E-06	694	BTG1	mRNA	1.89943	3.16E-86
66	12237	Bub3	mRNA	0.2542	0.040218	9184	BUB3	mRNA	0.23099	0.009562
67	231889	Bud31	mRNA	0.625	0.000584	8896	BUD31	mRNA	0.38645	6.67E-05
68	66882	Bzw1	mRNA	0.5357	0.002766	9689	BZW1	mRNA	0.39511	5.66E-08
69	12283	Cab39	mRNA	0.4494	0.014942	51719	CAB39	mRNA	0.32287	0.00017
70	78832	Cacul1	mRNA	0.7326	4.52E-07	143384	CACUL1	mRNA	0.25842	0.012855
71	67488	Calcoco1	mRNA	0.9617	1.08E-09	57658	CALCOCO1	mRNA	0.91106	2.42E-15
72	67886	Camsap2	mRNA	0.5192	0.007485	23271	CAMSAP2	mRNA	0.19097	0.031914
73	12367	Casp3	mRNA	0.7878	1.09E-08	836	CASP3	mRNA	0.43332	1.27E-05
74	12380	Cast	mRNA	0.6479	3.16E-06	831	CAST	mRNA	0.31796	4.52E-05
75	12418	Cbx4	mRNA	0.8939	0.003964	8535	CBX4	mRNA	0.90075	2.32E-13
76	54648	Ccdc120	mRNA	0.8092	4.76E-05	90060	CCDC120	mRNA	0.63787	0.000287
77	213993	Ccdc186	mRNA	0.7373	0.006995	55088	CCDC186	mRNA	0.73718	1.52E-11
78	66396	Ccdc82	mRNA	0.414	0.028788	79780	CCDC82	mRNA	0.54175	1.57E-10
79	78317	Ccdc88B	mRNA	1.9142	0.040214	283234	CCDC88B	mRNA	1.24338	5.41E-06
80	12449	Ccnf	mRNA	0.5817	0.000115	899	CCNF	mRNA	0.51879	2.45E-05
81	12452	Ccng2	mRNA	2.0203	5.86E-10	901	CCNG2	mRNA	1.97521	1.25E-44
82	12527	Cd9	mRNA	0.6097	0.00398	928	CD9	mRNA	0.35471	2.47E-05
83	104252	Cdc42Ep2	mRNA	1.2261	3.88E-14	10435	CDC42EP2	mRNA	0.45226	0.001295
84	67849	Cdca5	mRNA	0.7042	1.32E-07	113130	CDCA5	mRNA	0.28978	0.006027
85	237459	Cdk17	mRNA	0.5388	0.014198	5128	CDK17	mRNA	0.65452	4.38E-10
86	78334	Cdk19	mRNA	0.5764	0.001221	23097	CDK19	mRNA	0.83215	1.13E-10
87	107951	Cdk9	mRNA	0.5196	1.40E-05	1025	CDK9	mRNA	0.50496	2.55E-07
88	12575	Cdkn1A	mRNA	1.5293	0.01579	1026	CDKN1A	mRNA	0.38588	1.63E-06
89	12576	Cdkn1B	mRNA	0.8034	1.35E-05	1027	CDKN1B	mRNA	0.52679	8.08E-07
90	70925	Cdkn2Aip	mRNA	0.8714	2.33E-10	55602	CDKN2AIP	mRNA	0.36081	0.008516
91	110911	Cds2	mRNA	0.5347	0.006927	8760	CDS2	mRNA	0.4163	5.21E-06
92	12608	Cebpb	mRNA	1.4904	1.19E-06	1051	CEBPB	mRNA	1.81324	7.50E-48
93	12609	Cebpd	mRNA	3.0458	4.37E-95	1052	CEBPD	mRNA	1.18952	1.07E-11
94	12611	Cebpg	mRNA	0.8713	1.99E-06	1054	CEBPG	mRNA	1.36599	2.99E-24
95	70454	Cenpl	mRNA	1.1667	4.68E-16	91687	CENPL	mRNA	0.82841	2.66E-08
96	225523	Cep120	mRNA	0.647	3.20E-06	153241	CEP120	mRNA	1.19122	2.91E-28
97	74201	Cep97	mRNA	0.6384	0.00255	79598	CEP97	mRNA	0.29821	0.046595
98	12632	Cfl2	mRNA	0.4535	0.016021	1073	CFL2	mRNA	0.40413	1.17E-05
99	12633	Cflar	mRNA	0.8736	2.95E-07	8837	CFLAR	mRNA	0.4549	5.59E-07
100	106143	Cggbp1	mRNA	0.6263	4.71E-08	8545	CGGBP1	mRNA	0.46893	1.10E-08
101	14004	Chchd2	mRNA	0.5581	4.93E-06	51142	CHCHD2	mRNA	0.39767	9.93E-06
102	107932	Chd4	mRNA	0.2893	0.043566	1108	CHD4	mRNA	0.30329	3.72E-05
103	67064	Chmp1B	mRNA	0.9474	8.20E-07	57132	CHMP1B	mRNA	2.43651	6.56E-152
104	76959	Chmp5	mRNA	0.6216	5.35E-06	51510	CHMP5	mRNA	0.84157	1.24E-17
105	56398	Chp1	mRNA	0.6367	7.07E-05	11261	CHP1	mRNA	0.6412	2.09E-13
106	68523	Ciao2B	mRNA	0.4713	0.020958	51647	CIAO2B	mRNA	0.92935	2.52E-18
107	66935	Cir1	mRNA	0.4359	0.002524	9541	CIR1	mRNA	0.82171	2.75E-13
108	54124	Cks1B	mRNA	0.4389	0.001066	1163	CKS1B	mRNA	0.58275	1.26E-10
109	66197	Cks2	mRNA	0.5297	6.16E-05	1164	CKS2	mRNA	0.41568	0.002577
110	29876	Clic4	mRNA	0.484	0.024543	25932	CLIC4	mRNA	0.31554	1.96E-05

111	76686	Clip3	mRNA	0.4552	0.02029	25999	CLIP3	mRNA	0.66246	0.000103
112	12750	Clk4	mRNA	0.6208	0.001137	57396	CLK4	mRNA	0.79473	1.56E-06
113	26374	Cop1	mRNA	0.267	0.047401	64326	COP1	mRNA	0.31074	0.007294
114	12848	Cops2	mRNA	0.4548	0.007466	9318	COPS2	mRNA	0.49088	4.07E-08
115	12864	Cox6C	mRNA	0.4047	0.021719	1345	COX6C	mRNA	0.40614	0.000229
116	12866	Cox7A2	mRNA	0.4001	0.042518	1347	COX7A2	mRNA	0.44861	0.000103
117	20463	Cox7A2L	mRNA	0.4412	0.006045	9167	COX7A2L	mRNA	0.62864	8.22E-13
118	231207	Cpeb2	mRNA	1.9373	1.64E-14	132864	CPEB2	mRNA	1.50476	5.59E-46
119	67579	Cpeb4	mRNA	1.3047	3.84E-06	80315	CPEB4	mRNA	0.36868	3.28E-05
120	58799	Crbn	mRNA	0.408	0.019513	51185	CRBN	mRNA	0.62828	3.81E-07
121	77128	Crebrf	mRNA	1.4321	2.53E-07	153222	CREBRF	mRNA	1.81733	1.94E-35
122	171508	Creld1	mRNA	1.3131	5.94E-16	78987	CRELD1	mRNA	0.47177	0.002111
123	12916	Crem	mRNA	2.0446	1.62E-17	1390	CREM	mRNA	0.73553	3.37E-07
124	56724	Cript	mRNA	0.4212	0.022052	9419	CRIPT	mRNA	0.62601	3.84E-06
125	12928	Crk	mRNA	0.4041	0.002154	1398	CRK	mRNA	0.34339	0.000198
126	12953	Cry2	mRNA	1.771	5.38E-05	1408	CRY2	mRNA	1.28769	3.14E-16
127	229663	Csde1	mRNA	0.5197	0.000217	7812	CSDE1	mRNA	0.2405	0.002107
128	104318	Csnk1D	mRNA	0.5704	9.30E-07	1453	CSNK1D	mRNA	0.34247	2.80E-05
129	27373	Csnk1E	mRNA	0.8301	1.60E-12	1454	CSNK1E	mRNA	0.65443	1.24E-12
130	70425	Csnk1G3	mRNA	0.5801	2.05E-05	1456	CSNK1G3	mRNA	0.46988	7.11E-05
131	13001	Csnk2B	mRNA	0.4087	0.000382	1460	CSNK2B	mRNA	0.3467	0.000199
132	207785	Csrnp2	mRNA	0.5222	0.002995	81566	CSRNP2	mRNA	0.67564	1.06E-08
133	227292	Ctdsp1	mRNA	0.4897	0.000208	58190	CTDSP1	mRNA	0.48073	1.01E-07
134	107869	Cth	mRNA	1	0.003054	1491	CTH	mRNA	3.43023	7.92E-88
135	26965	Cul1	mRNA	0.3099	0.003704	8454	CUL1	mRNA	0.21625	0.018099
136	72584	Cul4B	mRNA	0.3573	0.012781	8450	CUL4B	mRNA	0.25995	0.002822
137	20310	Cxcl2	mRNA	4.904	5.99E-11	2920	CXCL2	mRNA	1.26561	6.70E-10
138	13163	Daxx	mRNA	0.5194	0.002895	1616	DAXX	mRNA	0.69946	4.35E-15
139	227525	Dclre1C	mRNA	0.4981	0.008115	64421	DCLRE1C	mRNA	0.89038	1.04E-11
140	67665	Dctn4	mRNA	0.4141	0.008911	51164	DCTN4	mRNA	0.52035	3.36E-09
141	100737	Dcun1D4	mRNA	0.808	3.69E-08	23142	DCUN1D4	mRNA	0.31012	0.001716
142	66498	Dda1	mRNA	0.6491	9.01E-05	79016	DDA1	mRNA	0.26454	0.024251
143	51793	Ddah2	mRNA	0.6774	0.000762	23564	DDAH2	mRNA	0.71357	1.02E-07
144	13209	Ddx6	mRNA	0.2549	0.039529	1656	DDX6	mRNA	0.43895	1.34E-08
145	67379	Dedd2	mRNA	1.6032	6.15E-13	162989	DEDD2	mRNA	1.11492	2.31E-17
146	320560	Dennd5B	mRNA	0.5134	0.025715	160518	DENND5B	mRNA	0.88932	9.38E-11
147	67487	Dhx40	mRNA	1.0386	3.32E-18	79665	DHX40	mRNA	0.60814	6.57E-11
148	228836	Dlgap4	mRNA	0.7788	3.40E-06	22839	DLGAP4	mRNA	0.36833	0.00028
149	240283	Dmxl1	mRNA	0.4376	0.004385	1657	DMXL1	mRNA	0.71452	4.65E-14
150	81489	Dnajb1	mRNA	0.5994	0.000158	3337	DNAJB1	mRNA	0.46321	2.90E-05
151	27362	Dnajb9	mRNA	1.1772	1.90E-05	4189	DNAJB9	mRNA	0.45638	0.014536
152	75974	Dock11	mRNA	0.4836	0.001579	139818	DOCK11	mRNA	0.47287	0.008589
153	13486	Dr1	mRNA	0.3447	0.006045	1810	DR1	mRNA	0.27994	0.015505
154	80904	Dtx3	mRNA	0.566	0.018012	196403	DTX3	mRNA	0.43315	0.001293
155	207521	Dtx4	mRNA	0.9302	0.000686	23220	DTX4	mRNA	0.60334	0.002618
156	19252	Dusp1	mRNA	1.7495	3.72E-17	1843	DUSP1	mRNA	0.77793	4.79E-16
157	63953	Dusp10	mRNA	1.8215	3.16E-10	11221	DUSP10	mRNA	1.7515	3.47E-27
158	72102	Dusp11	mRNA	0.3775	0.00266	8446	DUSP11	mRNA	0.32852	0.018224
159	319520	Dusp4	mRNA	1.5694	1.57E-09	1846	DUSP4	mRNA	1.64939	9.73E-36
160	240672	Dusp5	mRNA	1.5967	1.14E-09	1847	DUSP5	mRNA	0.79853	1.87E-06
161	67603	Dusp6	mRNA	0.8181	0.001393	1848	DUSP6	mRNA	1.79529	2.93E-19
162	18218	Dusp8	mRNA	1.2876	0.000669	1850	DUSP8	mRNA	0.64591	6.82E-05
163	112403	Dxo	mRNA	0.5947	0.006567	1797	DXO	mRNA	0.43241	0.00142
164	13549	Dyrk1B	mRNA	1.4143	6.90E-05	9149	DYRK1B	mRNA	1.4237	8.44E-21
165	226419	Dyrk3	mRNA	0.486	0.033239	8444	DYRK3	mRNA	0.56613	5.97E-06
166	13627	Eef1A1	mRNA	0.3332	0.033276	1915	EEF1A1	mRNA	0.54925	7.92E-13
167	13629	Eef2	mRNA	0.3224	0.018506	1938	EEF2	mRNA	0.39049	2.26E-09
168	13653	Egr1	mRNA	1.0322	0.002995	1958	EGR1	mRNA	0.99874	0.038902

169	20918	Eif1	mRNA	0.9476	3.12E-11	10209	EIF1	mRNA	0.78933	1.27E-17
170	217715	Eif2B2	mRNA	0.6911	5.76E-08	8892	EIF2B2	mRNA	0.46219	1.17E-05
171	75705	Eif4B	mRNA	0.3462	0.002292	1975	EIF4B	mRNA	0.50009	2.15E-09
172	217869	Eif5	mRNA	0.2655	0.013811	1983	EIF5	mRNA	0.53212	1.05E-11
173	13709	Elf1	mRNA	0.5557	0.018676	1997	ELF1	mRNA	0.44732	0.000283
174	69257	Elf2	mRNA	0.3448	0.01664	1998	ELF2	mRNA	0.51378	3.35E-05
175	67673	Elob	mRNA	0.2718	0.020374	6923	ELOB	mRNA	0.38415	0.000247
176	13726	Emd	mRNA	0.3907	0.000753	2010	EMD	mRNA	0.42542	8.73E-05
177	56205	Ensa	mRNA	0.5228	4.06E-06	2029	ENSA	mRNA	0.24407	0.009387
178	13875	Erf	mRNA	0.7563	7.54E-06	2077	ERF	mRNA	0.57153	1.99E-07
179	77805	Esco1	mRNA	0.4716	0.022271	114799	ESCO1	mRNA	0.60456	2.12E-06
180	13885	Esd	mRNA	0.5402	0.000304	2098	ESD	mRNA	0.40372	1.33E-05
181	23872	Ets2	mRNA	1.2791	6.97E-05	2114	ETS2	mRNA	1.03476	7.34E-38
182	22350	Ezr	mRNA	1.2062	3.93E-17	7430	EZR	mRNA	0.54858	2.47E-08
183	16456	F11R	mRNA	0.9035	2.40E-06	50848	F11R	mRNA	0.63456	5.30E-09
184	225358	Fam13B	mRNA	0.8007	0.000148	51306	FAM13B	mRNA	0.3033	0.005179
185	71721	Fam13C	mRNA	1.1331	0.00072	220965	FAM13C	mRNA	1.06205	4.96E-10
186	102122	Fam192A	mRNA	0.3923	0.000794	80011	FAM192A	mRNA	0.2929	0.002155
187	230088	Fam214B	mRNA	1.4055	2.72E-12	80256	FAM214B	mRNA	1.39478	2.36E-47
188	67922	Fam32A	mRNA	0.4342	0.012699	26017	FAM32A	mRNA	0.27374	0.004311
189	66306	Fam53C	mRNA	0.62	0.000835	51307	FAM53C	mRNA	0.36152	0.000474
190	108900	Fam72A	mRNA	0.7206	0.001835	729533	FAM72A	mRNA	0.87038	3.34E-05
191	72826	Fam76B	mRNA	0.3892	0.005546	143684	FAM76B	mRNA	0.99616	3.87E-22
192	97863	Fam8A1	mRNA	0.4365	0.00262	51439	FAM8A1	mRNA	0.65717	7.89E-06
193	100040608	Fancf	mRNA	0.5973	0.001265	2188	FANCF	mRNA	0.53398	0.001478
194	14109	Fau	mRNA	0.4788	0.000591	2197	FAU	mRNA	0.41331	1.02E-06
195	14123	Fbrs	mRNA	0.6391	3.48E-05	64319	FBRS	mRNA	0.4843	2.27E-06
196	30843	Fbxl12	mRNA	0.5875	0.016206	54850	FBXL12	mRNA	0.36312	0.013708
197	50789	Fbxl3	mRNA	0.4797	0.000717	26224	FBXL3	mRNA	0.64638	7.03E-11
198	242960	Fbxl5	mRNA	0.7267	9.95E-05	26234	FBXL5	mRNA	0.47858	7.28E-07
199	225055	Fbxo11	mRNA	0.4457	0.017975	80204	FBXO11	mRNA	0.44081	7.73E-06
200	66822	Fbxo25	mRNA	0.3786	0.027714	26260	FBXO25	mRNA	1.01565	2.65E-18
201	67948	Fbxo28	mRNA	0.4816	0.000368	23219	FBXO28	mRNA	0.33803	0.002226
202	71865	Fbxo30	mRNA	1.2073	1.55E-17	84085	FBXO30	mRNA	0.71577	9.95E-10
203	218503	Fcho2	mRNA	0.4079	0.006096	115548	FCHO2	mRNA	0.77451	1.15E-09
204	14178	Fgf7	mRNA	1.1441	0.016253	2252	FGF7	mRNA	1.05708	0.01675
205	116701	Fgfrl1	mRNA	0.891	2.30E-07	53834	FGFRL1	mRNA	0.49612	6.62E-08
206	66437	Fis1	mRNA	0.487	0.000586	51024	FIS1	mRNA	0.49495	4.55E-05
207	216742	Fnip1	mRNA	0.6565	0.020348	96459	FNIP1	mRNA	0.29382	0.024316
208	17425	Foxk1	mRNA	0.4321	0.017976	221937	FOXK1	mRNA	0.43659	0.001591
209	54601	Foxo4	mRNA	0.4821	0.012132	4303	FOXO4	mRNA	0.69692	0.025546
210	74123	Foxp4	mRNA	0.4097	0.013801	116113	FOXP4	mRNA	0.34556	0.013457
211	14296	Frat1	mRNA	1.7856	0.011284	10023	FRAT1	mRNA	2.07994	0.001129
212	212398	Frat2	mRNA	3.012	2.49E-25	23401	FRAT2	mRNA	1.17789	1.16E-06
213	14300	Frg1	mRNA	0.4861	0.002982	2483	FRG1	mRNA	0.40121	0.000131
214	319636	Fsd1L	mRNA	0.5948	0.021744	83856	FSD1L	mRNA	0.61809	0.000321
215	14359	Fxr1	mRNA	0.5815	2.75E-05	8087	FXR1	mRNA	0.55574	1.17E-10
216	14360	Fyn	mRNA	0.6844	0.001318	2534	FYN	mRNA	0.93244	2.91E-17
217	14389	Gab2	mRNA	1.0433	7.87E-07	9846	GAB2	mRNA	0.98501	8.06E-23
218	56486	Gabarap	mRNA	0.5445	0.000902	11337	GABARAP	mRNA	0.49401	3.21E-08
219	57436	Gabarapl1	mRNA	1.1819	4.43E-11	23710	GABARAPI	mRNA	2.67928	1.24E-93
220	93739	Gabarapl2	mRNA	0.4905	0.000724	11345	GABARAPI	mRNA	0.44681	0.000132
221	14390	Gabpa	mRNA	0.302	0.016311	2551	GABPA	mRNA	0.36016	0.001152
222	14433	Gapdh	mRNA	0.3675	0.006939	2597	GAPDH	mRNA	0.34759	0.000457
223	229542	Gatad2B	mRNA	0.4347	0.000273	57459	GATAD2B	mRNA	0.62366	7.89E-09
224	74375	Gcc1	mRNA	0.459	0.026965	79571	GCC1	mRNA	0.30155	0.030294
225	14528	Gch1	mRNA	0.475	0.028753	2643	GCH1	mRNA	0.76439	1.00E-04
226	14629	Gclc	mRNA	0.9913	2.60E-11	2729	GCLC	mRNA	0.69392	3.40E-09

227	14630	Gclm	mRNA	0.2727	0.042561	2730	GCLM	mRNA	0.79065	3.31E-14
228	14567	Gdi1	mRNA	0.5278	3.40E-05	2664	GDI1	mRNA	0.3468	8.32E-05
229	217039	Ggnbp2	mRNA	0.7174	6.66E-09	79893	GGNBP2	mRNA	0.3879	8.55E-05
230	14593	Ggps1	mRNA	0.8205	2.28E-09	9453	GGPS1	mRNA	0.37367	0.007915
231	66092	Ghitm	mRNA	0.5822	0.000379	27069	GHITM	mRNA	0.41838	2.11E-05
232	63985	Gmfb	mRNA	0.6294	1.43E-06	2764	GMFB	mRNA	0.37924	9.60E-06
233	14707	Gng5	mRNA	0.4982	0.030783	2787	GNG5	mRNA	0.26883	0.016276
234	66629	Golph3	mRNA	0.5798	2.37E-05	64083	GOLPH3	mRNA	0.29957	0.001942
235	81845	Gpank1	mRNA	0.4911	0.039265	7918	GPANK1	mRNA	0.56413	0.000605
236	77110	Gpbp1L1	mRNA	0.9031	3.12E-10	60313	GPBP1L1	mRNA	0.57214	1.54E-08
237	83924	Gpr137B	mRNA	0.7463	0.000116	7107	GPR137B	mRNA	0.51054	0.010789
238	232431	Gprc5A	mRNA	0.2905	0.049437	9052	GPRC5A	mRNA	0.9059	1.89E-20
239	54645	Gripap1	mRNA	0.4929	0.006398	56850	GRIPAP1	mRNA	0.36785	0.007041
240	66787	Gskip	mRNA	0.4898	0.008211	51527	GSKIP	mRNA	0.45099	0.000404
241	14873	Gsto1	mRNA	1.24	1.66E-14	9446	GSTO1	mRNA	0.32881	0.000535
242	14884	Gtf2H1	mRNA	0.4303	0.029934	2965	GTF2H1	mRNA	0.22345	0.022767
243	57080	Gtf2Ird1	mRNA	0.5775	0.009124	9569	GTF2IRD1	mRNA	1.54116	1.02E-65
244	74533	Gzf1	mRNA	0.825	1.57E-09	64412	GZF1	mRNA	0.47067	0.000124
245	232440	H2Afj	mRNA	0.5731	2.20E-05	55766	H2AFJ	mRNA	0.50501	0.01207
246	51788	H2Afz	mRNA	0.7298	6.06E-05	3015	H2AFZ	mRNA	0.28722	0.007045
247	231123	Haus3	mRNA	0.4985	0.004378	79441	HAUS3	mRNA	0.31646	0.034235
248	73389	Hbp1	mRNA	1.6309	1.59E-11	26959	HBP1	mRNA	1.8616	5.19E-64
249	380629	Heca	mRNA	1.3656	4.11E-15	51696	HECA	mRNA	1.26346	1.47E-22
250	80517	Herpud2	mRNA	0.6853	0.000272	64224	HERPUD2	mRNA	0.63883	2.49E-09
251	192231	Hexim1	mRNA	0.9046	4.43E-09	10614	HEXIM1	mRNA	0.42776	1.29E-05
252	102423	Hinfp	mRNA	0.4433	0.002933	25988	HINFP	mRNA	0.6486	7.49E-06
253	15259	Hipk3	mRNA	0.5332	0.001633	10114	HIPK3	mRNA	0.30016	0.001183
254	219150	Hmbox1	mRNA	0.6706	0.000186	79618	HMBOX1	mRNA	0.52513	4.77E-05
255	15368	Hmox1	mRNA	1.4972	1.16E-05	3162	HMOX1	mRNA	1.67726	6.72E-06
256	15381	Hnrnpc	mRNA	0.3816	0.032203	3183	HNRNPC	mRNA	0.26434	0.006753
257	72692	Hnrnpll	mRNA	0.2998	0.037035	92906	HNRNPLL	mRNA	0.60052	7.65E-07
258	110920	Hspa13	mRNA	0.5289	0.000743	6782	HSPA13	mRNA	0.34132	0.000937
259	80888	Hspb8	mRNA	0.8609	9.45E-07	26353	HSPB8	mRNA	0.31788	0.008868
260	76832	Hyls1	mRNA	0.8663	4.90E-09	219844	HYLS1	mRNA	0.42853	0.000686
261	15936	Ier2	mRNA	1.1564	0.000156	9592	IER2	mRNA	0.93983	5.15E-18
262	15937	Ier3	mRNA	2.9589	4.23E-25	8870	IER3	mRNA	1.36377	6.17E-47
263	15982	Ifrd1	mRNA	1.153	4.18E-11	3475	IFRD1	mRNA	1.32205	2.64E-36
264	55978	Ift20	mRNA	0.5065	0.017396	90410	IFT20	mRNA	0.42607	0.019353
265	67143	Ikzf5	mRNA	0.5917	1.33E-05	64376	IKZF5	mRNA	0.47624	0.000441
266	16156	Il11	mRNA	3.2333	5.67E-11	3589	IL11	mRNA	4.45539	1.94E-77
267	93757	Immp2L	mRNA	0.6437	0.014198	83943	IMMP2L	mRNA	0.51048	0.025524
268	26356	Ing1	mRNA	0.4772	0.000269	3621	ING1	mRNA	0.73497	3.65E-05
269	28019	Ing4	mRNA	0.5348	0.004751	51147	ING4	mRNA	1.14272	4.18E-14
270	76500	Ip6K2	mRNA	0.6421	0.007185	51447	IP6K2	mRNA	0.38435	0.000334
271	108960	Irak2	mRNA	1.3539	2.26E-14	3656	IRAK2	mRNA	1.59955	6.62E-33
272	238330	Irf2Bpl	mRNA	0.8155	0.018426	64207	IRF2BPL	mRNA	1.29893	2.10E-28
273	414801	Itprip	mRNA	1.8713	8.90E-15	85450	ITPRIP	mRNA	2.0786	1.04E-83
274	16449	Jag1	mRNA	0.5949	0.030374	182	JAG1	mRNA	1.07104	2.38E-18
275	57748	Jmy	mRNA	0.8084	0.006276	133746	JMY	mRNA	1.41623	1.22E-22
276	16477	Junb	mRNA	1.3928	3.41E-16	3726	JUNB	mRNA	0.88898	2.06E-15
277	16478	Jund	mRNA	1.3528	8.89E-09	3727	JUND	mRNA	0.96999	2.55E-16
278	68691	Kansl1L	mRNA	1.3098	0.000246	151050	KANSL1L	mRNA	0.48913	0.004975
279	81601	Kat5	mRNA	0.3476	0.009669	10524	KAT5	mRNA	0.20929	0.049178
280	210973	Kbtbd2	mRNA	0.5846	0.000175	25948	KBTBD2	mRNA	0.225	0.031163
281	74287	Kcmf1	mRNA	0.3588	0.008491	56888	KCMF1	mRNA	0.39151	0.000116
282	622320	Kctd21	mRNA	1.2162	0.048434	283219	KCTD21	mRNA	0.78342	9.98E-09
283	105440	Kctd9	mRNA	0.3582	0.020727	54793	KCTD9	mRNA	0.52906	5.65E-09
284	277250	Kdm3B	mRNA	0.3527	0.048331	51780	KDM3B	mRNA	0.26667	0.00669

285	214899	Kdm5A	mRNA	0.3949	0.005316	5927	KDM5A	mRNA	0.49344	2.99E-07
286	216850	Kdm6B	mRNA	1.3041	0.000817	23135	KDM6B	mRNA	1.02202	1.08E-07
287	73804	Kif2C	mRNA	0.507	5.71E-05	11004	KIF2C	mRNA	0.33336	0.000186
288	16579	Kifap3	mRNA	0.5226	0.000152	22920	KIFAP3	mRNA	0.62101	3.58E-09
289	74764	Klc4	mRNA	0.7944	0.000403	89953	KLC4	mRNA	0.93704	6.54E-08
290	194655	Klf11	mRNA	1.6481	7.14E-11	8462	KLF11	mRNA	0.53179	4.12E-05
291	50794	Klf13	mRNA	1.1477	1.01E-05	51621	KLF13	mRNA	0.50322	5.20E-06
292	16598	Klf2	mRNA	1.9391	1.17E-29	10365	KLF2	mRNA	4.00454	1.94E-125
293	16599	Klf3	mRNA	0.6327	0.000857	51274	KLF3	mRNA	0.82378	4.05E-13
294	16600	Klf4	mRNA	2.462	2.23E-05	9314	KLF4	mRNA	0.65682	0.003279
295	23849	Klf6	mRNA	1.1541	1.07E-11	1316	KLF6	mRNA	1.53964	7.88E-76
296	69554	Klhdc2	mRNA	0.3345	0.029835	23588	KLHDC2	mRNA	1.19597	1.09E-26
297	71765	Klhdc3	mRNA	0.9053	1.41E-13	116138	KLHDC3	mRNA	0.50421	7.19E-07
298	75785	Klhl24	mRNA	1.6694	0.001729	54800	KLHL24	mRNA	1.96774	2.05E-34
299	66689	Klhl28	mRNA	0.7806	0.000602	54813	KLHL28	mRNA	1.13751	9.81E-14
300	67956	Kmt5A	mRNA	0.4651	1.57E-05	387893	KMT5A	mRNA	0.67833	2.25E-13
301	16646	Kpna1	mRNA	0.5443	1.14E-06	3836	KPNA1	mRNA	0.18011	0.043402
302	16649	Kpna4	mRNA	0.2921	0.032549	3840	KPNA4	mRNA	0.3782	1.27E-05
303	16653	Kras	mRNA	0.6337	1.14E-08	3845	KRAS	mRNA	1.6204	1.78E-71
304	16668	Krt18	mRNA	1.9295	0.000309	3875	KRT18	mRNA	0.5588	8.47E-13
305	16691	Krt8	mRNA	3.1352	5.67E-11	3856	KRT8	mRNA	0.32385	5.91E-06
306	74127	Krt80	mRNA	0.4603	0.01278	144501	KRT80	mRNA	0.46068	1.48E-08
307	28036	Larp7	mRNA	0.5585	0.000124	51574	LARP7	mRNA	0.23599	0.022251
308	16854	Lgals3	mRNA	0.4439	0.000743	3958	LGALS3	mRNA	0.53321	8.73E-05
309	319583	Lig4	mRNA	1.2679	3.15E-12	3981	LIG4	mRNA	0.6358	4.85E-08
310	231506	Lin54	mRNA	0.6311	4.97E-06	132660	LIN54	mRNA	0.39183	0.000473
311	56722	Litaf	mRNA	1.0075	5.88E-20	9516	LITAF	mRNA	0.99473	5.87E-28
312	74775	Lmbr1L	mRNA	0.9574	4.16E-05	55716	LMBR1L	mRNA	1.47701	4.38E-20
313	16911	Lmo4	mRNA	1.0914	0.003491	8543	LMO4	mRNA	0.73487	4.06E-14
314	244421	Lonrf1	mRNA	0.8352	0.02413	91694	LONRF1	mRNA	0.46981	0.014054
315	53978	Lpar2	mRNA	0.5546	0.000721	9170	LPAR2	mRNA	1.39866	1.24E-09
316	241296	Lrrc8A	mRNA	0.7867	0.003234	56262	LRRC8A	mRNA	0.46647	3.28E-08
317	66725	Lrrk2	mRNA	0.8985	6.97E-05	120892	LRRK2	mRNA	1.96106	1.28E-32
318	80289	Lysmd3	mRNA	0.714	1.62E-07	116068	LYSMD3	mRNA	0.28319	0.019696
319	241638	Lzts3	mRNA	1.0106	5.21E-08	9762	LZTS3	mRNA	0.51375	0.006865
320	66146	Maco1	mRNA	0.9347	4.76E-12	55219	MACO1	mRNA	0.44678	0.000148
321	66591	Mad2L1B	mRNA	0.6404	9.15E-05	9587	MAD2L1BP	mRNA	0.3497	0.019149
322	68877	Maf1	mRNA	0.8265	2.50E-08	84232	MAF1	mRNA	0.76671	2.65E-18
323	26406	Map3K3	mRNA	0.6457	2.96E-05	4215	MAP3K3	mRNA	0.29301	0.018647
324	213582	Map9	mRNA	0.6081	0.003087	79884	MAP9	mRNA	0.38216	0.006817
325	50772	Mapk6	mRNA	0.6217	0.002924	5597	MAPK6	mRNA	0.26693	0.001368
326	26419	Mapk8	mRNA	0.3861	0.018431	5599	MAPK8	mRNA	0.28066	0.019764
327	17193	Mbd4	mRNA	0.6483	0.000712	8930	MBD4	mRNA	0.61474	4.17E-09
328	109241	Mbd5	mRNA	1.0332	0.001197	55777	MBD5	mRNA	0.96431	3.60E-09
329	110962	Mbd6	mRNA	1.0012	0.00073	114785	MBD6	mRNA	0.59248	7.02E-05
330	103537	Mbtd1	mRNA	1.1971	1.81E-10	54799	MBTD1	mRNA	0.54722	0.000406
331	231803	Mepce	mRNA	1.0182	5.29E-14	56257	MEPCE	mRNA	0.27144	0.002747
332	210029	Metrnl	mRNA	1.3545	3.30E-26	284207	METRNL	mRNA	0.49278	0.017694
333	67011	Mettl6	mRNA	0.5621	0.000229	131965	METTL6	mRNA	0.28453	0.045648
334	240396	Mex3C	mRNA	0.5809	0.000253	51320	MEX3C	mRNA	0.60269	1.80E-08
335	75734	Mff	mRNA	0.3328	0.048693	56947	MFF	mRNA	0.30133	0.001961
336	78506	Micu3	mRNA	1.1983	1.91E-06	286097	MICU3	mRNA	0.50186	0.00359
337	59090	Midn	mRNA	0.4082	0.03181	90007	MIDN	mRNA	1.01593	9.13E-12
338	71148	Mier1	mRNA	0.8756	1.43E-10	57708	MIER1	mRNA	0.31777	0.001891
339	56772	Mllt11	mRNA	1.0494	4.22E-09	10962	MLLT11	mRNA	0.96334	1.58E-11
340	17428	Mnt	mRNA	1.0136	0.001021	4335	MNT	mRNA	0.42238	0.011308
341	68473	Mob1B	mRNA	0.7349	4.51E-08	92597	MOB1B	mRNA	0.41137	7.35E-05
342	101513	Mob2	mRNA	0.5182	0.0013	81532	MOB2	mRNA	0.86921	6.57E-11

343	70380	Mospd1	mRNA	0.5688	0.021297	56180	MOSPD1	mRNA	0.27192	0.040417
344	76763	Mospd2	mRNA	0.4716	0.00273	158747	MOSPD2	mRNA	0.28358	0.044349
345	17686	Msh3	mRNA	0.6423	0.000836	4437	MSH3	mRNA	0.26624	0.035029
346	74026	Msl1	mRNA	0.6308	8.52E-05	339287	MSL1	mRNA	0.53565	1.76E-09
347	97287	Mtmr14	mRNA	0.5538	0.000491	64419	MTMR14	mRNA	0.54415	1.70E-07
348	14489	Mtpn	mRNA	0.5229	5.21E-05	136319	MTPN	mRNA	0.22523	0.010951
349	17119	Mxd1	mRNA	0.9039	0.005205	4084	MXD1	mRNA	0.95187	6.47E-10
350	17122	Mxd4	mRNA	0.845	0.000304	10608	MXD4	mRNA	1.16308	4.53E-28
351	50918	Myadm	mRNA	0.3444	0.004977	91663	MYADM	mRNA	0.27383	0.001056
352	17864	Mybl1	mRNA	0.5636	0.000836	4603	MYBL1	mRNA	0.29508	0.021103
353	17876	Myef2	mRNA	0.407	0.000918	50804	MYEF2	mRNA	0.41945	2.99E-07
354	17904	Myl6	mRNA	0.407	0.015742	4637	MYL6	mRNA	0.45155	4.85E-06
355	333789	N4Bp2	mRNA	1.1104	1.68E-10	55728	N4BP2	mRNA	0.43078	0.007033
356	100637	N4Bp2L1	mRNA	1.4571	0.000192	90634	N4BP2L1	mRNA	1.44124	1.15E-11
357	24083	Natd1	mRNA	0.5441	0.007146	256302	NATD1	mRNA	1.22657	2.21E-08
358	17966	Nbr1	mRNA	0.6788	0.001313	4077	NBR1	mRNA	0.7759	2.31E-22
359	17974	Nck2	mRNA	0.7675	0.000547	8440	NCK2	mRNA	0.3604	0.003696
360	380969	Nckap5L	mRNA	0.8393	0.000399	57701	NCKAP5L	mRNA	0.67374	3.28E-07
361	83431	Ndel1	mRNA	0.8944	4.11E-07	81565	NDEL1	mRNA	0.24975	0.030981
362	54405	Ndufa1	mRNA	0.5033	0.007346	4694	NDUFA1	mRNA	0.48401	0.00018
363	67184	Ndufa13	mRNA	0.4534	0.000593	51079	NDUFA13	mRNA	0.31177	0.002088
364	17991	Ndufa2	mRNA	0.5674	0.001408	4695	NDUFA2	mRNA	0.41205	0.002851
365	17992	Ndufa4	mRNA	0.4078	0.027167	4697	NDUFA4	mRNA	0.33818	0.002221
366	67130	Ndufa6	mRNA	0.4224	0.019743	4700	NDUFA6	mRNA	0.37564	0.002899
367	104130	Ndufb11	mRNA	0.4782	0.014064	54539	NDUFB11	mRNA	0.31143	0.015815
368	68198	Ndufb2	mRNA	0.4807	0.005389	4708	NDUFB2	mRNA	0.46144	0.000146
369	54446	Nfat5	mRNA	0.8485	0.004184	10725	NFAT5	mRNA	0.39735	0.004226
370	18024	Nfe2L2	mRNA	1.1512	1.44E-05	4780	NFE2L2	mRNA	0.7551	6.35E-17
371	18035	Nfkbia	mRNA	1.035	3.71E-06	4792	NFKBIA	mRNA	0.39306	1.87E-05
372	18038	Nfkbil1	mRNA	0.471	0.007943	4795	NFKBIL1	mRNA	0.96519	6.41E-11
373	270156	Nkapd1	mRNA	0.4159	0.045805	55216	NKAPD1	mRNA	0.41516	3.25E-05
374	107607	Nod1	mRNA	0.9349	0.002843	10392	NOD1	mRNA	0.53757	0.001896
375	68077	Nop53	mRNA	0.4844	0.000194	29997	NOP53	mRNA	0.44355	4.74E-08
376	19155	Npepps	mRNA	0.8799	1.55E-06	9520	NPEPPS	mRNA	0.23017	0.019827
377	353187	Nr1D2	mRNA	0.871	1.55E-09	9975	NR1D2	mRNA	0.638	7.94E-07
378	22260	Nr1H2	mRNA	0.8689	4.01E-11	7376	NR1H2	mRNA	0.27128	0.033435
379	15370	Nr4A1	mRNA	4.8878	1.33E-217	3164	NR4A1	mRNA	0.90043	1.52E-06
380	18124	Nr4A3	mRNA	3.2014	5.93E-29	8013	NR4A3	mRNA	5.74591	6.41E-26
381	18222	Numb	mRNA	0.8744	4.38E-07	8650	NUMB	mRNA	0.34918	0.000301
382	53319	Nxf1	mRNA	0.5024	2.01E-06	10482	NXF1	mRNA	0.34638	9.74E-05
383	237082	Nxt2	mRNA	0.4613	0.016972	55916	NXT2	mRNA	0.3645	0.033454
384	18245	Oaz1	mRNA	0.3227	0.00398	4946	OAZ1	mRNA	0.49215	2.17E-07
385	18263	Odc1	mRNA	1.1054	3.98E-10	4953	ODC1	mRNA	1.40789	4.67E-81
386	108155	Ogt	mRNA	0.4902	0.000764	8473	OGT	mRNA	0.31027	0.010407
387	100273	Osbpl9	mRNA	0.5904	0.001087	114883	OSBPL9	mRNA	0.37158	7.75E-06
388	66680	Oser1	mRNA	1.0673	2.23E-09	51526	OSER1	mRNA	0.63507	4.85E-08
389	209212	Osgin2	mRNA	1.3034	2.24E-06	734	OSGIN2	mRNA	0.30137	0.02095
390	54644	Otud5	mRNA	0.5147	0.000148	55593	OTUD5	mRNA	0.62213	4.41E-12
391	67869	Paip2	mRNA	0.6646	2.75E-07	51247	PAIP2	mRNA	0.54597	1.71E-06
392	18479	Pak1	mRNA	0.3755	0.009554	5058	PAK1	mRNA	0.25389	0.014162
393	72587	Pan3	mRNA	0.6728	0.001092	255967	PAN3	mRNA	0.97875	5.25E-21
394	211347	Pank3	mRNA	0.855	5.88E-08	79646	PANK3	mRNA	0.26589	0.022716
395	18514	Pbx1	mRNA	1.0571	0.000186	5087	PBX1	mRNA	1.31879	7.92E-14
396	74737	Pcf11	mRNA	0.3402	0.008039	51585	PCF11	mRNA	0.97544	2.82E-30
397	245867	Pcmtd2	mRNA	0.828	3.83E-09	55251	PCMTD2	mRNA	0.67283	4.87E-06
398	56426	Pdcd10	mRNA	0.4618	0.013553	11235	PDCD10	mRNA	0.48716	4.11E-05
399	18569	Pdcd4	mRNA	0.5437	0.01149	27250	PDCD4	mRNA	1.4573	2.44E-43
400	18578	Pde4B	mRNA	1.4096	9.73E-23	5142	PDE4B	mRNA	3.20616	1.05E-22

401	238871	Pde4D	mRNA	2.2145	4.72E-23	5144	PDE4D	mRNA	4.01078	2.43E-19
402	18584	Pde8A	mRNA	1.0484	0.038997	5151	PDE8A	mRNA	0.26747	0.019902
403	27273	Pdk4	mRNA	0.6045	0.006888	5166	PDK4	mRNA	2.53605	1.75E-72
404	18626	Per1	mRNA	2.397	3.01E-17	5187	PER1	mRNA	1.5906	1.96E-38
405	64058	Perp	mRNA	0.3457	0.015597	64065	PERP	mRNA	0.24616	0.012722
406	18643	Pfn1	mRNA	0.3029	0.029823	5216	PFN1	mRNA	0.32289	0.003113
407	110208	Pgd	mRNA	0.5377	0.002515	5226	PGD	mRNA	0.23073	0.008113
408	66522	Pgpep1	mRNA	1.0209	2.87E-09	54858	PGPEP1	mRNA	1.3456	1.18E-17
409	21652	Phf1	mRNA	0.8596	0.000429	5252	PHF1	mRNA	0.36748	0.009946
410	268448	Phf12	mRNA	0.3756	0.021396	57649	PHF12	mRNA	0.30454	0.008187
411	78246	Phf23	mRNA	0.9404	2.50E-15	79142	PHF23	mRNA	0.4978	3.42E-07
412	213109	Phf3	mRNA	0.6522	9.55E-05	23469	PHF3	mRNA	0.61458	2.58E-15
413	83946	Phip	mRNA	1.1	1.35E-11	55023	PHIP	mRNA	0.24306	0.019415
414	21664	Phlda1	mRNA	2.0056	1.62E-54	22822	PHLDA1	mRNA	2.44201	3.49E-70
415	84095	Pi4K2A	mRNA	0.7429	6.50E-07	55361	PI4K2A	mRNA	0.73533	2.03E-14
416	18706	Pik3Ca	mRNA	0.4553	0.008888	5290	PIK3CA	mRNA	0.7314	2.23E-10
417	18712	Pim1	mRNA	1.3449	8.91E-15	5292	PIM1	mRNA	1.79975	3.08E-44
418	117150	Pip4K2C	mRNA	0.5594	2.22E-05	79837	PIP4K2C	mRNA	0.44413	0.000673
419	219024	Pip4P1	mRNA	0.7672	7.72E-05	90809	PIP4P1	mRNA	0.75013	1.36E-11
420	241075	Plekhm3	mRNA	1.338	0.000128	389072	PLEKHM3	mRNA	0.68118	5.27E-05
421	102595	Plekho2	mRNA	0.7913	0.000147	80301	PLEKHO2	mRNA	1.28268	1.56E-33
422	12795	Plk3	mRNA	2.4543	0.001264	1263	PLK3	mRNA	0.53838	0.00046
423	66853	Pnpla2	mRNA	0.6348	0.000198	57104	PNPLA2	mRNA	1.0309	9.05E-31
424	67452	Pnpla8	mRNA	1.0865	1.21E-10	50640	PNPLA8	mRNA	1.30634	3.22E-31
425	108767	Pnrc1	mRNA	2.5246	7.87E-07	10957	PNRC1	mRNA	1.59943	6.76E-38
426	268373	Ppia	mRNA	0.3996	0.013063	5478	PPIA	mRNA	0.30606	0.000313
427	19042	Ppm1A	mRNA	0.672	3.42E-06	5494	PPM1A	mRNA	0.61299	6.37E-11
428	19043	Ppm1B	mRNA	0.5331	4.17E-05	5495	PPM1B	mRNA	0.68675	5.58E-09
429	19046	Ppp1Cb	mRNA	0.6179	3.01E-05	5500	PPP1CB	mRNA	0.92951	3.41E-35
430	52040	Ppp1R10	mRNA	0.4142	0.01278	5514	PPP1R10	mRNA	0.55132	1.47E-07
431	76448	Ppp1R18	mRNA	0.5856	2.97E-06	170954	PPP1R18	mRNA	0.60202	1.20E-12
432	66849	Ppp1R2	mRNA	0.401	0.000569	5504	PPP1R2	mRNA	0.38096	0.005546
433	69871	Ppp1R35	mRNA	0.7842	0.000517	221908	PPP1R35	mRNA	0.82052	4.86E-05
434	19052	Ppp2Ca	mRNA	0.4862	1.06E-05	5515	PPP2CA	mRNA	0.22137	0.042182
435	19053	Ppp2Cb	mRNA	0.4358	0.00021	5516	PPP2CB	mRNA	0.29179	0.00349
436	67857	Ppp6C	mRNA	0.6886	3.02E-06	5537	PPP6C	mRNA	0.21875	0.048236
437	72843	Prdm4	mRNA	0.8477	1.88E-07	11108	PRDM4	mRNA	0.22878	0.04791
438	54683	Prdx5	mRNA	0.7207	1.60E-10	25824	PRDX5	mRNA	0.6552	7.82E-11
439	18753	Prkcd	mRNA	0.3978	0.002319	5580	PRKCD	mRNA	0.67233	2.47E-09
440	15468	Prmt2	mRNA	0.5518	0.001111	3275	PRMT2	mRNA	0.20419	0.031092
441	229589	Prune1	mRNA	0.5498	0.000633	58497	PRUNE1	mRNA	0.48765	0.001871
442	67151	Psmd9	mRNA	0.3621	0.013234	5715	PSMD9	mRNA	0.38444	0.001598
443	56195	Ptbp2	mRNA	0.5516	4.16E-05	58155	PTBP2	mRNA	0.52907	4.87E-05
444	19243	Ptp4A1	mRNA	0.9729	1.36E-13	7803	PTP4A1	mRNA	0.9461	9.45E-27
445	19248	Ptpn12	mRNA	1.0593	2.45E-09	5782	PTPN12	mRNA	0.43196	6.55E-08
446	19258	Ptpn4	mRNA	0.304	0.040451	5775	PTPN4	mRNA	0.60033	4.76E-06
447	19338	Rab33B	mRNA	0.7323	1.75E-05	83452	RAB33B	mRNA	0.58305	0.00013
448	69834	Rab43	mRNA	0.3844	0.027079	339122	RAB43	mRNA	1.08474	1.97E-05
449	271457	Rab5A	mRNA	0.6602	2.23E-10	5868	RAB5A	mRNA	0.64132	1.41E-10
450	19346	Rab6A	mRNA	0.3811	0.001654	5870	RAB6A	mRNA	0.59803	5.92E-14
451	227800	Rabgap1	mRNA	0.5528	2.95E-05	23637	RABGAP1	mRNA	0.27047	0.010204
452	56705	Ranbp9	mRNA	0.6531	1.54E-05	10048	RANBP9	mRNA	0.22156	0.046626
453	76089	Rapgef2	mRNA	0.6946	0.004804	9693	RAPGEF2	mRNA	0.28221	0.012264
454	19411	Rarg	mRNA	0.5967	0.019964	5916	RARG	mRNA	1.0793	1.05E-14
455	56289	Rassf1	mRNA	0.7817	7.14E-07	11186	RASSF1	mRNA	1.2199	6.46E-41
456	12421	Rb1Cc1	mRNA	0.7267	2.96E-06	9821	RB1CC1	mRNA	1.27388	6.49E-48
457	19652	Rbm3	mRNA	0.3749	0.024208	5935	RBM3	mRNA	0.25594	0.001232
458	381626	Rbm33	mRNA	0.6723	0.000671	155435	RBM33	mRNA	0.41579	0.000383

459	170791	Rbm39	mRNA	0.3806	0.00033	9584	RBM39	mRNA	0.42547	5.95E-07
460	245945	Rbm47	mRNA	1.7754	0.000186	54502	RBM47	mRNA	0.52029	5.76E-07
461	319817	Rc3H2	mRNA	0.8646	2.36E-07	54542	RC3H2	mRNA	0.30204	0.001695
462	19697	Rela	mRNA	0.7367	5.99E-07	5970	RELA	mRNA	0.23345	0.005856
463	100532	Rell1	mRNA	0.5209	0.000998	768211	RELL1	mRNA	0.75805	2.78E-06
464	19735	Rgs2	mRNA	3.0377	1.85E-45	5997	RGS2	mRNA	2.03141	1.14E-07
465	69159	Rhebl1	mRNA	1.2263	0.000286	121268	RHEBL1	mRNA	1.41221	0.0054
466	11848	Rhoa	mRNA	0.5876	3.88E-08	387	RHOA	mRNA	0.83331	7.10E-25
467	11852	Rhob	mRNA	2.5573	4.20E-53	388	RHOB	mRNA	4.21878	1.78E-41
468	56212	Rhog	mRNA	0.4463	0.000529	391	RHOG	mRNA	0.26021	0.009794
469	19766	Ripk1	mRNA	0.791	1.01E-10	8737	RIPK1	mRNA	0.40334	9.69E-05
470	58809	Rnase4	mRNA	1.5185	7.84E-15	6038	RNASE4	mRNA	0.77439	1.54E-05
471	68031	Rnf146	mRNA	0.4985	0.001661	81847	RNF146	mRNA	0.73186	8.69E-10
472	30945	Rnf19A	mRNA	0.3448	0.013929	25897	RNF19A	mRNA	0.59743	2.30E-09
473	75234	Rnf19B	mRNA	1.0488	2.02E-15	127544	RNF19B	mRNA	1.19006	2.91E-18
474	268291	Rnf217	mRNA	1.1344	3.51E-15	154214	RNF217	mRNA	0.4161	0.000229
475	51902	Rnf24	mRNA	0.4109	0.007554	11237	RNF24	mRNA	0.63568	8.11E-10
476	57751	Rnf25	mRNA	0.5783	0.00857	64320	RNF25	mRNA	0.35212	0.00838
477	73469	Rnf38	mRNA	0.4373	0.00923	152006	RNF38	mRNA	0.59503	3.72E-07
478	67588	Rnf41	mRNA	0.5685	0.00238	10193	RNF41	mRNA	0.78784	1.21E-12
479	54197	Rnf5	mRNA	0.5116	0.000397	6048	RNF5	mRNA	0.58402	1.59E-09
480	67067	Romo1	mRNA	0.4377	0.010048	140823	ROMO1	mRNA	0.4549	0.01448
481	69723	Rpain	mRNA	0.7556	0.003152	84268	RPAIN	mRNA	0.5434	0.000201
482	110954	Rpl10	mRNA	0.4415	0.01611	6134	RPL10	mRNA	0.45015	1.10E-06
483	19896	Rpl10A	mRNA	0.4771	0.044437	4736	RPL10A	mRNA	0.63703	3.07E-18
484	67025	Rpl11	mRNA	0.3706	0.021277	6135	RPL11	mRNA	0.42682	2.37E-06
485	269261	Rpl12	mRNA	0.4203	0.010685	6136	RPL12	mRNA	0.63419	2.14E-15
486	22121	Rpl13A	mRNA	0.4653	0.022301	23521	RPL13A	mRNA	0.54503	3.57E-14
487	76808	Rpl18A	mRNA	0.442	0.013625	6142	RPL18A	mRNA	0.3335	0.000287
488	19933	Rpl21	mRNA	0.3935	0.032392	6144	RPL21	mRNA	0.54753	2.70E-12
489	68028	Rpl22L1	mRNA	0.6088	0.007683	200916	RPL22L1	mRNA	0.44137	1.91E-05
490	65019	Rpl23	mRNA	0.4156	0.008401	9349	RPL23	mRNA	0.45688	1.80E-07
491	268449	Rpl23A	mRNA	0.4118	0.010264	6147	RPL23A	mRNA	0.47214	3.64E-09
492	68193	Rpl24	mRNA	0.4522	0.001573	6152	RPL24	mRNA	0.38022	3.94E-05
493	19941	Rpl26	mRNA	0.5162	0.023859	6154	RPL26	mRNA	0.46253	2.16E-09
494	19943	Rpl28	mRNA	0.3032	0.02541	6158	RPL28	mRNA	0.30646	0.00244
495	19946	Rpl30	mRNA	0.4361	0.018012	6156	RPL30	mRNA	0.49489	2.53E-08
496	114641	Rpl31	mRNA	0.4367	0.020252	6160	RPL31	mRNA	0.55112	7.95E-13
497	68436	Rpl34	mRNA	0.5333	0.026713	6164	RPL34	mRNA	0.49692	1.73E-07
498	66489	Rpl35	mRNA	0.5161	0.014433	11224	RPL35	mRNA	0.40459	0.000323
499	54217	Rpl36	mRNA	0.4284	0.00803	25873	RPL36	mRNA	0.28581	0.002797
500	19982	Rpl36A	mRNA	0.4692	0.011464	6173	RPL36A	mRNA	0.41587	2.22E-06
501	67281	Rpl37	mRNA	0.4759	0.002345	6167	RPL37	mRNA	0.44656	6.06E-09
502	67671	Rpl38	mRNA	0.5819	0.00123	6169	RPL38	mRNA	0.38141	0.009377
503	67248	Rpl39	mRNA	0.471	0.017899	6170	RPL39	mRNA	0.47853	6.70E-10
504	67945	Rpl41	mRNA	0.3697	0.022117	6171	RPL41	mRNA	0.52997	2.34E-09
505	11837	Rplp0	mRNA	0.3529	0.01467	6175	RPLP0	mRNA	0.56783	1.51E-14
506	56040	Rplp1	mRNA	0.5332	0.000401	6176	RPLP1	mRNA	0.44939	1.73E-07
507	20042	Rps12	mRNA	0.4289	0.039795	6206	RPS12	mRNA	0.52313	2.61E-12
508	20044	Rps14	mRNA	0.4865	0.022749	6208	RPS14	mRNA	0.52831	1.16E-09
509	267019	Rps15A	mRNA	0.5006	0.026465	6210	RPS15A	mRNA	0.55668	7.52E-13
510	20055	Rps16	mRNA	0.4214	0.024156	6217	RPS16	mRNA	0.45305	4.85E-06
511	20068	Rps17	mRNA	0.5104	0.008879	6218	RPS17	mRNA	0.38716	6.76E-05
512	20084	Rps18	mRNA	0.489	0.035121	6222	RPS18	mRNA	0.49715	3.42E-13
513	20085	Rps19	mRNA	0.421	0.011023	6223	RPS19	mRNA	0.40416	4.75E-06
514	67427	Rps20	mRNA	0.5565	0.000167	6224	RPS20	mRNA	0.49767	3.03E-07
515	66481	Rps21	mRNA	0.3911	0.032015	6227	RPS21	mRNA	0.52242	1.62E-08
516	66475	Rps23	mRNA	0.3589	0.036361	6228	RPS23	mRNA	0.44441	2.37E-10

517	75617	Rps25	mRNA	0.4403	0.006546	6230	RPS25	mRNA	0.49052	1.41E-09
518	27370	Rps26	mRNA	0.3306	0.018907	6231	RPS26	mRNA	0.21798	0.030893
519	57294	Rps27	mRNA	0.5201	0.017899	6232	RPS27	mRNA	0.54935	1.36E-10
520	20090	Rps29	mRNA	0.4133	0.038625	6235	RPS29	mRNA	0.79441	1.66E-18
521	20102	Rps4X	mRNA	0.4503	0.048705	6191	RPS4X	mRNA	0.45547	1.05E-08
522	20103	Rps5	mRNA	0.3628	0.033794	6193	RPS5	mRNA	0.42066	3.35E-05
523	73086	Rps6Ka5	mRNA	1.2675	4.89E-07	9252	RPS6KA5	mRNA	0.92163	0.006003
524	76846	Rps9	mRNA	0.3105	0.031595	6203	RPS9	mRNA	0.42176	1.87E-07
525	229675	Rsbn1	mRNA	0.587	0.005059	54665	RSBN1	mRNA	0.41764	0.002616
526	233532	Rsf1	mRNA	0.2862	0.032446	51773	RSF1	mRNA	0.43697	4.79E-07
527	27981	Rsrp1	mRNA	1.1927	7.41E-06	57035	RSRP1	mRNA	0.36712	0.035411
528	56353	Rybp	mRNA	0.752	5.21E-07	23429	RYBP	mRNA	0.32462	0.012471
529	67630	Samd8	mRNA	0.3445	0.046895	142891	SAMD8	mRNA	0.29579	0.009005
530	66118	Sarnp	mRNA	0.5433	0.004897	84324	SARNP	mRNA	0.47004	1.84E-06
531	20229	Sat1	mRNA	2.8105	7.21E-34	6303	SAT1	mRNA	1.99885	3.10E-92
532	100126824	Sco2	mRNA	0.5661	1.03E-05	9997	SCO2	mRNA	0.55682	0.002514
533	228765	Sdcbp2	mRNA	1.5172	0.001388	27111	SDCBP2	mRNA	1.80555	0.0012
534	99683	Sec24B	mRNA	0.4394	0.001682	10427	SEC24B	mRNA	0.30836	0.002169
535	378702	Serf2	mRNA	0.3724	0.00042	10169	SERF2	mRNA	0.4281	1.66E-05
536	26943	Serinc3	mRNA	0.3708	0.016473	10955	SERINC3	mRNA	0.41859	2.27E-06
537	20713	Serpini1	mRNA	1.6888	6.00E-06	5274	SERPINI1	mRNA	2.5597	0.042493
538	228071	Sestd1	mRNA	0.8015	1.60E-07	91404	SESTD1	mRNA	0.39653	0.002775
539	72895	Setd5	mRNA	0.3945	0.012572	55209	SETD5	mRNA	0.29668	0.006878
540	67788	Sfr1	mRNA	0.7752	1.52E-09	119392	SFR1	mRNA	0.50973	0.017805
541	218544	Sgtb	mRNA	1.7624	3.88E-14	54557	SGTB	mRNA	1.18771	5.94E-26
542	54673	Sh3Glb1	mRNA	0.2469	0.032808	51100	SH3GLB1	mRNA	0.54228	4.69E-12
543	108037	Shmt2	mRNA	0.3807	0.042939	6472	SHMT2	mRNA	1.0279	3.43E-46
544	56392	Shoc2	mRNA	0.5825	0.000102	8036	SHOC2	mRNA	0.86005	5.82E-19
545	20466	Sin3A	mRNA	0.703	5.92E-07	25942	SIN3A	mRNA	0.50876	2.08E-08
546	93759	Sirt1	mRNA	0.7465	1.59E-08	23411	SIRT1	mRNA	0.56894	9.26E-07
547	20482	Skil	mRNA	0.567	0.005205	6498	SKIL	mRNA	0.65024	4.07E-13
548	18674	Slc25A3	mRNA	0.2968	0.007396	5250	SLC25A3	mRNA	0.34799	3.80E-05
549	67760	Slc38A2	mRNA	1.2701	0.020404	54407	SLC38A2	mRNA	1.88702	2.96E-06
550	17254	Slc3A2	mRNA	0.539	0.026846	6520	SLC3A2	mRNA	0.97253	3.70E-33
551	20874	Slk	mRNA	0.3482	0.021602	9748	SLK	mRNA	0.39804	7.46E-06
552	193116	Slu7	mRNA	0.547	0.002957	10569	SLU7	mRNA	0.69663	5.44E-13
553	17126	Smad2	mRNA	0.498	0.000174	4087	SMAD2	mRNA	1.32116	9.40E-55
554	57376	Smarce1	mRNA	0.2906	0.032853	6605	SMARCE1	mRNA	0.21439	0.019095
555	68552	Smim14	mRNA	0.3879	0.026255	201895	SMIM14	mRNA	1.84724	5.97E-51
556	228608	Smox	mRNA	0.4065	0.017976	54498	SMOX	mRNA	2.50285	1.80E-46
557	67797	Snrnp48	mRNA	0.6387	0.001336	154007	SNRNP48	mRNA	0.43967	0.000201
558	66354	Snw1	mRNA	0.3344	0.020827	22938	SNW1	mRNA	0.28466	0.001721
559	69024	Snx15	mRNA	0.4014	0.040306	29907	SNX15	mRNA	0.74323	1.40E-10
560	69178	Snx5	mRNA	0.3673	0.007011	27131	SNX5	mRNA	0.40164	3.18E-06
561	20662	Sos1	mRNA	0.3435	0.043687	6654	SOS1	mRNA	0.29597	0.005989
562	268301	Sowahc	mRNA	1.1831	1.69E-08	65124	SOWAHC	mRNA	0.61327	2.09E-05
563	70834	Spag9	mRNA	0.4233	0.014636	9043	SPAG9	mRNA	0.265	0.00141
564	20729	Spin1	mRNA	0.8352	2.42E-10	10927	SPIN1	mRNA	0.32246	0.000339
565	24064	Spry2	mRNA	0.6298	0.001012	10253	SPRY2	mRNA	0.58396	0.00064
566	76650	Srxn1	mRNA	0.969	1.52E-10	140809	SRXN1	mRNA	0.31181	0.00454
567	66970	Ssbp2	mRNA	0.5941	0.001815	23635	SSBP2	mRNA	0.44363	0.013706
568	20843	Stag2	mRNA	0.544	2.34E-05	10735	STAG2	mRNA	0.68417	5.35E-18
569	20460	Stil	mRNA	0.9907	1.54E-13	6491	STIL	mRNA	0.31405	0.012023
570	22218	Sumo1	mRNA	0.5234	0.017995	7341	SUMO1	mRNA	0.4781	3.11E-07
571	68592	Syf2	mRNA	0.5062	0.000442	25949	SYF2	mRNA	0.80658	3.77E-14
572	229521	Syt11	mRNA	0.6665	0.000484	23208	SYT11	mRNA	0.55488	1.79E-08
573	21351	Taldo1	mRNA	0.3587	0.002873	6888	TALDO1	mRNA	0.36684	0.000452
574	77097	Tanc2	mRNA	0.4309	0.04511	26115	TANC2	mRNA	1.09813	4.32E-32

575	21353	Tank	mRNA	0.847	1.97E-07	10010	TANK	mRNA	0.58351	3.16E-09
576	216965	Taok1	mRNA	0.4389	3.49E-05	57551	TAOK1	mRNA	0.247	0.00404
577	52440	Tax1Bp1	mRNA	0.4273	0.000612	8887	TAX1BP1	mRNA	0.47828	6.25E-08
578	103724	Tbc1D10/	mRNA	0.3534	0.032025	83874	TBC1D10A	mRNA	0.75306	1.07E-05
579	66687	Tbc1D15	mRNA	0.6457	0.000433	64786	TBC1D15	mRNA	0.38652	2.68E-05
580	233204	Tbc1D17	mRNA	0.7021	6.64E-05	79735	TBC1D17	mRNA	0.90194	2.92E-13
581	71310	Tbc1D9	mRNA	1.7287	7.88E-06	23158	TBC1D9	mRNA	0.28405	0.018689
582	21371	Tbca	mRNA	0.4762	0.029496	6902	TBCA	mRNA	0.23921	0.014591
583	56480	Tbk1	mRNA	0.4304	0.004582	29110	TBK1	mRNA	0.31082	0.001884
584	77574	Tcaf1	mRNA	0.5043	0.007952	9747	TCAF1	mRNA	0.27112	0.003069
585	216198	Tcp11L2	mRNA	2.5541	5.03E-11	255394	TCP11L2	mRNA	3.16306	1.63E-31
586	21685	Tef	mRNA	0.8265	0.000143	7008	TEF	mRNA	0.74554	0.006161
587	100715	Tent2	mRNA	0.4148	0.000992	167153	TENT2	mRNA	0.46831	1.51E-05
588	74645	Tent5C	mRNA	1.7211	0.003215	54855	TENT5C	mRNA	1.5462	0.000207
589	21753	Tes	mRNA	0.6192	0.001044	26136	TES	mRNA	0.30791	0.008924
590	209446	Tfe3	mRNA	0.5925	0.013262	7030	TFE3	mRNA	0.43381	7.43E-07
591	21815	Tgif1	mRNA	1.5294	4.49E-12	7050	TGIF1	mRNA	1.47956	1.69E-41
592	73754	Thap1	mRNA	0.5614	0.004014	55145	THAP1	mRNA	0.37438	0.028135
593	21824	Thbd	mRNA	2.3543	0.01568	7056	THBD	mRNA	1.13622	0.000197
594	28113	Tinf2	mRNA	0.4182	0.024783	26277	TINF2	mRNA	0.36615	0.001156
595	226591	Tiprl	mRNA	0.3692	0.032593	261726	TIPRL	mRNA	0.34528	0.001586
596	380712	Tlcd2	mRNA	1.7756	0.009063	727910	TLCD2	mRNA	0.74403	0.01024
597	24086	Tlk2	mRNA	0.5685	2.26E-05	11011	TLK2	mRNA	0.30433	0.002928
598	17112	Tm4Sf1	mRNA	0.6031	0.001342	4071	TM4SF1	mRNA	2.45028	2.43E-116
599	73130	Tmed5	mRNA	0.518	0.000112	50999	TMED5	mRNA	0.23016	0.014317
600	66309	Tmem128	mRNA	0.3844	0.012861	85013	TMEM128	mRNA	0.34259	0.009456
601	57439	Tmem183	mRNA	0.4783	5.97E-05	92703	TMEM183A	mRNA	0.48223	5.52E-07
602	19240	Tmsb10	mRNA	0.403	0.004544	9168	TMSB10	mRNA	0.52952	1.27E-09
603	21927	Tnfaip1	mRNA	0.3624	0.005461	7126	TNFAIP1	mRNA	0.47387	1.17E-08
604	51789	Tnk2	mRNA	0.4651	0.009792	10188	TNK2	mRNA	1.17099	2.74E-20
605	74493	Tnks2	mRNA	0.3372	0.026706	80351	TNKS2	mRNA	0.43219	4.47E-07
606	22057	Tob1	mRNA	0.7303	2.20E-05	10140	TOB1	mRNA	1.19934	6.62E-29
607	57259	Tob2	mRNA	1.0077	4.40E-06	10766	TOB2	mRNA	0.71569	1.22E-11
608	66169	Tomm7	mRNA	0.4299	0.011688	54543	TOMM7	mRNA	0.46404	1.34E-05
609	21983	Tpbg	mRNA	0.6696	1.81E-05	7162	TPBG	mRNA	0.90256	3.87E-08
610	22070	Tpt1	mRNA	0.6139	3.69E-05	7178	TPT1	mRNA	0.62143	4.23E-13
611	103213	Traf3Ip2	mRNA	0.6662	0.000732	10758	TRAF3IP2	mRNA	0.91652	5.50E-23
612	211770	Trib1	mRNA	1.3806	1.27E-09	10221	TRIB1	mRNA	0.58266	0.000959
613	94092	Trim16	mRNA	1.0994	1.11E-07	10626	TRIM16	mRNA	1.01537	2.78E-31
614	79263	Trim39	mRNA	0.436	0.009482	56658	TRIM39	mRNA	0.50431	0.001443
615	211007	Trim41	mRNA	0.8464	2.22E-08	90933	TRIM41	mRNA	0.39319	0.003255
616	106628	Trip10	mRNA	0.503	0.000309	9322	TRIP10	mRNA	0.21043	0.037936
617	74753	Trmo	mRNA	0.5299	0.01321	51531	TRMO	mRNA	0.51332	0.000345
618	67674	Trmt112	mRNA	0.319	0.010791	51504	TRMT112	mRNA	0.24961	0.008117
619	21807	Tsc22D1	mRNA	1.175	2.24E-26	8848	TSC22D1	mRNA	2.49205	7.07E-91
620	14605	Tsc22D3	mRNA	2.013	1.94E-14	1831	TSC22D3	mRNA	4.81731	0
621	78829	Tsc22D4	mRNA	0.4577	0.009737	81628	TSC22D4	mRNA	0.58449	0.000106
622	22088	Tsg101	mRNA	0.2992	0.030991	7251	TSG101	mRNA	0.65313	2.66E-11
623	67120	Ttc14	mRNA	1.0025	6.40E-23	151613	TTC14	mRNA	0.40515	0.00153
624	70387	Ttc9C	mRNA	0.5859	2.70E-05	283237	TTC9C	mRNA	0.84495	1.25E-11
625	70892	Ttll7	mRNA	0.824	9.35E-11	79739	TTLL7	mRNA	0.29711	0.032305
626	22142	Tuba1A	mRNA	1.2348	1.76E-08	7846	TUBA1A	mRNA	1.97245	1.44E-85
627	22151	Tubb2A	mRNA	2.4746	2.98E-87	7280	TUBB2A	mRNA	0.94713	4.64E-15
628	73710	Tubb2B	mRNA	2.858	2.31E-51	347733	TUBB2B	mRNA	2.04663	7.70E-08
629	214290	Tut7	mRNA	0.5586	0.000953	79670	TUT7	mRNA	0.52386	1.68E-06
630	319370	Ubald2	mRNA	0.6612	0.00074	283991	UBALD2	mRNA	1.74353	2.44E-40
631	67123	Ubap1	mRNA	1.28	1.81E-13	51271	UBAP1	mRNA	0.94276	1.46E-24
632	22187	Ubb	mRNA	0.9406	2.07E-10	7314	UBB	mRNA	0.40414	6.10E-05

633	22210	Ube2B	mRNA	0.5326	0.00129	7320	UBE2B	mRNA	0.98461	3.64E-20
634	66105	Ube2D3	mRNA	0.4231	0.004667	7323	UBE2D3	mRNA	0.28576	0.000171
635	22214	Ube2H	mRNA	0.5339	0.000907	7328	UBE2H	mRNA	0.93192	2.04E-30
636	67615	Ube2R2	mRNA	0.429	0.003332	54926	UBE2R2	mRNA	0.3822	4.44E-05
637	28018	Ubfd1	mRNA	0.5607	0.000604	56061	UBFD1	mRNA	0.28015	0.003049
638	66177	Ubl5	mRNA	0.4065	0.003161	59286	UBL5	mRNA	0.31262	0.005667
639	320538	Ubn2	mRNA	0.4683	0.037989	254048	UBN2	mRNA	0.71446	2.86E-05
640	327900	Ubtd2	mRNA	0.4477	0.01171	92181	UBTD2	mRNA	0.51402	4.52E-05
641	66530	Ubxn6	mRNA	0.494	0.000355	80700	UBXN6	mRNA	0.61047	4.50E-12
642	224111	Ubxn7	mRNA	0.7662	3.62E-10	26043	UBXN7	mRNA	0.53754	3.74E-07
643	109113	Uhrf2	mRNA	0.5413	0.000176	115426	UHRF2	mRNA	0.68589	5.24E-10
644	22241	Ulk1	mRNA	0.6188	0.008859	8408	ULK1	mRNA	0.95608	3.14E-12
645	66152	Uqcr10	mRNA	0.4435	0.01242	29796	UQCR10	mRNA	0.30037	0.027786
646	68205	Urm1	mRNA	0.4368	0.014357	81605	URM1	mRNA	0.24127	0.026375
647	22278	Usf1	mRNA	0.5552	9.69E-05	7391	USF1	mRNA	0.50969	2.06E-05
648	22282	Usf2	mRNA	0.3756	0.009283	7392	USF2	mRNA	0.37763	6.63E-05
649	22217	Usp12	mRNA	0.3052	0.031108	219333	USP12	mRNA	0.46313	2.13E-07
650	71472	Usp19	mRNA	0.6433	3.67E-07	10869	USP19	mRNA	0.59522	8.65E-11
651	244144	Usp35	mRNA	1.0273	7.14E-05	57558	USP35	mRNA	0.38917	0.028686
652	99526	Usp53	mRNA	1.0013	3.67E-09	54532	USP53	mRNA	1.22559	2.04E-37
653	22317	Vamp1	mRNA	0.9885	0.000713	6843	VAMP1	mRNA	0.61628	0.004506
654	26949	Vat1	mRNA	0.4383	0.000998	10493	VAT1	mRNA	0.45047	5.02E-08
655	22327	Vbp1	mRNA	0.3926	0.003122	7411	VBP1	mRNA	0.22547	0.047003
656	70675	Vcpip1	mRNA	0.6279	0.000877	80124	VCPIP1	mRNA	0.27174	0.029941
657	22337	Vdr	mRNA	0.8807	0.000186	7421	VDR	mRNA	0.50903	0.002737
658	22344	Vezf1	mRNA	0.4643	0.002051	7716	VEZF1	mRNA	0.66265	6.10E-14
659	71732	Vps11	mRNA	0.612	0.006099	55823	VPS11	mRNA	0.41152	1.65E-05
660	20479	Vps4B	mRNA	0.4843	3.16E-05	9525	VPS4B	mRNA	0.35414	0.000669
661	225131	Wac	mRNA	0.6311	1.21E-08	51322	WAC	mRNA	0.3046	0.00016
662	22378	Wbp2	mRNA	1.1023	3.13E-09	23558	WBP2	mRNA	0.35818	3.67E-05
663	226757	Wdr26	mRNA	0.506	8.22E-05	80232	WDR26	mRNA	0.44549	1.67E-07
664	54636	Wdr45	mRNA	0.8204	9.69E-05	11152	WDR45	mRNA	0.73659	3.76E-12
665	22390	Wee1	mRNA	0.6984	0.00011	7465	WEE1	mRNA	0.21046	0.044252
666	74781	Wipi2	mRNA	0.4773	0.014238	26100	WIPI2	mRNA	0.69553	9.42E-15
667	97064	Wwtr1	mRNA	0.5096	0.02528	25937	WWTR1	mRNA	0.63949	7.41E-14
668	22612	Yes1	mRNA	0.5428	0.000135	7525	YES1	mRNA	0.29371	0.000881
669	67864	Yipf4	mRNA	0.3608	0.026397	84272	YIPF4	mRNA	0.58564	1.34E-07
670	77864	Ypel2	mRNA	1.4361	0.00139	388403	YPEL2	mRNA	1.46803	7.52E-13
671	383295	Ypel5	mRNA	0.9735	4.22E-08	51646	YPEL5	mRNA	1.10319	2.01E-28
672	22629	Ywhah	mRNA	0.5899	9.77E-07	7533	YWHAH	mRNA	0.76721	1.30E-14
673	22631	Ywhaz	mRNA	0.6076	2.54E-08	7534	YWHAZ	mRNA	0.57986	1.48E-11
674	229055	Zbtb10	mRNA	1.0405	0.000457	65986	ZBTB10	mRNA	0.6251	3.85E-05
675	245007	Zbtb38	mRNA	0.5916	0.002069	253461	ZBTB38	mRNA	0.82438	9.02E-20
676	235132	Zbtb44	mRNA	0.5177	0.000103	29068	ZBTB44	mRNA	0.47988	5.08E-07
677	16969	Zbtb7A	mRNA	0.5083	0.000496	51341	ZBTB7A	mRNA	0.30184	0.003825
678	22724	Zbtb7B	mRNA	0.6165	0.007893	51043	ZBTB7B	mRNA	0.9465	2.98E-18
679	72350	Zc2Hc1C	mRNA	1.2904	3.99E-05	79696	ZC2HC1C	mRNA	0.90499	0.028252
680	24136	Zeb2	mRNA	0.7114	0.039233	9839	ZEB2	mRNA	0.48447	3.62E-07
681	100494	Zfand2A	mRNA	1.2691	3.58E-06	90637	ZFAND2A	mRNA	1.18407	7.62E-15
682	21769	Zfand3	mRNA	0.3084	0.013962	60685	ZFAND3	mRNA	0.45707	3.40E-07
683	22695	Zfp36	mRNA	0.8683	0.007144	7538	ZFP36	mRNA	1.15187	1.37E-11
684	22763	Zfr	mRNA	0.4752	0.047423	51663	ZFR	mRNA	0.41197	1.75E-07
685	217695	Zfyve1	mRNA	1.0191	3.71E-06	53349	ZFYVE1	mRNA	0.92489	1.43E-13
686	74570	Zkscan1	mRNA	0.343	0.044579	7586	ZKSCAN1	mRNA	0.31928	0.006935
687	22757	Zkscan5	mRNA	0.8223	0.000919	23660	ZKSCAN5	mRNA	0.48397	0.000135
688	328365	Zmiz1	mRNA	0.8024	0.00238	57178	ZMIZ1	mRNA	0.35563	0.000385
689	268721	Zswim8	mRNA	0.9289	5.75E-10	23053	ZSWIM8	mRNA	0.23995	0.033185
