# Supplementary material for: Promotion of axon regeneration and protection on injured retinal ganglion cells by rCXCL2
Source: Inflamm Regen. 2023 Jun 20;43:31. doi: 10.1186/s41232-023-00283-5 (PMC10280836; doi:10.1186/s41232-023-00283-5)
Supplement: Supplementary file 4 — Additional file 4: Table S2. There was a total of 595 co-downregulated genes in both groups of STS-treated cells. [file 41232_2023_283_MOESM4_ESM.pdf]

Table S2. There was a total of 595 co-downregulated genes in both groups of STS-treated cells.

	Gene ID	Symbol	Type	log2 (STS_661W / Vehicle_661W)	Qvalue (STS_661W / Vehicle_661W)	Gene ID	Symbol	Type	log2 (STS_ARPE19 / Vehicle_ARPE19)	Qvalue (STS_ARPE19 / Vehicle_ARPE19)
1	56321	Aatf	mRNA	-0.5594	0.000243	26574	AATF	mRNA	-0.6006	2.01E-09
2	109934	Abr	mRNA	-1.0438	5.88E-06	29	ABR	mRNA	-0.3559	1.21E-05
3	107476	Acaca	mRNA	-0.7189	0.0046975	31	ACACA	mRNA	-0.6858	3.63E-15
4	11428	Aco1	mRNA	-0.4514	0.00044	48	ACO1	mRNA	-0.2398	0.00625
5	242894	Actr3B	mRNA	-2.186	0.0469075	57180	ACTR3B	mRNA	-1.7949	7.25E-16
6	11487	Adam10	mRNA	-0.3239	0.029413	102	ADAM10	mRNA	-0.4289	1.32E-08
7	11489	Adam12	mRNA	-2.4904	6.93E-06	8038	ADAM12	mRNA	-0.6334	3.63E-11
8	237360	Adamts14	mRNA	-1.2068	5.56E-06	140766	ADAMTS14	mRNA	-0.9521	0.00726
9	269959	Adamtsl3	mRNA	-0.9462	0.0019262	57188	ADAMTSL3	mRNA	-0.4486	5.99E-05
10	229595	Adamtsl4	mRNA	-1.4049	1.44E-06	54507	ADAMTSL4	mRNA	-0.3734	0.03519
11	110532	Adarb1	mRNA	-1.0209	7.79E-07	104	ADARB1	mRNA	-0.9095	2.13E-06
12	66757	Adat2	mRNA	-0.4773	0.0180696	134637	ADAT2	mRNA	-0.9848	2.54E-08
13	57869	Adck2	mRNA	-1.2226	0.000854	90956	ADCK2	mRNA	-0.3134	0.0451
14	11515	Adcy9	mRNA	-0.9271	0.0032637	115	ADCY9	mRNA	-0.5633	0.00019
15	72141	Adpgk	mRNA	-0.9235	1.26E-08	83440	ADPGK	mRNA	-0.613	1.70E-12
16	100206	Adprhl2	mRNA	-0.8912	0.000134	54936	ADPRHL2	mRNA	-0.4988	4.17E-06
17	11548	Adra1B	mRNA	-5.4561	1.80E-05	147	ADRA1B	mRNA	-1.4488	5.97E-09
18	70292	Afap1	mRNA	-0.7091	0.0013523	60312	AFAP1	mRNA	-0.6034	1.22E-11
19	347722	Agap1	mRNA	-0.5149	0.0075042	116987	AGAP1	mRNA	-0.4858	8.73E-05
20	28169	Agpat3	mRNA	-0.3929	0.0151037	56894	AGPAT3	mRNA	-0.3086	0.00043
21	16475	Ajuba	mRNA	-0.9353	9.96E-06	84962	AJUBA	mRNA	-0.2628	0.00194
22	208211	Alg1	mRNA	-0.6952	5.70E-05	56052	ALG1	mRNA	-0.5994	1.15E-07
23	223774	Alg12	mRNA	-1.1122	1.34E-05	79087	ALG12	mRNA	-0.9117	1.11E-09
24	208624	Alg3	mRNA	-0.6074	0.0013433	10195	ALG3	mRNA	-0.5956	1.07E-09
25	66248	Alg5	mRNA	-0.4155	0.0137319	29880	ALG5	mRNA	-0.416	0.00712
26	320438	Alg6	mRNA	-0.7779	1.60E-05	29929	ALG6	mRNA	-0.6691	0.00036
27	381903	Alg8	mRNA	-0.5417	0.0059811	79053	ALG8	mRNA	-0.503	2.61E-08
28	102580	Alg9	mRNA	-0.3556	0.0397947	79796	ALG9	mRNA	-0.2992	0.00273
29	235633	Als2Cl	mRNA	-0.9377	5.82E-05	259173	ALS2CL	mRNA	-0.4334	0.01857
30	245847	Amdhd2	mRNA	-0.4388	0.0427784	51005	AMDHD2	mRNA	-0.6536	0.00016
31	75723	Amotl1	mRNA	-0.6951	0.0021758	154810	AMOTL1	mRNA	-0.5634	2.21E-10
32	68737	Angel1	mRNA	-1.891	6.86E-09	23357	ANGEL1	mRNA	-0.8272	8.72E-05
33	11736	Ankfy1	mRNA	-0.3258	0.0447652	51479	ANKFY1	mRNA	-0.6262	3.14E-12
34	245886	Ankrd27	mRNA	-1.2218	1.90E-17	84079	ANKRD27	mRNA	-0.2911	0.03311
35	52231	Ankzf1	mRNA	-0.676	0.0040555	55139	ANKZF1	mRNA	-0.4677	0.001
36	102566	Ano10	mRNA	-0.5164	0.0060695	55129	ANO10	mRNA	-0.2441	0.01809
37	11776	Ap3D1	mRNA	-0.409	0.0274231	8943	AP3D1	mRNA	-0.3764	3.78E-06
38	30878	Apln	mRNA	-4.315	6.33E-39	8862	APLN	mRNA	-2.6304	2.17E-05
39	78514	Arhgap10	mRNA	-0.6584	5.56E-06	79658	ARHGAP10	mRNA	-0.6996	9.04E-11
40	214137	Arhgap29	mRNA	-0.9062	2.67E-08	9411	ARHGAP29	mRNA	-0.8853	7.92E-28
41	54126	Arhgef7	mRNA	-0.4911	0.0011676	8874	ARHGEF7	mRNA	-0.4202	2.32E-05
42	320982	Arl4C	mRNA	-0.5246	0.0438794	10123	ARL4C	mRNA	-1.2311	3.34E-22
43	76813	Armc6	mRNA	-0.523	0.0163444	93436	ARMC6	mRNA	-0.8012	2.45E-10
44	70420	Arpin	mRNA	-0.6518	1.19E-06	348110	ARPIN	mRNA	-0.2742	0.03361
45	109689	Arrb1	mRNA	-0.6406	0.022519	408	ARRB1	mRNA	-0.6849	0.00997
46	13196	Asap1	mRNA	-0.9318	6.21E-08	50807	ASAP1	mRNA	-0.4496	1.83E-05
47	142688	Asb13	mRNA	-0.7381	0.000764	79754	ASB13	mRNA	-0.3993	0.0195
48	108888	Atad3A	mRNA	-0.7568	7.56E-06	55210	ATAD3A	mRNA	-1.0039	1.28E-20
49	74772	Atp13A2	mRNA	-0.5666	0.0040149	23400	ATP13A2	mRNA	-0.3909	0.00011
50	11975	Atp6V0A1	mRNA	-0.8188	8.81E-06	535	ATP6V0A1	mRNA	-0.4929	3.08E-05
51	245000	Atr	mRNA	-0.7824	0.000184	545	ATR	mRNA	-0.3913	0.00014
52	26879	B3Galnt1	mRNA	-0.6109	0.0032086	8706	B3GALNT1	mRNA	-0.5174	0.00132

53	26877	B3Galt1	mRNA	-1.2383	8.32E-08	8708	B3GALT1	mRNA	-3.1978	3.77E-07
54	210004	B3Gntl1	mRNA	-1.1181	0.0059056	146712	B3GNTL1	mRNA	-0.8442	0.04189
55	14421	B4Galnt1	mRNA	-0.6391	0.000403	2583	B4GALNT1	mRNA	-0.6811	0.04955
56	53418	B4Galt2	mRNA	-0.7972	0.000149	8704	B4GALT2	mRNA	-0.3883	0.00013
57	56336	B4Galt5	mRNA	-0.7058	0.0026452	9334	B4GALT5	mRNA	-0.7669	1.71E-13
58	23821	Bace1	mRNA	-1.1864	7.78E-08	23621	BACE1	mRNA	-0.2967	0.00035
59	213539	Bag2	mRNA	-0.848	5.55E-08	9532	BAG2	mRNA	-0.6567	1.00E-06
60	29815	Bcar3	mRNA	-0.8475	0.000177	8412	BCAR3	mRNA	-0.7851	8.54E-22
61	12041	Bckdk	mRNA	-0.4079	0.0032889	10295	BCKDK	mRNA	-0.3442	0.00228
62	12043	Bcl2	mRNA	-0.7906	0.000743	596	BCL2	mRNA	-1.6945	2.08E-07
63	66821	Bcs1L	mRNA	-0.6202	0.0031278	617	BCS1L	mRNA	-0.5212	0.00024
64	246229	Bivm	mRNA	-0.6678	1.18E-05	54841	BIVM	mRNA	-0.4301	0.00236
65	104184	Blmh	mRNA	-0.4877	0.0097772	642	BLMH	mRNA	-0.3438	0.00413
66	73230	Bmper	mRNA	-1.04	2.24E-09	168667	BMPER	mRNA	-1.4144	4.41E-32
67	242509	Bnc2	mRNA	-0.7636	0.002138	54796	BNC2	mRNA	-0.5138	4.68E-05
68	51800	Bok	mRNA	-0.9121	4.96E-12	666	BOK	mRNA	-0.3366	0.00049
69	74007	Btbd11	mRNA	-1.7301	5.30E-17	121551	BTBD11	mRNA	-0.8067	6.08E-05
70	12236	Bub1B	mRNA	-0.5102	0.0017501	701	BUB1B	mRNA	-0.3656	0.00078
71	104248	Cabin1	mRNA	-0.4562	0.0430523	23523	CABIN1	mRNA	-0.3083	0.02639
72	69719	Cad	mRNA	-0.7165	6.85E-06	790	CAD	mRNA	-0.7124	2.72E-11
73	55984	Camkk1	mRNA	-2.16	1.24E-07	84254	CAMKK1	mRNA	-0.675	0.0475
74	59035	Carm1	mRNA	-0.5356	0.0030279	10498	CARM1	mRNA	-0.4566	7.06E-06
75	67383	Carnmt1	mRNA	-0.4224	0.0346998	138199	CARNMT1	mRNA	-1.2391	1.24E-16
76	494448	Cbx6	mRNA	-0.4681	0.0072502	23466	CBX6	mRNA	-0.7673	1.57E-11
77	231214	Cc2D2A	mRNA	-1.9754	3.54E-06	57545	CC2D2A	mRNA	-0.306	0.01736
78	214239	Ccdc9B	mRNA	-1.6507	4.79E-13	388115	CCDC9B	mRNA	-0.4746	6.91E-06
79	12443	Ccnd1	mRNA	-2.2636	3.73E-75	595	CCND1	mRNA	-2.5167	#######
80	12521	Cd82	mRNA	-0.4251	0.0042473	3732	CD82	mRNA	-0.579	1.47E-06
81	66953	Cdca7	mRNA	-0.7689	2.24E-07	83879	CDCA7	mRNA	-2.4857	5.53E-48
82	18557	Cdk18	mRNA	-0.8666	0.0067301	5129	CDK18	mRNA	-0.3199	0.03042
83	12571	Cdk6	mRNA	-0.4956	0.0353336	1021	CDK6	mRNA	-0.8605	2.17E-16
84	57810	Cdon	mRNA	-0.5913	0.016789	50937	CDON	mRNA	-0.4924	0.00312
85	12009	Cep131	mRNA	-1.0492	0.0060055	22994	CEP131	mRNA	-0.5827	0.00057
86	16328	Cep250	mRNA	-0.5913	0.0170676	11190	CEP250	mRNA	-0.8664	2.08E-12
87	208518	Cep78	mRNA	-1.3787	1.56E-12	84131	CEP78	mRNA	-0.6323	1.89E-05
88	67260	Cers4	mRNA	-0.824	0.0038068	79603	CERS4	mRNA	-0.4399	0.04574
89	70737	Cgn	mRNA	-1.2491	4.74E-08	57530	CGN	mRNA	-1.7894	2.94E-05
90	208092	Chmp6	mRNA	-0.9472	3.84E-06	79643	CHMP6	mRNA	-0.4465	0.00087
91	58250	Chst11	mRNA	-1.0179	1.26E-09	50515	CHST11	mRNA	-0.3346	0.01492
92	72136	Chst14	mRNA	-1.3378	7.14E-11	113189	CHST14	mRNA	-1.0942	5.66E-28
93	54371	Chst2	mRNA	-1.246	0.0012052	9435	CHST2	mRNA	-1.6043	4.33E-32
94	60322	Chst7	mRNA	-1.1547	3.95E-11	56548	CHST7	mRNA	-0.7212	0.02399
95	76499	Clasp2	mRNA	-0.3198	0.0415553	23122	CLASP2	mRNA	-0.2683	0.02197
96	53609	Clasrp	mRNA	-0.583	0.0016109	11129	CLASRP	mRNA	-0.4148	0.01371
97	76524	Cln6	mRNA	-1.473	1.65E-07	54982	CLN6	mRNA	-0.9041	1.89E-21
98	218335	Clptm1L	mRNA	-0.4123	0.0135285	81037	CLPTM1L	mRNA	-0.508	7.75E-08
99	65945	Clstn1	mRNA	-0.6885	5.95E-05	22883	CLSTN1	mRNA	-0.2862	0.002
100	74157	Cmtr1	mRNA	-0.6005	4.63E-05	23070	CMTR1	mRNA	-0.3174	0.00194
101	12798	Cnn2	mRNA	-0.5128	7.69E-05	1265	CNN2	mRNA	-0.3402	0.00312
102	12799	Cnp	mRNA	-1.1309	0.0215972	1267	CNP	mRNA	-0.3284	0.00135
103	68185	Coa4	mRNA	-0.9741	0.0235477	51287	COA4	mRNA	-0.6305	7.95E-13
104	12837	Col8A1	mRNA	-0.4529	0.0419392	1295	COL8A1	mRNA	-0.4513	1.82E-08
105	66423	Coprs	mRNA	-0.5052	0.0087175	55352	COPRS	mRNA	-0.2846	0.01453
106	230027	Coq3	mRNA	-0.6192	0.0110799	51805	COQ3	mRNA	-0.4563	0.04732
107	23790	Coro1C	mRNA	-0.4838	5.16E-05	23603	CORO1C	mRNA	-0.8232	1.31E-19
108	12892	Cpox	mRNA	-0.4724	0.0019796	1371	CPOX	mRNA	-0.4667	0.00012
109	230872	Crocc	mRNA	-1.3259	0.000596	9696	CROCC	mRNA	-0.5741	0.00449
110	14219	Ctgf	mRNA	-1.4092	0.0145151	1490	CTGF	mRNA	-4.8067	7.47E-10

111	225912	Cyb561A3	mRNA	-1.5227	6.28E-10	220002	CYB561A3	mRNA	-0.2656	0.01607
112	19159	Cyth3	mRNA	-0.4017	0.0126185	9265	CYTH3	mRNA	-0.6812	4.86E-13
113	226539	Dars2	mRNA	-0.98	6.37E-05	55157	DARS2	mRNA	-0.736	1.99E-07
114	13169	Dbnl	mRNA	-0.5797	0.000555	28988	DBNL	mRNA	-0.2555	0.02376
115	66686	Dcbld1	mRNA	-0.7021	0.0039796	285761	DCBLD1	mRNA	-0.9275	1.64E-22
116	69219	Ddah1	mRNA	-0.5785	3.28E-05	23576	DDAH1	mRNA	-0.6368	6.25E-09
117	320209	Ddx11	mRNA	-0.3937	0.0094524	1663	DDX11	mRNA	-0.6258	3.27E-06
118	67848	Ddx55	mRNA	-0.5013	0.005707	57696	DDX55	mRNA	-0.8207	1.42E-11
119	54006	Deaf1	mRNA	-0.7297	0.000504	10522	DEAF1	mRNA	-0.3153	0.00173
120	229541	Dennd4B	mRNA	-0.9516	0.0130266	9909	DENND4B	mRNA	-0.4212	0.00137
121	104418	Dgkz	mRNA	-0.6946	4.97E-05	8525	DGKZ	mRNA	-0.3914	0.00266
122	74754	Dhcr24	mRNA	-1.2458	5.95E-05	1718	DHCR24	mRNA	-0.584	6.11E-17
123	56749	Dhodh	mRNA	-0.9231	8.70E-05	1723	DHODH	mRNA	-0.9275	2.10E-07
124	70451	Dhrs13	mRNA	-0.825	0.000565	147015	DHRS13	mRNA	-0.9679	5.80E-05
125	72831	Dhx30	mRNA	-0.543	0.000447	22907	DHX30	mRNA	-0.6442	2.65E-13
126	71723	Dhx34	mRNA	-1.1865	2.36E-14	9704	DHX34	mRNA	-1.341	1.65E-18
127	71715	Dhx35	mRNA	-0.4315	0.02946	60625	DHX35	mRNA	-0.6961	9.66E-05
128	83945	Dnaja3	mRNA	-0.6963	1.22E-06	9093	DNAJA3	mRNA	-0.2713	0.00735
129	76088	Dock8	mRNA	-1.3272	0.000157	81704	DOCK8	mRNA	-0.7659	0.00063
130	105445	Dock9	mRNA	-0.6083	0.0162869	23348	DOCK9	mRNA	-0.3862	0.00102
131	227697	Dolk	mRNA	-0.7	0.0027283	22845	DOLK	mRNA	-0.6432	5.75E-08
132	116905	Dph1	mRNA	-1.5111	6.53E-15	1801	DPH1	mRNA	-0.4843	0.00029
133	67228	Dph7	mRNA	-1.3598	6.52E-07	92715	DPH7	mRNA	-0.3795	0.00578
134	75221	Dpp3	mRNA	-0.3361	0.0395426	10072	DPP3	mRNA	-0.4145	2.90E-05
135	319901	Dsel	mRNA	-0.7832	6.72E-07	92126	DSEL	mRNA	-1.8362	3.08E-35
136	209200	Dtx3L	mRNA	-1.5663	6.49E-10	151636	DTX3L	mRNA	-0.2614	0.03039
137	56405	Dusp14	mRNA	-0.4789	0.0046434	11072	DUSP14	mRNA	-0.7398	4.03E-14
138	235584	Dusp7	mRNA	-0.6619	0.0035942	1849	DUSP7	mRNA	-0.3367	0.00416
139	56455	Dynll1	mRNA	-0.2732	0.0347595	8655	DYNLL1	mRNA	-0.3038	0.00788
140	67417	Ears2	mRNA	-0.7593	0.0018882	124454	EARS2	mRNA	-0.5048	0.0001
141	13631	Eef2K	mRNA	-1.4962	3.77E-09	29904	EEF2K	mRNA	-0.447	0.00036
142	70511	Eef2Kmt	mRNA	-1.3024	2.90E-08	196483	EEF2KMT	mRNA	-0.6037	2.28E-07
143	67484	Eepd1	mRNA	-1.4206	0.0022888	80820	EEPD1	mRNA	-2.0313	5.26E-09
144	230648	Efcab14	mRNA	-0.3039	0.0367253	9813	EFCAB14	mRNA	-0.276	0.00185
145	112407	Egln3	mRNA	-1.3024	1.26E-08	112399	EGLN3	mRNA	-2.2819	1.05E-20
146	57440	Ehd3	mRNA	-1.565	0.0232236	30845	EHD3	mRNA	-0.5766	0.00624
147	98878	Ehd4	mRNA	-0.457	0.000686	30844	EHD4	mRNA	-0.289	0.01607
148	13663	Ei24	mRNA	-0.7983	2.00E-09	9538	EI24	mRNA	-0.2692	0.00491
149	27103	Eif2Ak4	mRNA	-0.6199	0.000463	440275	EIF2AK4	mRNA	-0.2884	0.00347
150	224045	Eif2B5	mRNA	-0.3118	0.0419217	8893	EIF2B5	mRNA	-0.3108	0.00274
151	208691	Eif5A2	mRNA	-0.401	0.0083016	56648	EIF5A2	mRNA	-0.4369	0.0157
152	170439	Elovl6	mRNA	-0.6895	0.0260577	79071	ELOVL6	mRNA	-0.8254	3.34E-20
153	230866	Emc1	mRNA	-0.4564	0.0054488	23065	EMC1	mRNA	-0.3728	8.41E-05
154	78798	Eml4	mRNA	-0.675	5.68E-07	27436	EML4	mRNA	-0.5294	3.62E-07
155	13803	Enc1	mRNA	-1.2626	6.04E-07	8507	ENC1	mRNA	-1.992	8.67E-81
156	71946	Endod1	mRNA	-1.5187	5.67E-11	23052	ENDOD1	mRNA	-1.1301	1.67E-31
157	67870	Enoph1	mRNA	-0.4207	0.0269942	58478	ENOPH1	mRNA	-0.234	0.03127
158	209224	Enox2	mRNA	-0.6548	0.0153426	10495	ENOX2	mRNA	-0.928	3.63E-09
159	67464	Entpd4	mRNA	-1.6292	0.0022888	9583	ENTPD4	mRNA	-0.9802	7.45E-29
160	101351	Eogt	mRNA	-1.2478	3.51E-11	285203	EOGT	mRNA	-0.6664	3.87E-10
161	13822	Epb41L2	mRNA	-0.4393	0.0013169	2037	EPB41L2	mRNA	-0.451	1.67E-08
162	13844	Ephb2	mRNA	-0.6005	0.0248968	2048	EPHB2	mRNA	-0.7406	1.26E-10
163	13866	Erbb2	mRNA	-0.5022	0.0092858	2064	ERBB2	mRNA	-0.3419	0.00155
164	76251	Ercc6L2	mRNA	-0.5136	0.0015881	375748	ERCC6L2	mRNA	-0.3017	0.01392
165	226144	Erlin1	mRNA	-0.6064	2.19E-06	10613	ERLIN1	mRNA	-0.6343	4.52E-14
166	244373	Erlin2	mRNA	-0.5661	0.000877	11160	ERLIN2	mRNA	-0.4442	3.38E-07
167	14009	Etv1	mRNA	-1.2418	0.0260262	2115	ETV1	mRNA	-1.1501	2.49E-07
168	107371	Exoc6	mRNA	-0.8707	3.78E-06	54536	EXOC6	mRNA	-0.4455	0.0022

169	66446	Exosc7	mRNA	-0.8511	1.06E-05	23016	EXOSC7	mRNA	-0.2992	0.03116
170	14043	Ext2	mRNA	-0.6189	0.000107	2132	EXT2	mRNA	-0.1861	0.04483
171	71885	Faap100	mRNA	-1.0822	5.64E-06	80233	FAAP100	mRNA	-0.5492	5.83E-06
172	329739	Fam102B	mRNA	-0.9128	4.49E-06	284611	FAM102B	mRNA	-0.4295	0.01688
173	242297	Fam110B	mRNA	-1.3364	0.000266	90362	FAM110B	mRNA	-0.5285	0.00424
174	218236	Fam120A	mRNA	-0.2696	0.0428921	23196	FAM120A	mRNA	-0.2848	0.0007
175	78755	Fam122B	mRNA	-0.5968	0.0092827	159090	FAM122B	mRNA	-0.4323	0.0003
176	269233	Fam171A1	mRNA	-0.8228	3.16E-06	221061	FAM171A1	mRNA	-0.7766	1.91E-12
177	215015	Fam20B	mRNA	-0.4499	0.0022434	9917	FAM20B	mRNA	-0.3989	6.40E-05
178	80752	Fam20C	mRNA	-1.0169	3.35E-06	56975	FAM20C	mRNA	-0.3344	0.01288
179	106581	Fam234A	mRNA	-0.3766	0.0190468	83986	FAM234A	mRNA	-0.2168	0.04504
180	74525	Fam234B	mRNA	-1.2494	0.0015731	57613	FAM234B	mRNA	-0.8161	0.0009
181	27999	Fam3C	mRNA	-1.1758	4.45E-12	10447	FAM3C	mRNA	-0.5228	1.07E-08
182	77938	Fam53B	mRNA	-0.6613	0.0471552	9679	FAM53B	mRNA	-0.6519	0.00347
183	116972	Fam57A	mRNA	-1.0021	2.20E-08	79850	FAM57A	mRNA	-1.5175	6.54E-57
184	67266	Fam69A	mRNA	-0.4544	0.0425864	388650	FAM69A	mRNA	-0.3108	0.04497
185	105732	Fam83H	mRNA	-0.6378	0.000481	286077	FAM83H	mRNA	-0.3259	0.00753
186	14087	Fanca	mRNA	-0.4779	0.016145	2175	FANCA	mRNA	-0.4207	0.00066
187	14102	Fas	mRNA	-1.5964	8.00E-10	355	FAS	mRNA	-0.5252	2.20E-06
188	14107	Fat1	mRNA	-0.7	0.0304144	2195	FAT1	mRNA	-1.0387	2.75E-24
189	231670	Fbxo21	mRNA	-0.5611	0.000215	23014	FBXO21	mRNA	-0.3057	0.00525
190	207278	Fchsd2	mRNA	-0.5132	0.0238832	9873	FCHSD2	mRNA	-0.45	0.00021
191	14229	Fkbp5	mRNA	-0.9893	9.02E-10	2289	FKBP5	mRNA	-0.7681	3.43E-12
192	246179	Fktn	mRNA	-0.5891	0.000134	2218	FKTN	mRNA	-0.3099	0.00275
193	68794	Flnc	mRNA	-0.6698	0.0106849	2318	FLNC	mRNA	-1.023	4.44E-18
194	226844	Flvcr1	mRNA	-0.4204	0.0347352	28982	FLVCR1	mRNA	-0.397	0.00691
195	14260	Fmn1	mRNA	-0.9567	2.38E-09	342184	FMN1	mRNA	-1.2443	2.25E-11
196	239554	Foxred2	mRNA	-1.0697	1.11E-09	80020	FOXRED2	mRNA	-1.1551	6.80E-22
197	319710	Frmd6	mRNA	-0.5418	0.0264045	122786	FRMD6	mRNA	-1.8029	1.18E-74
198	14086	Fscn1	mRNA	-0.5551	2.34E-05	6624	FSCN1	mRNA	-0.827	0.00048
199	17281	Fyco1	mRNA	-0.845	0.0011553	79443	FYCO1	mRNA	-0.392	0.0004
200	57265	Fzd2	mRNA	-0.6722	2.44E-05	2535	FZD2	mRNA	-0.701	1.34E-11
201	17873	Gadd45B	mRNA	-1.0956	2.62E-12	4616	GADD45B	mRNA	-1.9595	7.84E-74
202	171212	Galnt10	mRNA	-0.6877	1.21E-05	55568	GALNT10	mRNA	-0.7581	5.51E-19
203	108148	Galnt2	mRNA	-0.628	2.46E-05	2590	GALNT2	mRNA	-0.2793	0.00408
204	14456	Gas6	mRNA	-0.5693	0.0310109	2621	GAS6	mRNA	-0.3427	9.15E-05
205	14461	Gata2	mRNA	-0.9683	0.0012858	2624	GATA2	mRNA	-1.6492	0.00208
206	384281	Gatc	mRNA	-0.7512	7.84E-05	283459	GATC	mRNA	-0.2805	0.01872
207	213350	Gatd1	mRNA	-1.5043	2.50E-06	347862	GATD1	mRNA	-0.4322	9.83E-05
208	107338	Gbf1	mRNA	-0.5998	0.0013764	8729	GBF1	mRNA	-0.4605	9.66E-07
209	276919	Gemin4	mRNA	-1.1679	3.05E-06	50628	GEMIN4	mRNA	-1.6906	9.43E-58
210	216766	Gemin5	mRNA	-0.5245	1.27E-05	25929	GEMIN5	mRNA	-1.5695	2.17E-42
211	69080	Gmppa	mRNA	-0.4956	0.0066393	29926	GMPPA	mRNA	-0.416	0.00036
212	331026	Gmppb	mRNA	-0.7995	0.0335588	29925	GMPPB	mRNA	-0.6078	0.00016
213	14673	Gna12	mRNA	-0.4184	0.0488905	2768	GNA12	mRNA	-0.2397	0.02216
214	14696	Gnb4	mRNA	-0.6117	0.0015049	59345	GNB4	mRNA	-0.335	0.00255
215	432486	Gnptab	mRNA	-1.4147	8.62E-20	79158	GNPTAB	mRNA	-0.353	0.00098
216	105348	Golm1	mRNA	-0.4269	0.0021193	51280	GOLM1	mRNA	-0.2876	0.00071
217	56494	Gosr2	mRNA	-0.3781	0.0059448	9570	GOSR2	mRNA	-0.2357	0.02113
218	14732	Gpam	mRNA	-1.3078	9.09E-18	57678	GPAM	mRNA	-0.3818	0.00042
219	381413	Gpr176	mRNA	-2.158	1.13E-09	11245	GPR176	mRNA	-1.043	7.32E-39
220	223752	Gramd4	mRNA	-0.8926	3.41E-06	23151	GRAMD4	mRNA	-0.9196	0.00016
221	381157	Greb1L	mRNA	-0.9926	9.01E-05	80000	GREB1L	mRNA	-1.3223	2.73E-08
222	56541	Habp4	mRNA	-0.6225	0.0020798	22927	HABP4	mRNA	-0.6942	4.38E-07
223	57874	Hacd3	mRNA	-0.4681	0.000106	51495	HACD3	mRNA	-0.4517	1.96E-08
224	234549	Heatr3	mRNA	-0.3213	0.0432012	55027	HEATR3	mRNA	-1.3063	1.64E-22
225	59053	Hgh1	mRNA	-0.5846	0.00064	51236	HGH1	mRNA	-0.4391	0.0003
226	52120	Hgsnat	mRNA	-0.7982	6.78E-10	138050	HGSNAT	mRNA	-0.3211	0.00518

227	16656	Hivep3	mRNA	-2.5246	1.64E-13	59269	HIVEP3	mRNA	-0.8963	0.01386
228	70823	Hmgxb4	mRNA	-0.4638	0.0056912	10042	HMGXB4	mRNA	-0.4647	1.39E-05
229	11991	Hnrnpd	mRNA	-0.5227	0.00037	3184	HNRNPD	mRNA	-0.7613	2.38E-19
230	50926	Hnrnpdl	mRNA	-0.356	0.0017486	9987	HNRNPDL	mRNA	-0.2783	0.00124
231	74326	Hnrnpr	mRNA	-0.3676	0.0048966	10236	HNRNPR	mRNA	-0.4291	1.36E-08
232	23908	Hs2St1	mRNA	-0.4603	0.000333	9653	HS2ST1	mRNA	-0.5835	3.03E-07
233	15194	Htt	mRNA	-0.5449	0.0092834	3064	HTT	mRNA	-0.5466	1.82E-07
234	320802	Ifitm10	mRNA	-2.338	5.05E-07	402778	IFITM10	mRNA	-0.5229	0.01353
235	67454	Ikbip	mRNA	-0.4429	0.0046979	121457	IKBIP	mRNA	-0.2178	0.0115
236	16180	Il1Rap	mRNA	-0.446	0.0207687	3556	IL1RAP	mRNA	-0.389	0.00068
237	1E+08	Ildr2	mRNA	-0.8746	6.79E-05	387597	ILDR2	mRNA	-1.8374	0.01583
238	70422	Ints2	mRNA	-0.7292	2.39E-06	57508	INTS2	mRNA	-0.3942	0.01322
239	229543	Ints3	mRNA	-0.6559	1.44E-05	65123	INTS3	mRNA	-0.541	1.60E-06
240	320727	Ipo8	mRNA	-0.5345	0.000341	10526	IPO8	mRNA	-0.4124	4.60E-05
241	232227	Iqsec1	mRNA	-1.0059	1.10E-05	9922	IQSEC1	mRNA	-0.7486	6.53E-09
242	66307	Isoc1	mRNA	-0.7507	0.000469	51015	ISOC1	mRNA	-0.3375	0.04689
243	16400	Itga3	mRNA	-0.5198	7.31E-05	3675	ITGA3	mRNA	-0.3533	0.00146
244	16403	Itga6	mRNA	-1.7062	4.03E-37	3655	ITGA6	mRNA	-0.3226	0.00214
245	16419	Itgb5	mRNA	-0.5931	0.0027781	3693	ITGB5	mRNA	-0.5372	4.10E-09
246	16469	Jrk	mRNA	-1.2359	8.14E-10	8629	JRK	mRNA	-0.6989	1.59E-05
247	14534	Kat2A	mRNA	-0.378	0.0054831	2648	KAT2A	mRNA	-0.4705	0.00037
248	16526	Kcnk2	mRNA	-1.4049	9.41E-08	3776	KCNK2	mRNA	-0.5224	0.00164
249	68304	Kdelc2	mRNA	-1.0772	2.18E-12	143888	KDELC2	mRNA	-0.7162	4.91E-18
250	218214	Kdm1B	mRNA	-0.7571	7.20E-05	221656	KDM1B	mRNA	-0.7498	1.24E-07
251	30841	Kdm2B	mRNA	-0.5847	0.0021611	84678	KDM2B	mRNA	-0.6488	3.74E-07
252	16553	Kif13A	mRNA	-0.6197	0.000824	63971	KIF13A	mRNA	-0.2048	0.0288
253	246293	Klhl8	mRNA	-0.9768	0.0203002	57563	KLHL8	mRNA	-0.7964	2.56E-05
254	214669	L3Mbtl2	mRNA	-0.3285	0.0359267	83746	L3MBTL2	mRNA	-0.7469	6.54E-11
255	212442	Lactb2	mRNA	-0.6517	9.33E-05	51110	LACTB2	mRNA	-0.2904	0.03327
256	226519	Lamc1	mRNA	-0.734	0.000159	3915	LAMC1	mRNA	-0.2807	0.00137
257	114128	Laptm4B	mRNA	-0.5087	0.000443	55353	LAPTM4B	mRNA	-0.1992	0.02386
258	102436	Lars2	mRNA	-0.7703	0.0028152	23395	LARS2	mRNA	-0.8144	4.78E-13
259	244864	Layn	mRNA	-1.2049	3.62E-09	143903	LAYN	mRNA	-0.7989	4.75E-10
260	241576	Ldlrad3	mRNA	-0.6004	0.0012218	143458	LDLRAD3	mRNA	-1.1484	1.13E-17
261	16842	Lef1	mRNA	-0.7281	0.0018039	51176	LEF1	mRNA	-0.9672	0.00142
262	70361	Lman1	mRNA	-0.4528	0.000332	3998	LMAN1	mRNA	-0.1879	0.01703
263	214895	Lman2L	mRNA	-0.6878	0.0051466	81562	LMAN2L	mRNA	-0.737	2.09E-09
264	76483	Lmf1	mRNA	-0.3781	0.042435	64788	LMF1	mRNA	-0.9765	0.00077
265	16907	Lmnb2	mRNA	-0.8465	5.40E-06	84823	LMNB2	mRNA	-1.119	1.47E-37
266	380928	Lmo7	mRNA	-1.1097	9.75E-07	4008	LMO7	mRNA	-0.8538	4.16E-16
267	16949	Loxl1	mRNA	-0.93	0.000408	4016	LOXL1	mRNA	-0.4703	4.58E-06
268	210992	Lpcat1	mRNA	-1.3765	7.44E-22	79888	LPCAT1	mRNA	-1.6385	1.35E-49
269	270084	Lpcat2	mRNA	-0.6531	0.000339	54947	LPCAT2	mRNA	-0.4144	0.00011
270	228357	Lrp4	mRNA	-0.7818	0.0122169	4038	LRP4	mRNA	-1.0166	0.0199
271	216011	Lrrc20	mRNA	-0.651	0.0015123	55222	LRRC20	mRNA	-0.5046	0.01875
272	433926	Lrrc8B	mRNA	-0.7899	8.10E-05	23507	LRRC8B	mRNA	-1.8368	7.46E-12
273	100604	Lrrc8C	mRNA	-0.5507	0.0123353	84230	LRRC8C	mRNA	-0.4313	0.00236
274	72267	Lrrc8E	mRNA	-0.6162	0.0204037	80131	LRRC8E	mRNA	-0.9191	3.44E-08
275	78651	Lsm6	mRNA	-0.3485	0.0127814	11157	LSM6	mRNA	-0.6793	5.70E-05
276	17096	Lyn	mRNA	-1.3811	3.22E-12	4067	LYN	mRNA	-0.442	7.35E-05
277	270118	Maml2	mRNA	-1.2539	0.000307	84441	MAML2	mRNA	-0.2232	0.03466
278	17158	Man2A1	mRNA	-0.9676	1.34E-07	4124	MAN2A1	mRNA	-0.3999	1.84E-06
279	242362	Manea	mRNA	-0.4223	0.0159629	79694	MANEA	mRNA	-0.4363	0.0001
280	26407	Map3K4	mRNA	-1.7292	1.45E-22	4216	MAP3K4	mRNA	-0.4793	4.49E-06
281	227743	Mapkap1	mRNA	-0.3862	0.0337422	79109	MAPKAP1	mRNA	-0.2386	0.00353
282	277010	Marveld1	mRNA	-0.7264	5.61E-05	83742	MARVELD	mRNA	-0.3837	2.59E-05
283	239796	Mb21D2	mRNA	-0.7232	0.000919	151963	MB21D2	mRNA	-1.346	4.43E-21
284	72852	Mblac2	mRNA	-1.2777	0.0180119	153364	MBLAC2	mRNA	-0.9337	5.43E-07

285	218121	Mboat1	mRNA	-0.6858	0.000204	154141	MBOAT1	mRNA	-0.5769	0.04951
286	67216	Mboat2	mRNA	-0.7603	0.000447	129642	MBOAT2	mRNA	-0.351	7.34E-05
287	78038	Mccc2	mRNA	-0.7436	0.000104	64087	MCCC2	mRNA	-0.2688	0.00418
288	240087	Mdc1	mRNA	-0.6287	0.0015947	9656	MDC1	mRNA	-0.6639	2.99E-08
289	17260	Mef2C	mRNA	-1.6976	1.78E-08	4208	MEF2C	mRNA	-1.5536	2.66E-09
290	17536	Meis2	mRNA	-0.7626	0.000243	4212	MEIS2	mRNA	-0.5179	0.00011
291	17295	Met	mRNA	-1.1015	1.06E-08	4233	MET	mRNA	-0.6229	1.01E-17
292	17299	Mettl1	mRNA	-0.6845	0.0047073	4234	METTL1	mRNA	-1.1593	5.87E-14
293	71306	Mfap3L	mRNA	-1.467	0.000921	9848	MFAP3L	mRNA	-0.7238	4.75E-10
294	170731	Mfn2	mRNA	-0.3134	0.0457242	9927	MFN2	mRNA	-0.4376	1.21E-07
295	217664	Mgat2	mRNA	-0.4129	0.0404085	4247	MGAT2	mRNA	-0.3592	0.00096
296	107895	Mgat5	mRNA	-0.468	0.0311208	4249	MGAT5	mRNA	-1.5493	1.65E-26
297	320878	Mical2	mRNA	-2.2585	4.24E-36	9645	MICAL2	mRNA	-0.3111	0.0034
298	17342	Mitf	mRNA	-1.3205	0.0051092	4286	MITF	mRNA	-0.5724	8.12E-05
299	109154	Mlec	mRNA	-0.3828	0.0059042	9761	MLEC	mRNA	-0.9433	6.00E-35
300	67096	Mmachc	mRNA	-0.5748	0.0370676	25974	MMACHC	mRNA	-0.9738	8.03E-05
301	57377	Mogs	mRNA	-0.6921	2.84E-06	7841	MOGS	mRNA	-0.8085	1.14E-14
302	17454	Mov10	mRNA	-0.7868	9.26E-05	4343	MOV10	mRNA	-0.3863	0.00031
303	320183	Msrb3	mRNA	-0.6214	2.85E-05	253827	MSRB3	mRNA	-0.3227	0.00081
304	229524	Msto1	mRNA	-0.6167	3.12E-05	55154	MSTO1	mRNA	-0.6973	2.56E-10
305	108156	Mthfd1	mRNA	-0.2834	0.0424835	4522	MTHFD1	mRNA	-0.3565	0.0011
306	238505	Mtr	mRNA	-0.6885	1.13E-05	4548	MTR	mRNA	-0.5372	2.35E-07
307	210009	Mtrr	mRNA	-0.4439	0.0483424	4552	MTRR	mRNA	-0.2834	0.01116
308	102103	Mtus1	mRNA	-2.276	0.0157803	57509	MTUS1	mRNA	-1.5341	0.00049
309	72543	Mvb12B	mRNA	-0.735	3.82E-05	89853	MVB12B	mRNA	-0.4588	0.02949
310	17865	Mybl2	mRNA	-0.5346	0.000737	4605	MYBL2	mRNA	-0.4524	0.00107
311	71602	Myo1E	mRNA	-1.0371	0.000351	4643	MYO1E	mRNA	-0.5562	2.03E-10
312	67991	Nacc2	mRNA	-1.1162	3.80E-07	138151	NACC2	mRNA	-0.3352	0.00149
313	56174	Nagk	mRNA	-0.7369	0.0303742	55577	NAGK	mRNA	-0.4061	0.00195
314	244141	Nars2	mRNA	-0.8947	4.74E-06	79731	NARS2	mRNA	-0.7088	3.12E-08
315	98956	Nat10	mRNA	-0.7629	6.89E-09	55226	NAT10	mRNA	-1.006	1.19E-34
316	78286	Nav2	mRNA	-1.2597	3.07E-05	89797	NAV2	mRNA	-0.8588	6.40E-12
317	235627	Nbeal2	mRNA	-1.2985	0.000187	23218	NBEAL2	mRNA	-1.168	8.16E-12
318	17965	Nbl1	mRNA	-1.1791	6.64E-08	4681	NBL1	mRNA	-0.5425	0.00431
319	320024	Nceh1	mRNA	-0.6883	0.0046434	57552	NCEH1	mRNA	-0.5802	1.98E-12
320	80987	Nckipsd	mRNA	-0.6357	0.0113613	51517	NCKIPSD	mRNA	-0.3833	0.0016
321	103425	Ncln	mRNA	-0.3568	0.036314	56926	NCLN	mRNA	-0.9285	2.93E-17
322	59126	Nek6	mRNA	-1.3659	1.22E-07	10783	NEK6	mRNA	-0.2798	0.00158
323	24116	Nelfa	mRNA	-0.3531	0.0444396	7469	NELFA	mRNA	-0.3631	0.02222
324	50877	Neu3	mRNA	-1.1357	0.0028389	10825	NEU3	mRNA	-0.6392	0.00671
325	68810	Nexn	mRNA	-0.9056	0.003206	91624	NEXN	mRNA	-2.2116	3.70E-90
326	18032	Nfix	mRNA	-0.6072	0.0035999	4784	NFIX	mRNA	-0.2513	0.02808
327	59007	Ngly1	mRNA	-0.5635	0.041822	55768	NGLY1	mRNA	-0.346	0.00928
328	245269	Nim1K	mRNA	-1.0835	0.000445	167359	NIM1K	mRNA	-1.5009	0.01518
329	217011	Nle1	mRNA	-0.6541	0.000204	54475	NLE1	mRNA	-0.4468	0.00071
330	68979	Nol11	mRNA	-0.3527	0.0046679	25926	NOL11	mRNA	-0.5764	2.28E-10
331	18128	Notch1	mRNA	-0.6373	0.0324447	4851	NOTCH1	mRNA	-1.0431	1.91E-09
332	17168	Nprl3	mRNA	-0.7844	0.0010536	8131	NPRL3	mRNA	-0.4433	0.00081
333	11819	Nr2F2	mRNA	-0.4108	0.0066406	7026	NR2F2	mRNA	-1.3015	4.66E-50
334	68404	Nrn1	mRNA	-0.6589	0.000556	51299	NRN1	mRNA	-5.0684	0.00541
335	18187	Nrp2	mRNA	-0.8408	0.0079269	8828	NRP2	mRNA	-0.8113	0.00065
336	66647	Nsmce3	mRNA	-0.4221	0.0241196	56160	NSMCE3	mRNA	-0.5187	8.18E-05
337	56876	Nsmf	mRNA	-0.6429	0.000385	26012	NSMF	mRNA	-0.3489	0.00357
338	76952	Nt5C2	mRNA	-0.6071	1.06E-07	22978	NT5C2	mRNA	-0.4877	3.56E-08
339	18230	Nxn	mRNA	-0.7059	2.45E-07	64359	NXN	mRNA	-0.6326	0.02873
340	102644	Oaf	mRNA	-0.7945	3.72E-07	220323	OAF	mRNA	-0.3322	0.00897
341	70155	Ogfrl1	mRNA	-1.1022	0.00055	79627	OGFRL1	mRNA	-0.5742	5.93E-09
342	67013	Oma1	mRNA	-1.595	1.54E-05	115209	OMA1	mRNA	-0.7443	0.00026

343	269717	Orai2	mRNA	-0.6406	0.04159	80228	ORAI2	mRNA	-0.3826	0.00414
344	14628	Ostm1	mRNA	-0.5547	0.0042008	28962	OSTM1	mRNA	-0.394	8.15E-05
345	94045	P2Rx5	mRNA	-1.3391	0.0345537	5026	P2RX5	mRNA	-0.6076	8.62E-05
346	18442	P2Ry2	mRNA	-1.4059	0.0051466	5029	P2RY2	mRNA	-0.68	5.08E-07
347	66180	P3H4	mRNA	-0.4127	0.0202666	10609	P3H4	mRNA	-0.6109	8.93E-06
348	18452	P4Ha2	mRNA	-0.3505	0.0179659	8974	P4HA2	mRNA	-0.5806	4.28E-13
349	27355	Pald1	mRNA	-0.4008	0.0483424	27143	PALD1	mRNA	-0.814	3.91E-05
350	242481	Palm2	mRNA	-1.6549	0.000919	114299	PALM2	mRNA	-0.7521	0.02018
351	231474	Paqr3	mRNA	-0.756	0.002881	152559	PAQR3	mRNA	-0.5747	2.62E-06
352	76498	Paqr4	mRNA	-1.2887	1.52E-06	124222	PAQR4	mRNA	-0.6619	4.46E-09
353	74108	Parn	mRNA	-0.7723	7.94E-07	5073	PARN	mRNA	-0.239	0.03928
354	11545	Parp1	mRNA	-0.3427	0.008829	142	PARP1	mRNA	-0.7553	3.72E-18
355	243771	Parp12	mRNA	-0.7959	0.0220518	64761	PARP12	mRNA	-0.5547	1.60E-10
356	547253	Parp14	mRNA	-3.1547	0.0072502	54625	PARP14	mRNA	-0.42	0.00048
357	80285	Parp9	mRNA	-1.4375	1.05E-11	83666	PARP9	mRNA	-0.2975	0.01244
358	73173	Pcdh18	mRNA	-0.5035	0.0369947	54510	PCDH18	mRNA	-1.5745	1.80E-05
359	93706	Pcdhgc3	mRNA	-0.6431	0.0071462	5098	PCDHGC3	mRNA	-1.0793	4.21E-14
360	76477	Pcolce2	mRNA	-0.5847	0.000343	26577	PCOLCE2	mRNA	-0.5843	0.00634
361	18572	Pdcd11	mRNA	-0.3915	0.000972	22984	PDCD11	mRNA	-0.8573	1.94E-17
362	68023	Pdf	mRNA	-0.9055	2.99E-07	64146	PDF	mRNA	-0.3751	0.04856
363	12304	Pdia4	mRNA	-0.3605	0.0071915	9601	PDIA4	mRNA	-0.8006	8.32E-28
364	72599	Pdia5	mRNA	-0.4218	0.0100481	10954	PDIA5	mRNA	-0.3733	0.00059
365	228026	Pdk1	mRNA	-0.9033	0.000186	5163	PDK1	mRNA	-1.0847	1.55E-20
366	56376	Pdlim5	mRNA	-0.8191	2.42E-06	10611	PDLIM5	mRNA	-0.6995	1.04E-15
367	56075	Pdss1	mRNA	-0.4602	0.0230801	23590	PDSS1	mRNA	-0.7975	2.39E-07
368	73182	Pear1	mRNA	-0.997	1.69E-08	375033	PEAR1	mRNA	-0.7482	6.97E-05
369	240518	Peli3	mRNA	-1.412	0.0270793	246330	PELI3	mRNA	-1.3485	2.16E-09
370	170768	Pfkfb3	mRNA	-0.8982	1.72E-05	5209	PFKFB3	mRNA	-1.9237	3.20E-55
371	66681	Pgm1	mRNA	-0.6897	1.52E-07	5236	PGM1	mRNA	-0.3222	0.0047
372	18685	Phtf1	mRNA	-0.5014	0.0071754	10745	PHTF1	mRNA	-0.5071	3.18E-05
373	14755	Pigq	mRNA	-0.4097	0.0035119	9091	PIGQ	mRNA	-0.4673	5.88E-05
374	70325	Pigw	mRNA	-0.3627	0.0329858	284098	PIGW	mRNA	-0.8641	4.83E-09
375	18709	Pik3R2	mRNA	-0.3612	0.028528	5296	PIK3R2	mRNA	-0.5669	7.87E-08
376	72400	Pinx1	mRNA	-0.9467	4.11E-06	54984	PINX1	mRNA	-0.5909	0.00015
377	263803	Pkn3	mRNA	-0.7966	0.029413	29941	PKN3	mRNA	-0.4366	0.01258
378	234779	Plcg2	mRNA	-0.5503	0.0135529	5336	PLCG2	mRNA	-1.2104	0.00011
379	211945	Plekhh1	mRNA	-0.7523	0.0274963	57475	PLEKHH1	mRNA	-1.1334	1.00E-05
380	18822	Plod1	mRNA	-0.5766	0.000146	5351	PLOD1	mRNA	-0.2729	0.00334
381	71910	Plpp5	mRNA	-0.624	0.000556	84513	PLPP5	mRNA	-0.5093	3.80E-05
382	235044	Plppr2	mRNA	-0.6293	0.0056804	64748	PLPPR2	mRNA	-0.6899	8.00E-08
383	18844	Plxna1	mRNA	-0.8777	2.23E-06	5361	PLXNA1	mRNA	-0.745	2.71E-10
384	18845	Plxna2	mRNA	-0.6649	0.0033848	5362	PLXNA2	mRNA	-0.4021	0.02618
385	67784	Plxnd1	mRNA	-0.9643	5.20E-06	23129	PLXND1	mRNA	-0.6729	1.74E-09
386	65112	Pmepa1	mRNA	-0.8414	1.06E-05	56937	PMEPA1	mRNA	-0.2399	0.00279
387	227099	Pms1	mRNA	-1.0948	4.70E-08	5378	PMS1	mRNA	-0.8004	8.58E-11
388	71701	Pnpt1	mRNA	-0.3534	0.0348395	87178	PNPT1	mRNA	-0.6819	2.49E-12
389	27205	Podxl	mRNA	-1.2397	6.42E-14	5420	PODXL	mRNA	-0.9744	1.62E-15
390	140484	Pofut1	mRNA	-0.7939	6.78E-06	23509	POFUT1	mRNA	-0.6463	1.25E-13
391	20017	Polr1B	mRNA	-0.8708	3.25E-11	84172	POLR1B	mRNA	-0.8701	4.63E-16
392	26939	Polr3E	mRNA	-0.5205	0.0010751	55718	POLR3E	mRNA	-0.6097	7.98E-07
393	67486	Polr3G	mRNA	-0.6686	0.000218	10622	POLR3G	mRNA	-1.3838	7.43E-14
394	68273	Pomgnt1	mRNA	-0.4603	0.0149839	55624	POMGNT1	mRNA	-0.387	2.51E-05
395	170826	Ppargc1B	mRNA	-3.2195	8.69E-14	133522	PPARGC1B	mRNA	-2.5724	0.00021
396	327655	Ppip5K1	mRNA	-0.6301	0.0178986	9677	PPIP5K1	mRNA	-0.3024	0.04435
397	68606	Ppm1F	mRNA	-0.4984	2.46E-05	9647	PPM1F	mRNA	-0.4533	0.00027
398	235542	Ppp2R3A	mRNA	-0.4451	0.0185949	5523	PPP2R3A	mRNA	-0.2755	0.00545
399	50907	Preb	mRNA	-0.4477	0.0028003	10113	PREB	mRNA	-0.3071	0.00878
400	19090	Prkdc	mRNA	-0.6472	1.42E-05	5591	PRKDC	mRNA	-0.5534	3.17E-08

401	27374	Prmt5	mRNA	-0.4719	0.0085841	10419	PRMT5	mRNA	-0.3062	0.00021
402	214572	Prmt7	mRNA	-0.9414	0.0010514	54496	PRMT7	mRNA	-0.3797	0.01003
403	110639	Prps2	mRNA	-0.603	1.26E-07	5634	PRPS2	mRNA	-0.7949	1.42E-19
404	76453	Prss23	mRNA	-0.6591	0.001544	11098	PRSS23	mRNA	-1.1995	7.45E-66
405	19165	Psen2	mRNA	-1.6531	0.0033503	5664	PSEN2	mRNA	-0.4313	0.01399
406	66645	Pspc1	mRNA	-0.5114	0.0053876	55269	PSPC1	mRNA	-0.3072	0.00201
407	19221	Ptgfrn	mRNA	-0.675	0.0073141	5738	PTGFRN	mRNA	-0.7237	1.18E-12
408	19250	Ptpn14	mRNA	-0.775	0.000141	5784	PTPN14	mRNA	-0.4411	2.07E-05
409	78697	Pus7	mRNA	-0.7935	4.97E-06	54517	PUS7	mRNA	-1.7704	9.73E-45
410	78895	Pus7L	mRNA	-1.4315	1.47E-09	83448	PUS7L	mRNA	-0.9345	1.48E-18
411	69051	Pycr2	mRNA	-0.5257	0.0186233	29920	PYCR2	mRNA	-0.2644	0.02524
412	104009	Qsox1	mRNA	-0.4614	0.0034605	5768	QSOX1	mRNA	-0.38	6.85E-06
413	227638	Qsox2	mRNA	-1.2849	1.17E-10	169714	QSOX2	mRNA	-0.8761	1.71E-13
414	227746	Rabepk	mRNA	-0.4677	0.0378315	10244	RABEPK	mRNA	-0.4849	0.00411
415	227624	Rabl6	mRNA	-0.6083	1.83E-05	55684	RABL6	mRNA	-0.25	0.01669
416	78255	Ralgps2	mRNA	-0.7682	0.0057502	55103	RALGPS2	mRNA	-0.2581	0.0349
417	98711	Rdh10	mRNA	-0.5946	0.0063364	157506	RDH10	mRNA	-1.7147	7.63E-28
418	320100	Relt	mRNA	-1.2651	1.12E-05	84957	RELT	mRNA	-1.4133	7.02E-27
419	76566	Rflnb	mRNA	-0.8583	1.28E-07	359845	RFLNB	mRNA	-0.7937	8.85E-17
420	328370	Rft1	mRNA	-1.2337	5.25E-09	91869	RFT1	mRNA	-0.4276	0.00043
421	19731	Rgl1	mRNA	-1.1386	0.000757	23179	RGL1	mRNA	-0.3648	0.01405
422	69288	Rhobtb1	mRNA	-0.9624	0.0025316	9886	RHOBTB1	mRNA	-1.6649	4.00E-53
423	67014	Riox2	mRNA	-1.3568	7.98E-12	84864	RIOX2	mRNA	-0.6387	3.96E-09
424	74315	Rnf145	mRNA	-1.3877	5.93E-10	153830	RNF145	mRNA	-1.4675	1.07E-39
425	269695	Rnft2	mRNA	-1.0838	0.000221	84900	RNFT2	mRNA	-1.0576	0.01357
426	26563	Ror1	mRNA	-2.0147	0.0041837	4919	ROR1	mRNA	-1.7694	1.92E-51
427	68925	Rpap1	mRNA	-0.6763	0.000903	26015	RPAP1	mRNA	-0.3997	0.01502
428	244585	Rpgrip1L	mRNA	-0.4759	0.0158979	23322	RPGRIP1L	mRNA	-0.4901	0.00064
429	52187	Rragd	mRNA	-0.8198	0.000773	58528	RRAGD	mRNA	-0.408	0.00256
430	18114	Rrp1	mRNA	-0.3323	0.0209718	8568	RRP1	mRNA	-0.9898	3.05E-19
431	72462	Rrp1B	mRNA	-0.3984	0.0028222	23076	RRP1B	mRNA	-1.0079	2.07E-22
432	74778	Rrp7A	mRNA	-0.5521	0.0013428	27341	RRP7A	mRNA	-0.7126	3.12E-14
433	170728	Rtn4Ip1	mRNA	-1.108	0.000412	84816	RTN4IP1	mRNA	-0.5585	0.02583
434	60455	Rxylt1	mRNA	-0.859	5.97E-05	10329	RXYLT1	mRNA	-0.7684	2.52E-07
435	72080	Sapcd2	mRNA	-0.8869	2.17E-05	89958	SAPCD2	mRNA	-0.6786	6.37E-08
436	216161	Sbno2	mRNA	-0.5808	0.0025348	22904	SBNO2	mRNA	-0.4577	0.00034
437	20778	Scarb1	mRNA	-0.516	0.0026763	949	SCARB1	mRNA	-0.5258	8.41E-06
438	12492	Scarb2	mRNA	-0.7632	8.87E-10	950	SCARB2	mRNA	-0.3497	2.72E-05
439	224024	Scarf2	mRNA	-0.5631	0.0136534	91179	SCARF2	mRNA	-1.4448	2.98E-18
440	74777	Selenon	mRNA	-1.1244	5.11E-09	57190	SELENON	mRNA	-0.2589	0.00637
441	20346	Sema3A	mRNA	-0.7154	0.000229	10371	SEMA3A	mRNA	-0.9669	0.00056
442	20349	Sema3E	mRNA	-0.4299	0.017823	9723	SEMA3E	mRNA	-1.9943	1.25E-07
443	20356	Sema5A	mRNA	-0.605	0.0070791	9037	SEMA5A	mRNA	-0.7982	8.64E-09
444	20361	Sema7A	mRNA	-0.9489	0.0069197	8482	SEMA7A	mRNA	-2.6399	1.11E-18
445	18787	Serpine1	mRNA	-2.2134	6.17E-19	5054	SERPINE1	mRNA	-2.6233	#######
446	101943	Sf3B3	mRNA	-0.2568	0.0180063	23450	SF3B3	mRNA	-0.6366	4.93E-18
447	78887	Sfi1	mRNA	-0.8894	0.002072	9814	SFI1	mRNA	-0.4046	0.02607
448	14057	Sfxn1	mRNA	-0.8973	4.50E-11	94081	SFXN1	mRNA	-0.6824	4.77E-13
449	94279	Sfxn2	mRNA	-1.0908	0.000285	118980	SFXN2	mRNA	-0.5167	0.00441
450	210274	Shank2	mRNA	-2.1297	0.0495495	22941	SHANK2	mRNA	-1.1643	6.01E-05
451	75698	Shld2	mRNA	-0.7289	0.007535	54537	SHLD2	mRNA	-0.2564	0.01374
452	18391	Sigmar1	mRNA	-0.8947	2.65E-09	10280	SIGMAR1	mRNA	-0.6453	1.16E-14
453	20469	Sipa1	mRNA	-0.8774	2.07E-09	6494	SIPA1	mRNA	-0.52	0.00219
454	76775	Slc10A7	mRNA	-0.7848	1.03E-05	84068	SLC10A7	mRNA	-0.6911	0.00105
455	18174	Slc11A2	mRNA	-0.607	0.000218	4891	SLC11A2	mRNA	-0.5393	9.90E-09
456	171286	Slc12A8	mRNA	-1.3071	0.0208305	84561	SLC12A8	mRNA	-0.4969	0.00629
457	83704	Slc12A9	mRNA	-0.6167	0.001025	56996	SLC12A9	mRNA	-0.3632	0.02185
458	80879	Slc16A3	mRNA	-2.3887	0.0080801	9123	SLC16A3	mRNA	-0.5371	1.29E-11

459	104681	Slc16A6	mRNA	-1.8207	0.000595	9120	SLC16A6	mRNA	-2.1713	9.10E-06
460	76306	Slc18B1	mRNA	-0.8524	0.002325	116843	SLC18B1	mRNA	-1.1147	1.66E-06
461	20509	Slc19A1	mRNA	-0.769	2.05E-06	6573	SLC19A1	mRNA	-1.5811	8.70E-34
462	20516	Slc20A2	mRNA	-0.6421	1.62E-05	6575	SLC20A2	mRNA	-1.8229	3.02E-44
463	18408	Slc25A15	mRNA	-0.5492	0.0256735	10166	SLC25A15	mRNA	-0.7377	4.33E-06
464	68267	Slc25A22	mRNA	-0.6428	0.008995	79751	SLC25A22	mRNA	-1.1566	2.98E-22
465	69906	Slc25A32	mRNA	-0.4213	0.0035913	81034	SLC25A32	mRNA	-0.8769	3.13E-16
466	63959	Slc29A1	mRNA	-0.5008	0.0040157	2030	SLC29A1	mRNA	-0.3219	0.01053
467	11416	Slc33A1	mRNA	-0.4721	0.0041674	9197	SLC33A1	mRNA	-0.4411	0.00021
468	73836	Slc35B2	mRNA	-0.5694	0.000106	347734	SLC35B2	mRNA	-0.2182	0.01857
469	14385	Slc37A4	mRNA	-1.3237	1.21E-12	2542	SLC37A4	mRNA	-0.4869	2.81E-07
470	625098	Slc38A6	mRNA	-1.4199	5.65E-07	145389	SLC38A6	mRNA	-0.4474	0.03335
471	68427	Slc39A13	mRNA	-1.0129	1.19E-07	91252	SLC39A13	mRNA	-0.4476	3.59E-07
472	213053	Slc39A14	mRNA	-0.5873	0.0342551	23516	SLC39A14	mRNA	-0.3453	0.00083
473	106947	Slc39A3	mRNA	-0.729	0.0051825	29985	SLC39A3	mRNA	-0.7911	9.96E-10
474	52466	Slc46A1	mRNA	-1.2838	0.00019	113235	SLC46A1	mRNA	-2.0106	2.35E-27
475	71706	Slc46A3	mRNA	-1.894	0.0150911	283537	SLC46A3	mRNA	-1.0987	1.55E-29
476	52710	Slc52A2	mRNA	-0.9575	5.42E-05	79581	SLC52A2	mRNA	-0.5266	8.34E-08
477	330064	Slc5A6	mRNA	-1.2078	3.12E-10	8884	SLC5A6	mRNA	-1.1789	2.54E-21
478	236727	Slc9A7	mRNA	-0.638	0.000133	84679	SLC9A7	mRNA	-0.3165	0.00988
479	108116	Slco3A1	mRNA	-0.5476	0.0374711	28232	SLCO3A1	mRNA	-0.339	0.01226
480	20587	Smarcb1	mRNA	-0.4074	0.0061444	6598	SMARCB1	mRNA	-0.2665	0.0135
481	66993	Smarcd3	mRNA	-0.438	0.0300506	6604	SMARCD3	mRNA	-0.4219	0.01707
482	319757	Smo	mRNA	-0.607	1.39E-05	6608	SMO	mRNA	-1.9085	1.89E-15
483	29856	Smtn	mRNA	-0.5424	0.000229	6525	SMTN	mRNA	-0.422	0.00023
484	232187	Smyd5	mRNA	-0.3728	0.036205	10322	SMYD5	mRNA	-0.7193	5.33E-09
485	102607	Snx19	mRNA	-0.9936	3.21E-11	399979	SNX19	mRNA	-0.2607	0.00238
486	20652	Soat1	mRNA	-0.8984	1.28E-08	6646	SOAT1	mRNA	-0.5325	7.02E-11
487	109205	Sobp	mRNA	-2.2144	0.0148374	55084	SOBP	mRNA	-0.9846	0.01878
488	81840	Sorcs2	mRNA	-0.9037	0.0225271	57537	SORCS2	mRNA	-0.7	0.03039
489	20692	Sparc	mRNA	-0.494	0.0388047	6678	SPARC	mRNA	-0.4162	0.00021
490	219140	Spata13	mRNA	-1.0438	0.0017943	221178	SPATA13	mRNA	-0.8098	3.09E-07
491	11790	Speg	mRNA	-0.5136	0.0097062	10290	SPEG	mRNA	-0.5261	0.00046
492	78586	Srbd1	mRNA	-0.4404	0.0081001	55133	SRBD1	mRNA	-0.3617	0.0184
493	20383	Srsf3	mRNA	-0.8389	0.000838	6428	SRSF3	mRNA	-0.765	3.08E-15
494	225027	Srsf7	mRNA	-0.8551	2.14E-05	6432	SRSF7	mRNA	-0.9957	2.90E-29
495	107513	Ssr1	mRNA	-0.3267	0.0147395	6745	SSR1	mRNA	-0.5041	3.02E-11
496	243362	Stard13	mRNA	-0.9379	0.000149	90627	STARD13	mRNA	-1.6863	6.20E-50
497	170459	Stard4	mRNA	-0.4006	0.0365765	134429	STARD4	mRNA	-0.5272	1.54E-08
498	20846	Stat1	mRNA	-1.6241	1.72E-11	6772	STAT1	mRNA	-0.4427	1.76E-07
499	20851	Stat5B	mRNA	-0.6093	0.00068	6777	STAT5B	mRNA	-0.3739	0.00743
500	68428	Steap3	mRNA	-1.4839	1.46E-06	55240	STEAP3	mRNA	-0.8292	2.58E-22
501	53416	Stk39	mRNA	-0.5611	0.021424	27347	STK39	mRNA	-0.3054	0.00655
502	69106	Stoml1	mRNA	-1.808	1.01E-09	9399	STOML1	mRNA	-0.3583	0.01178
503	68292	Stt3B	mRNA	-0.262	0.0332391	201595	STT3B	mRNA	-0.4858	2.25E-10
504	217517	Stxbp6	mRNA	-1.5378	6.49E-10	29091	STXBP6	mRNA	-0.5996	0.00129
505	234373	Sugp2	mRNA	-0.651	0.0076695	10147	SUGP2	mRNA	-0.2879	0.01621
506	77053	Sun1	mRNA	-0.3604	0.00776	23353	SUN1	mRNA	-0.2759	0.00634
507	20973	Syngr2	mRNA	-0.4534	0.0023195	9144	SYNGR2	mRNA	-0.3741	5.02E-06
508	233335	Synm	mRNA	-1.3858	0.0028187	23336	SYNM	mRNA	-1.8326	1.34E-30
509	217030	Synrg	mRNA	-1.0235	1.55E-06	11276	SYNRG	mRNA	-0.3741	0.00056
510	272538	Tango6	mRNA	-0.7063	6.59E-05	79613	TANGO6	mRNA	-1.1597	9.80E-14
511	71807	Tars2	mRNA	-0.7213	2.74E-06	80222	TARS2	mRNA	-0.6268	3.18E-06
512	544696	Tbc1D32	mRNA	-0.6281	0.0065148	221322	TBC1D32	mRNA	-0.6575	0.03928
513	210789	Tbc1D4	mRNA	-1.5061	6.32E-11	9882	TBC1D4	mRNA	-1.025	1.94E-10
514	76795	Tbc1D9B	mRNA	-0.9386	4.74E-09	23061	TBC1D9B	mRNA	-0.2179	0.01411
515	27368	Tbl2	mRNA	-0.474	0.0010657	26608	TBL2	mRNA	-0.3909	0.0004
516	77832	Tchp	mRNA	-0.8056	0.000249	84260	TCHP	mRNA	-0.4959	0.00043

517	27060	Tcirg1	mRNA	-0.7937	4.94E-09	10312	TCIRG1	mRNA	-0.4345	0.00067
518	67590	Tctn3	mRNA	-1.2066	1.36E-08	26123	TCTN3	mRNA	-0.3396	0.00035
519	104884	Tdp1	mRNA	-0.6723	6.26E-05	55775	TDP1	mRNA	-0.2972	0.00825
520	71718	Telo2	mRNA	-0.6807	0.000152	9894	TELO2	mRNA	-0.6021	1.69E-06
521	21766	Tex261	mRNA	-0.4672	0.001025	113419	TEX261	mRNA	-0.4007	5.24E-07
522	21808	Tgfb2	mRNA	-1.2552	3.97E-07	7042	TGFB2	mRNA	-3.0202	5.48E-38
523	21813	Tgfbr2	mRNA	-0.866	3.64E-09	7048	TGFBR2	mRNA	-0.4166	0.0005
524	21814	Tgfbr3	mRNA	-0.5712	0.0109618	7049	TGFBR3	mRNA	-0.4062	0.0448
525	73122	Tgfbrap1	mRNA	-0.4626	0.0478403	9392	TGFBRAP1	mRNA	-0.5887	1.14E-09
526	21817	Tgm2	mRNA	-0.4458	0.0363986	7052	TGM2	mRNA	-0.7885	4.05E-13
527	240174	Thada	mRNA	-1.0297	8.35E-12	63892	THADA	mRNA	-0.9168	1.65E-14
528	223626	Them6	mRNA	-1.671	4.53E-25	51337	THEM6	mRNA	-0.8827	6.30E-10
529	50492	Thop1	mRNA	-0.3784	0.0341813	7064	THOP1	mRNA	-0.4713	2.59E-05
530	105663	Thtpa	mRNA	-0.4681	0.0129508	79178	THTPA	mRNA	-0.49	0.01735
531	72167	Thumpd2	mRNA	-0.6933	0.0039161	80745	THUMPD2	mRNA	-0.4301	0.02771
532	24001	Tiam2	mRNA	-0.6738	0.0070342	26230	TIAM2	mRNA	-0.699	2.05E-05
533	68140	Tigd2	mRNA	-0.5922	0.0017927	166815	TIGD2	mRNA	-0.5017	0.02461
534	14356	Timm10B	mRNA	-0.4373	0.0169126	26515	TIMM10B	mRNA	-0.2638	0.0273
535	94242	Tinagl1	mRNA	-0.7078	1.31E-05	64129	TINAGL1	mRNA	-0.7447	0.00801
536	68385	Tlcd1	mRNA	-1.0563	2.85E-10	116238	TLCD1	mRNA	-0.7726	0.00051
537	70549	Tln2	mRNA	-0.8298	0.000133	83660	TLN2	mRNA	-0.8411	8.92E-13
538	320534	Tmem104	mRNA	-1.1171	1.54E-06	54868	TMEM104	mRNA	-0.8671	1.69E-12
539	217203	Tmem106	mRNA	-1.2132	2.94E-05	113277	TMEM106A	mRNA	-1.36	1.58E-06
540	68539	Tmem109	mRNA	-0.3957	0.0087398	79073	TMEM109	mRNA	-0.49	7.08E-09
541	229473	Tmem131	mRNA	-0.9802	0.000283	23240	TMEM131L	mRNA	-0.7155	2.09E-06
542	72982	Tmem138	mRNA	-0.4589	0.0098403	51524	TMEM138	mRNA	-0.6189	2.99E-08
543	72309	Tmem158	mRNA	-0.6717	0.022846	25907	TMEM158	mRNA	-1.2457	1.54E-13
544	103765	Tmem17	mRNA	-1.4352	0.0014329	200728	TMEM17	mRNA	-0.5995	0.04768
545	66343	Tmem177	mRNA	-0.6911	0.0070461	80775	TMEM177	mRNA	-0.9526	1.46E-06
546	230917	Tmem201	mRNA	-0.7073	1.98E-05	199953	TMEM201	mRNA	-1.642	1.22E-27
547	68796	Tmem214	mRNA	-0.3222	0.0347352	54867	TMEM214	mRNA	-0.7431	2.73E-19
548	68642	Tmem216	mRNA	-0.6076	0.0048044	51259	TMEM216	mRNA	-0.4705	0.018
549	77975	Tmem50B	mRNA	-0.4249	0.0493074	757	TMEM50B	mRNA	-0.54	0.00024
550	70397	Tmem70	mRNA	-0.4115	0.016492	54968	TMEM70	mRNA	-0.3862	0.00466
551	69071	Tmem97	mRNA	-0.32	0.0331736	27346	TMEM97	mRNA	-0.2381	0.01373
552	21961	Tns1	mRNA	-1.6515	2.03E-10	7145	TNS1	mRNA	-0.8993	0.00086
553	252972	Tpcn1	mRNA	-0.8249	2.84E-05	53373	TPCN1	mRNA	-0.5537	4.08E-09
554	22031	Traf3	mRNA	-0.434	0.0468215	7187	TRAF3	mRNA	-0.4081	0.00023
555	22033	Traf5	mRNA	-1.2928	0.00017	7188	TRAF5	mRNA	-0.6773	7.34E-07
556	22670	Trim26	mRNA	-0.9251	1.78E-07	7726	TRIM26	mRNA	-0.3324	0.00493
557	69716	Trip13	mRNA	-0.707	0.000197	9319	TRIP13	mRNA	-0.263	0.042
558	15547	Trmt2A	mRNA	-0.6044	0.000109	27037	TRMT2A	mRNA	-0.5179	3.57E-05
559	215201	Trmt2B	mRNA	-0.5363	0.0060899	79979	TRMT2B	mRNA	-0.3531	0.00972
560	328162	Trmt61A	mRNA	-0.5802	0.000427	115708	TRMT61A	mRNA	-0.6868	4.78E-08
561	381802	Tsen2	mRNA	-1.807	2.47E-10	80746	TSEN2	mRNA	-0.9219	3.06E-07
562	269831	Tspan12	mRNA	-0.614	0.0195238	23554	TSPAN12	mRNA	-0.8562	3.15E-05
563	56224	Tspan5	mRNA	-0.5816	0.000167	10098	TSPAN5	mRNA	-0.7831	1.13E-12
564	72480	Tspyl4	mRNA	-1.4607	0.0035804	23270	TSPYL4	mRNA	-0.8067	5.17E-18
565	74044	Ttf2	mRNA	-0.5134	0.008279	8458	TTF2	mRNA	-0.2655	0.01605
566	75425	Tti1	mRNA	-0.659	0.0081511	9675	TTI1	mRNA	-0.6228	2.66E-08
567	223723	Ttll12	mRNA	-0.5494	0.000504	23170	TTLL12	mRNA	-1.4504	1.16E-43
568	50500	Ttpa	mRNA	-1.1977	0.0111694	7274	TTPA	mRNA	-1.0966	0.02769
569	106200	Txndc11	mRNA	-0.4465	0.0083302	51061	TXNDC11	mRNA	-0.4078	5.17E-05
570	54721	Tyk2	mRNA	-0.5539	0.006656	7297	TYK2	mRNA	-0.3843	0.00673
571	22174	Tyro3	mRNA	-0.4747	0.0076576	7301	TYRO3	mRNA	-0.762	2.04E-08
572	71767	Tysnd1	mRNA	-1.8813	1.43E-09	219743	TYSND1	mRNA	-0.8402	0.0001
573	100929	Tyw1	mRNA	-0.4246	0.0013021	55253	TYW1	mRNA	-0.3918	0.00188
574	50995	Uba2	mRNA	-0.403	0.0013603	10054	UBA2	mRNA	-0.35	4.24E-05

575	72828	Ubash3B	mRNA	-1.0389	2.25E-08	84959	UBASH3B	mRNA	-1.2782	8.83E-60
576	67196	Ube2T	mRNA	-0.8108	7.28E-05	29089	UBE2T	mRNA	-0.3391	0.02854
577	320011	Uggt1	mRNA	-0.2755	0.0377339	56886	UGGT1	mRNA	-0.6005	3.10E-10
578	107197	Uqcc3	mRNA	-0.6296	0.0073765	790955	UQCC3	mRNA	-0.4501	0.00735
579	24110	Usp18	mRNA	-2.5976	0.000835	11274	USP18	mRNA	-1.0172	0.03257
580	227334	Usp40	mRNA	-0.7449	0.000448	55230	USP40	mRNA	-0.3149	0.00098
581	229658	Vangl1	mRNA	-1.0788	1.10E-07	81839	VANGL1	mRNA	-0.2855	0.00148
582	74048	Vsir	mRNA	-3.5069	0.0065975	64115	VSIR	mRNA	-0.7714	8.90E-07
583	57750	Wdr12	mRNA	-0.4363	0.0260577	55759	WDR12	mRNA	-0.5943	1.83E-08
584	269470	Wdr3	mRNA	-0.346	0.0341455	10885	WDR3	mRNA	-1.0345	5.06E-26
585	74682	Wdr35	mRNA	-0.4563	0.0136666	57539	WDR35	mRNA	-0.3521	0.01522
586	72338	Wdr89	mRNA	-0.9938	0.004644	112840	WDR89	mRNA	-0.4251	0.01727
587	57258	Xpo4	mRNA	-0.4023	0.0037985	64328	XPO4	mRNA	-0.5718	1.51E-07
588	268880	Xxylt1	mRNA	-1.4968	8.95E-15	152002	XXYLT1	mRNA	-0.8118	9.48E-11
589	102448	Xylb	mRNA	-0.7708	0.0189432	9942	XYLB	mRNA	-1.2758	1.22E-10
590	240255	Ythdc2	mRNA	-0.4545	0.0327813	64848	YTHDC2	mRNA	-0.5035	8.09E-06
591	330474	Zc3H4	mRNA	-0.415	0.0485521	23211	ZC3H4	mRNA	-0.2888	0.03248
592	74168	Zdhhc16	mRNA	-0.3328	0.0155364	84287	ZDHHC16	mRNA	-0.4774	1.09E-06
593	70605	Zdhhc24	mRNA	-1.568	2.26E-05	254359	ZDHHC24	mRNA	-0.2778	0.04146
594	208884	Zdhhc9	mRNA	-0.7233	0.00036	51114	ZDHHC9	mRNA	-0.5068	1.36E-06
595	195018	Zzef1	mRNA	-1.132	2.62E-07	23140	ZZEF1	mRNA	-0.3406	0.00318
